# Catalytic Decomposition of Long‐Chain Olefins to Propylene via Isomerization‐Metathesis Using Latent Bicyclic (Alkyl)(Amino)Carbene‐Ruthenium Olefin Metathesis Catalysts

**DOI:** 10.1002/anie.202204413

**Published:** 2022-05-19

**Authors:** Márton Nagyházi, Ádám Lukács, Gábor Turczel, Jenő Hancsók, József Valyon, Attila Bényei, Sándor Kéki, Róbert Tuba

**Affiliations:** ^1^ Institute of Materials and Environmental Chemistry Eötvös Loránd Research Network Research Centre for Natural Sciences Magyar tudósok körútja 2 1519 Budapest Hungary; ^2^ Department of Organic Chemistry and Technology Budapest University of Technology and Economics Szent Gellért tér 4 1111 Budapest Hungary; ^3^ Research Centre for Biochemical Environmental and Chemical Engineering Department of MOL Hydrocarbon and Coal Processing University of Pannonia Egyetem u. 10 8210 Veszprém Hungary; ^4^ Department of Pharmaceutical Chemistry Faculty of Science and Technology University of Debrecen Egyetem tér 1 4032 Debrecen Hungary; ^5^ Department of Applied Chemistry Faculty of Science and Technology University of Debrecen Egyetem tér 1 4032 Debrecen Hungary

**Keywords:** BICAAC, Hydrocarbon Decomposition, ISOMET, Metathesis, Propylene, Ruthenium

## Abstract

One of the most exciting scientific challenges today is the catalytic degradation of non‐biodegradable polymers into value‐added chemical feedstocks. The mild pyrolysis of polyolefins, including high‐density polyethylene (HDPE), results in pyrolysis oils containing long‐chain olefins as major products. In this paper, novel bicyclic (alkyl)(amino)carbene ruthenium (BICAAC−Ru) temperature‐activated latent olefin metathesis catalysts, which can be used for catalytic decomposition of long‐chain olefins to propylene are reported. These thermally stable catalysts show significantly higher selectivity to propylene at a reaction temperature of 75 °C compared to second generation Hoveyda–Grubbs or CAAC−Ru catalysts under ethenolysis conditions. The conversion of long‐chain olefins (e.g., 1‐octadecene or methyl oleate) to propylene via isomerization‐metathesis is performed by using a (RuHCl)(CO)(PPh_3_)_3_ isomerization co‐catalyst. The reactions can be carried out at a BICAAC−Ru catalyst loading as low as 1 ppm at elevated reaction temperature (75 °C). The observed turnover number and turnover frequency are as high as 55 000 and 10 000 mol_propylene_ mol_catalyst_^−1^ h^−1^, respectively.

## Graphical abstract

Bicyclic (alkyl)(amino)carbene ruthenium temperature‐activated, thermally stable olefin metathesis (ethenolysis) catalysts are reported for the catalytic decomposition of long‐chain olefins such as 1‐octadecene or methyl oleate into propylene. The reaction occurs via isomerization‐metathesis using the (RuHCl)(CO)(PPh_3_)_3_ isomerization co‐catalyst.

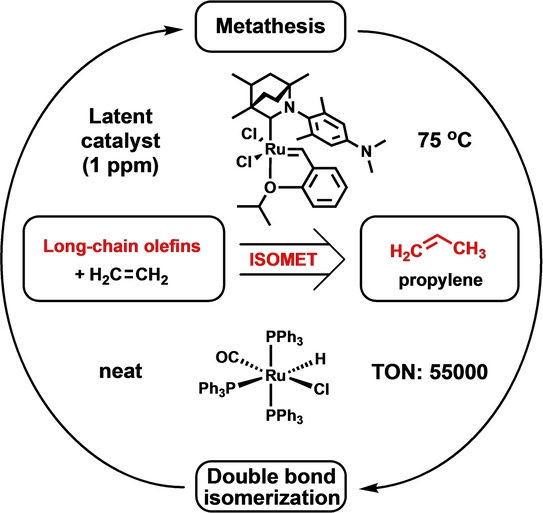

The industrial application of sustainable catalysis is crucial from the point of view of contemporary chemical technologies.[^1^, ^2^, ^3^] For example, the selective catalytic decomposition of non‐biodegradable polymers, the cause of microplastic pollution, such as polyethylene (PE) (produced 100 million metric tons in 2018) to high value chemical feedstock is an urgent issue in contemporary research.^[4]^ For instance, the transformation of PE waste into propylene—the second largest volume petrochemical (after ethylene) produced today—would be a major step forward to the valorization of non‐biodegradable polymer waste. Currently, propylene is mainly produced from crude oil via steam cracking as by‐product of ethylene production[^5^, ^6^] and via olefin metathesis of 2‐butene.[^7^, ^8^, ^9^, ^10^] Although there is a growing demand for its sustainable production, its economically viable synthesis from plastic waste or renewable resources is not yet established.^[11]^

The application of olefin metathesis in the field of green chemistry, especially in sustainable catalysis, is emerging.[^12^, ^13^, ^14^, ^15^] A special case of olefin metathesis is called isomerization‐metathesis (ISOMET). In the case of linear mono‐olefins, the complete ISOMET using ethylene as the cross‐coupling agent theoretically ends up with propylene as the only product regardless of the position of the double bond and the length of the hydrocarbon chain (Scheme 1).

**Scheme 1:**
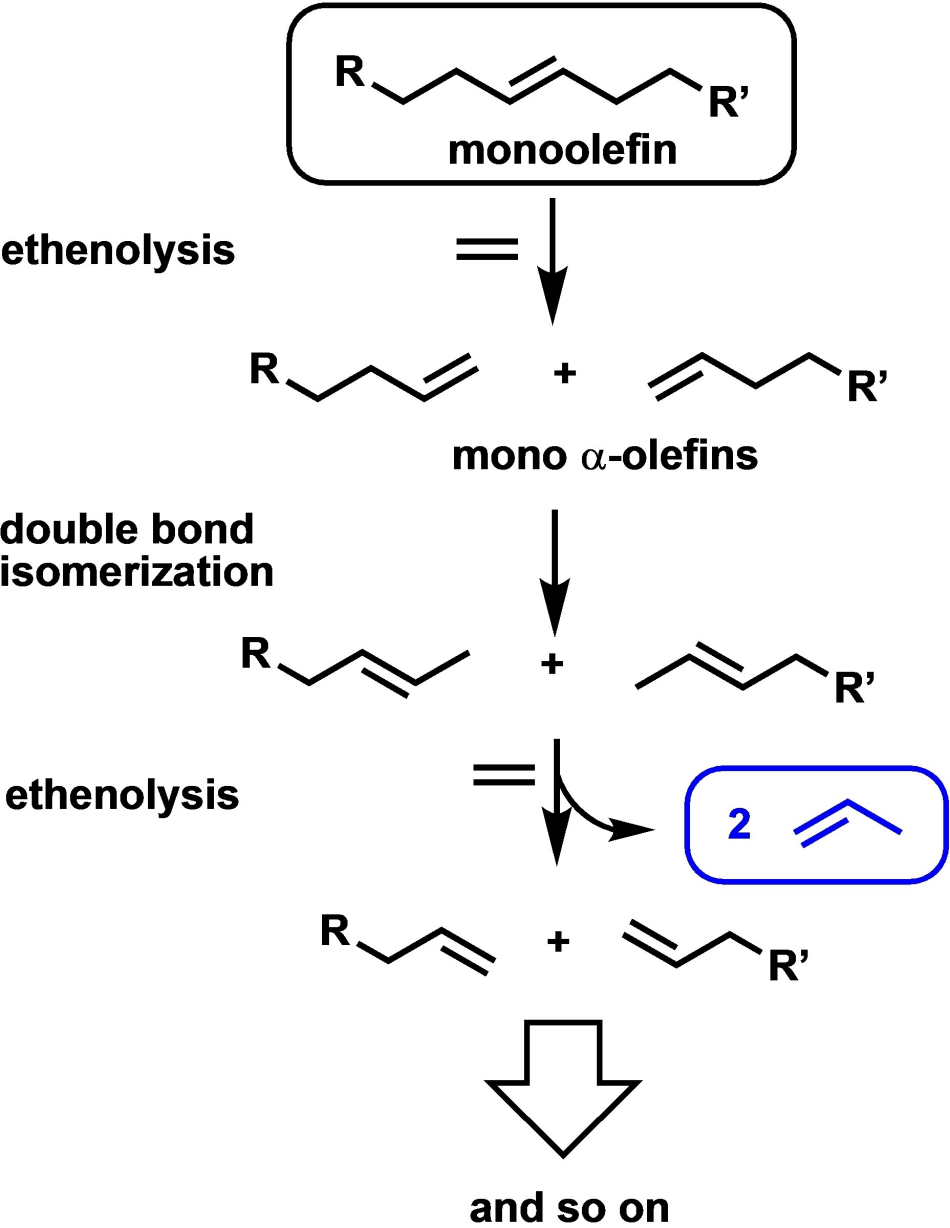
Tentative procedure for propylene production from high‐molecular‐weight mono‐olefins.

As most of the isomerization catalysts including RuHCl(CO)(PPh_3_)_3_ (**RuH**) operate at elevated temperature (>50 °C), the ISOMET reactions necessitate an ethenolysis catalyst showing improved thermal stability.^[15]^ The ISOMET reaction using mixed heterogeneous MoO_3_/Al_2_O_3_ and H‐BEA zeolite systems has recently been demonstrated in our laboratory.^[16]^ The drawbacks of the reaction are, however, the low turnover number (TON) and short catalyst lifetime even at relatively high molybdena coverage.^[17]^

The application of latent catalysts with olefin metathesis activity is emerging.[^18^, ^19^, ^20^] The key feature of the latent catalysts involves low activity or inactivity at ambient conditions; meanwhile these catalysts can be converted into to a highly active form upon external stimuli such as chemical, photochemical, or thermal activation.^[21]^ One of the main advantages of the temperature‐activated latent activity is, for example, that the reaction can be set up at room temperature without any undesired side reactions. For instance, in the case of ethenolysis, olefin self‐metathesis can be avoided when there is still no ethylene pressure applied. The heat‐activated olefin metathesis catalyst, however, in general requires relatively high loading, as the formed active species exhibit limited stability at elevated temperatures. This is especially true for one of the most temperature‐sensitive catalytic intermediates of ethenolysis, ruthenium methylidene species (Ru=CH_2_).[^22^, ^23^] These intermediates, when heated in the presence of ethylene excess, may take part in β‐elimination reactions and lead to fast intrinsic catalyst decomposition.[^24^, ^25^, ^26^]

The recent development of new carbene ligands has resulted in catalysts with higher stability, functional group tolerance, and improved catalytic activity (Scheme 2).[^27^, ^28^, ^29^, ^30^, ^31^, ^32^] For example, replacing the N‐heterocyclic carbene (NHC) ligand of the second generation Hoveyda–Grubbs catalyst (**HG2**) by CAAC‐5 carbenes significantly improves the efficiency of olefin metathesis (Scheme 3, **BG**).[^33^, ^34^, ^35^] Exceptionally high methyl oleate (**24**) ethenolysis activity (340 000 TON) has been reported by Bertrand and Grubbs.^[13]^ A highly active *bis*(CAAC‐5)‐Ru olefin metathesis catalyst (**3**) is also known.^[34]^ We have recently reported the synthesis of quaternary ammonium ion tagged derivatives of complex **2** revealing remarkably better stability than analogs of first and second generation ruthenium‐based metathesis catalysts (Scheme 3). These complexes have shown exceptional activity in olefin metathesis even in protic media.^[36]^ Ruthenium complexes (e.g. **4** and **5**, Scheme 3) containing six‐membered cyclic and bicyclic alkyl amino carbene ligands (CAAC‐6, BICAAC‐6, Scheme 2) are also known. However, they display lower olefin metathesis activity at ambient reaction conditions.[^37^, ^42^] The modification of the ring allowed the synthesis of bicyclic carbenes (BICAAC‐6) with favorable electronic and steric properties (Scheme 2).^[28]^ Although some transition metal complexes of BICAAC ligands have recently been reported, their ruthenium derivatives are rare.[^38^, ^39^, ^40^, ^41^, ^42^]

**Scheme 2:**
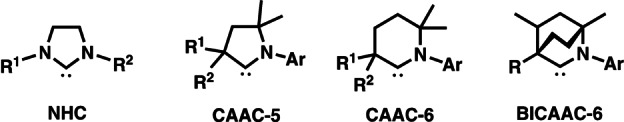
Representative examples for carbene ligands used for homogeneous catalysis.

**Scheme 3:**
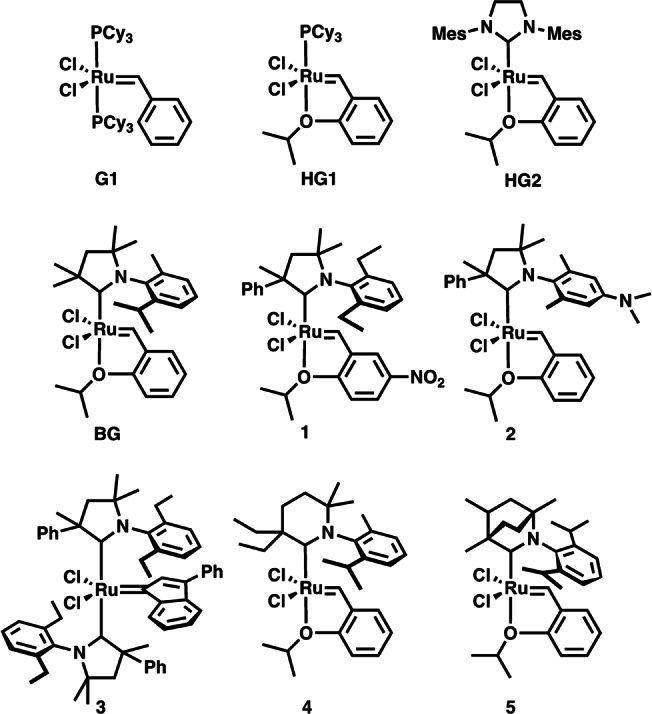
Representative examples for ruthenium‐based olefin metathesis catalysts (PCy_3_=tricyclohexylphosphine; Mes=mesityl).

In this paper, a novel, robust, highly active BICAAC−Ru olefin metathesis and ISOMET catalyst system showing temperature‐activated latent activity is reported.

BICAAC ruthenium catalyst synthesis and characterization: Ligands **10**–**13** were synthetized by slightly modified literature procedures (Scheme 4, see the Supporting Information).[^28^, ^41^] The BICAAC salts were deprotonated, giving the respective carbenes. It was found that the phosphine–carbene ligand exchange takes place readily with both **G1** and **HG1** complexes. Reactions of the in situ formed carbenes with **G1** and **HG1** afforded the target molecules **14**, **16**, **18**–**20** (Scheme 5).

**Scheme 4:**
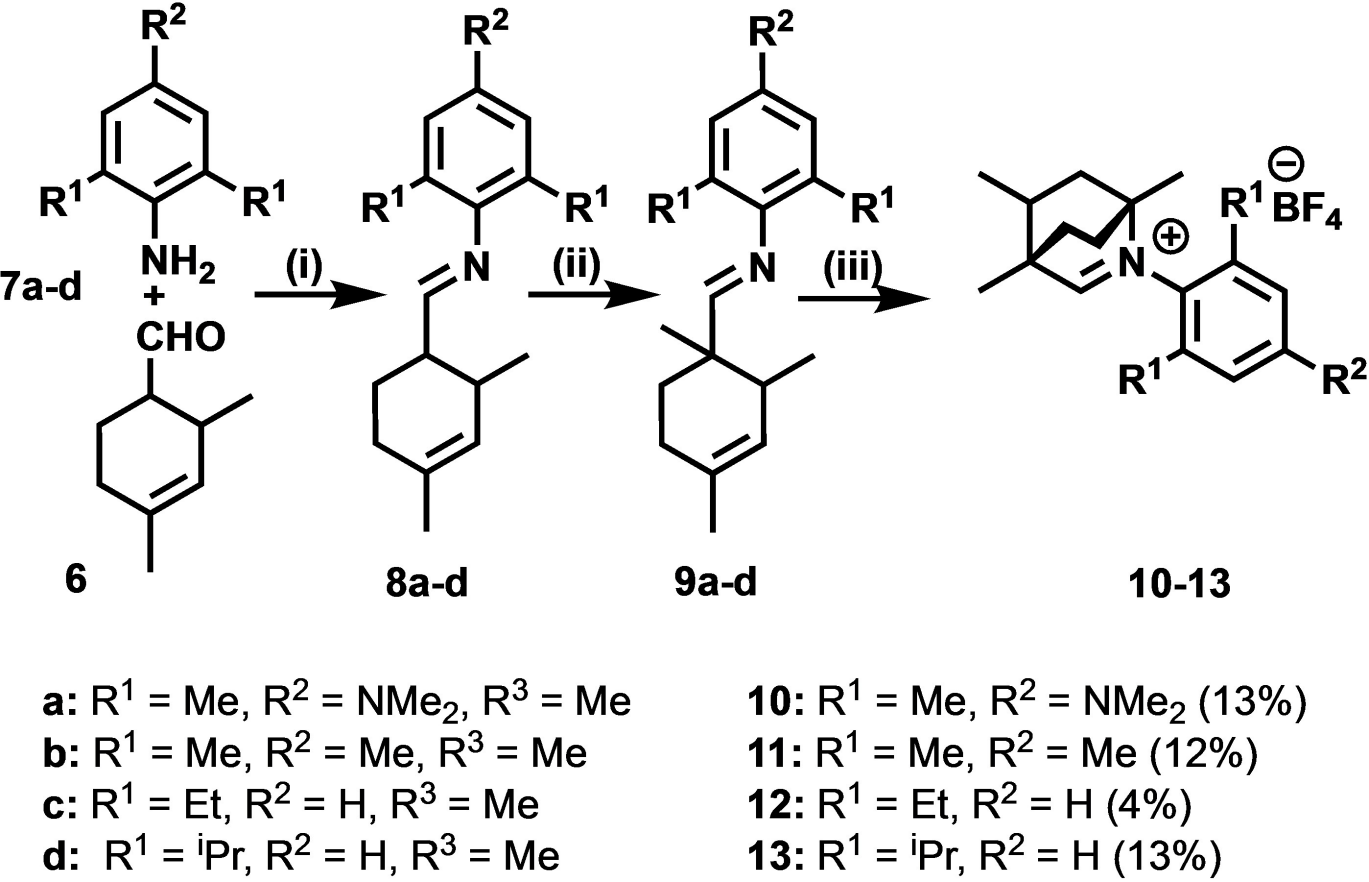
A representative example for the synthesis of BICAAC carbene precursor salts **10**–**13**. Conditions: (i) DCM, room temperature, molecular sieves (3 Å); (ii) 2 equiv LDA, dry THF, 2 equiv methyl iodide, 0 °C, (iii) 3–6 equiv HCl in dioxane (3 M), 80 °C, NH_4_BF_4_.

**Scheme 5:**
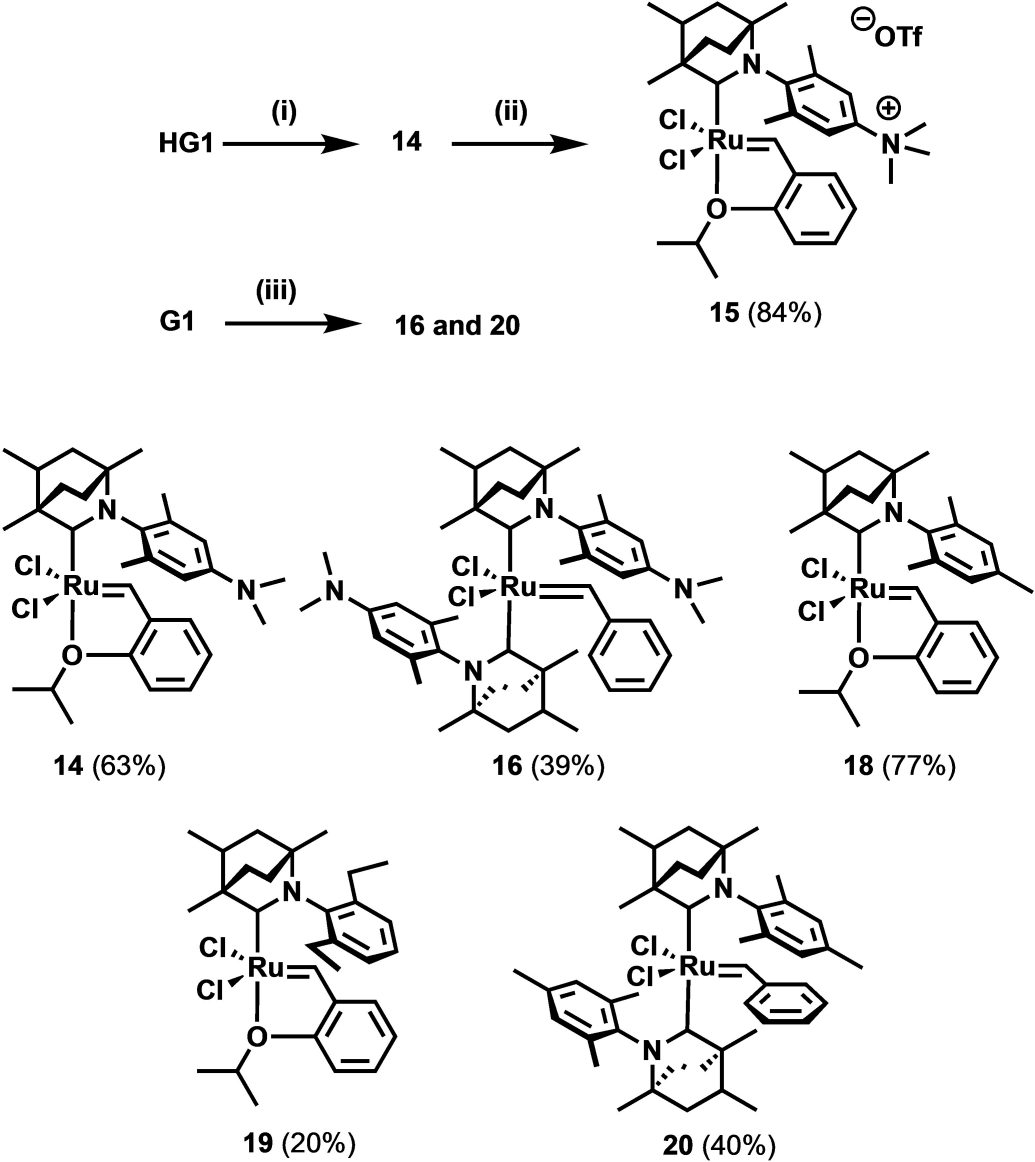
Synthesis of BICAAC ruthenium carbene complexes. Conditions: (i) 1.5 equiv of carbene precursors **10**–**13**, 1.5 equiv LiHMDS, THF, 25 °C; (ii) 1.1 equiv of MeOTf, DCM, −30 °C; (iii) 3.0 equiv of carbene precursors **10** and **11**, 3.3 equiv LiHMDS, THF, 25 °C.

*Mono*‐BICAAC complexes like **14** were very stable in air, even in solution and during chromatography. Meanwhile, *bis*‐BICAAC complexes such as **16**, which can also be purified under air, showed lower shelf‐life stability under ambient conditions. The lower stability is presumably caused by the strong *trans*‐effect of the BICAAC ligands. This effect is well known for both NHC^[43]^ and CAAC^[25]^ systems. Complexes **14** and **16** were alkylated with methyl trifluoromethanesulfonate (MeOTf). While complex **15** could be isolated in reasonable yield and the product could be analyzed by X‐ray diffraction (see Figure S36, Table S1), the alkylation of **16** gave a multicomponent mixture. In the ^1^H NMR spectrum there was some indication of the formation of the alkylated compound (**17**, see the Supporting Information); however, its isolation failed. Complex **15** showed high stability and solubility in protic solvents (Scheme 5). NMR investigations revealed that although several ^1^H benzylidene signals were present in the crude mixture after complexation of mono‐carbene complexes—due to the racemic nature of the BICAAC ligands,—only one major diastereomer can be observed after purification (recrystallization or chromatography). The role of the possible complex diastereomer pairs formed using asymmetric carbene ligand has already been discussed in our recent article.^[36]^ Single crystals suitable for X‐ray diffraction analysis (Figure 1) were obtained by concentration of the acetone solution of the complexes by slow evaporation, or by gentle cooling of their concentrated hexane solution (for details see the Supporting Information).^[44]^

**Figure 1:**
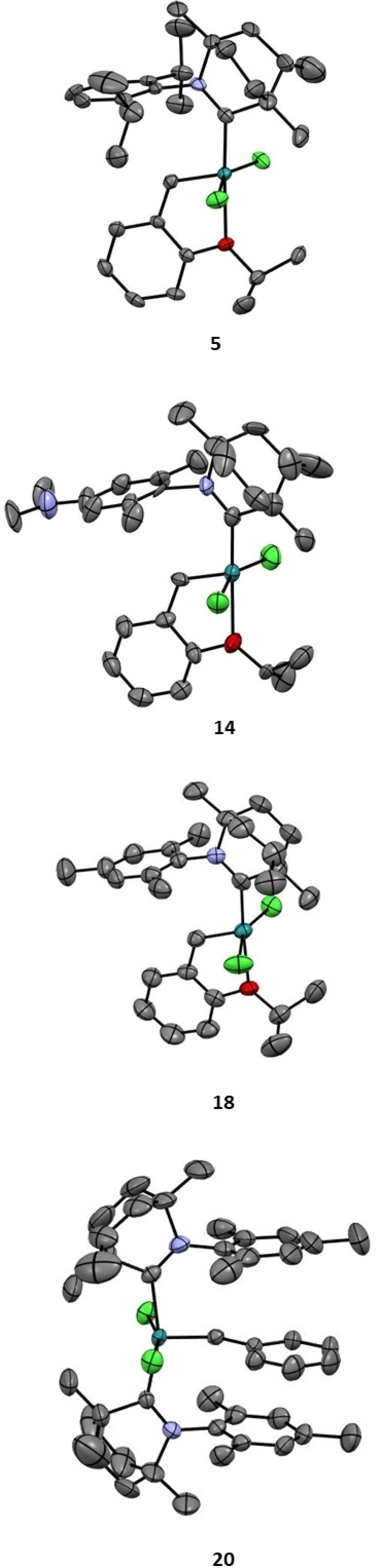
X‐ray crystal structures of complexes **5**, **14**, **18** and **20**.^[44]^

Investigation of the latent olefin metathesis reaction: The ring‐closing metathesis (RCM) reaction of diallyl diethyl malonate^[45]^ (**21**) using catalysts **14**, **18**–**20** was investigated (Scheme 6) at reaction temperatures of 50 and 75 °C by in situ ^1^H NMR spectroscopy. When 0.05 mol % of catalyst **14** was used, the reaction carried out at 50 °C resulted in the formation of the RCM product (**22**) in very low yield (2 %) within three hours. However, when the reaction was repeated at 75 °C, as high as 80 % yield of **22** was achieved within the same reaction time (Figure S38). The formal kinetic description of the formation of **22** can be found in the Supporting Information. In an additional experiment, complexes **14**, **18**–**20** were investigated at 0.25 mol % catalyst loading: the mixture was heated at 50 °C for one hour, then the temperature was increased to 75 °C for an additional five hours (Figure 2). Very low RCM product yield was observed for each catalyst after the first hour at 50 °C. However, when the reaction temperature was raised to 75 °C, the RCM reactions started immediately. With catalyst **14** after 2 hours at 75 °C more than 90 % yield of **22** and almost complete conversion of **21** was observed. The *bis*‐carbene catalyst **20** also showed high activity; however, after two hours at 75 °C, when 80 % yield of the product was reached, a plateau indicating catalyst decomposition was observed. In the case of catalyst **18**, 90 % yield was achieved after six hours. Catalyst **19** gave a slightly lower yield of 80 % in the same reaction time frame; however, the reaction was not yet complete, indicating lower activity but good robustness of the catalyst. Finally, it could be concluded that both the monocarbene (**14**, **18**, and **19**) and *bis*‐carbene (**20**) complexes show unequivocal latent properties.

**Scheme 6:**
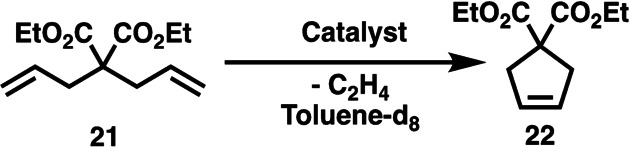
Ring‐closing metathesis (RCM) model reaction using catalyst **14**, **18**–**20**.

**Figure 2:**
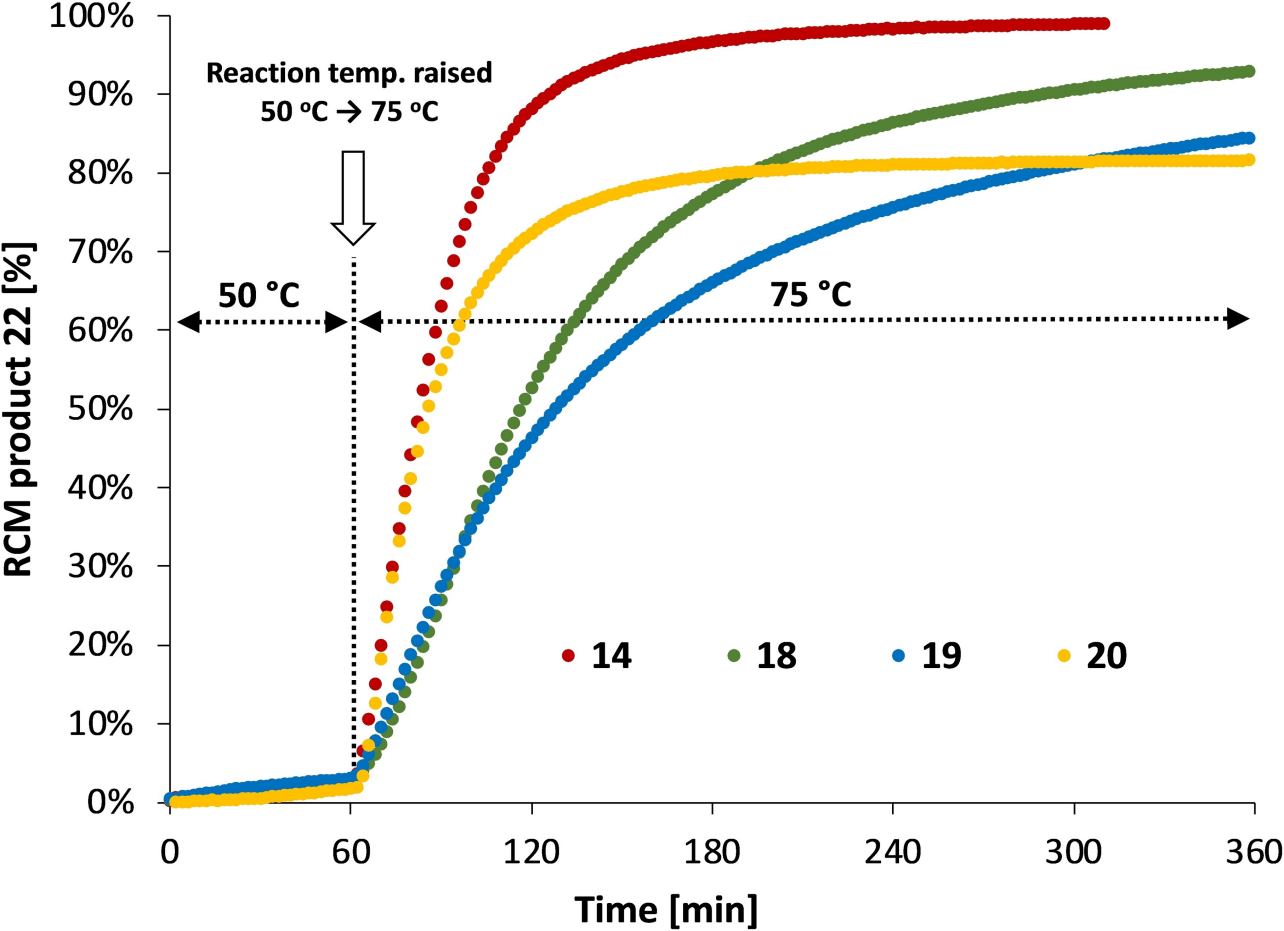
Comparison of the latent activity of BICAAC−Ru complexes. *Mono‐*carbene complexes: **14** (red), **18** (green), **19** (blue), *bis‐*carbene complex: **20** (orange). 50 °C for 60 min, then 75 °C for 300 min; toluene‐d_8_; [**21**]=0.20 M; catalyst loading: 0.25 mol %.

Interestingly, no RCM reaction was observed with catalyst **5**. Even increasing the reaction temperature to 100 °C resulted in a minor RCM product yield of only 3 % over a longer reaction time (24 h). Regarding the reaction curves, a correlation can be seen between the steric hindrance of the alkyl groups of the N‐aryl moiety in the *ortho* position and the catalytic activity of the BICAACRu complexes. RCM reaction in methanol using ionized complex **15** under similar conditions showed catalyst decomposition; no RCM product formation was observed.

ISOMET of methyl oleate (**23**): Due to the latent properties and increased catalytic activity of the BICAAC−Ru complexes at elevated temperatures (75 °C), our attention was focused on their ISOMET reactions. Since the isomerization activity of the catalyst (RuHCl(CO)(PPh_3_)_3_ (**RuH**) increases at elevated temperatures (above 50 °C), we were interested in the catalytic performance of the mixed **RuH**‐**14** ISOMET dual catalyst system under ethylene pressure. Pretreated 96.5 % pure methyl oleate (**23**) was selected as the model compound for the preliminary investigation of the ISOMET reactions. The ISOMET of **23** was investigated using metathesis (**HG2**, **1**–**3**, and **14**) and isomerization (**RuH**) catalysts (Scheme 7). It was found that with catalyst **HG2** at 75 °C and 10 bar ethylene pressure in the presence of 1 mol % of metathesis and 2 mol % of isomerization catalysts only a moderate amount of ethenolysis (C10) products formation was produced (Table 1, entry 1). Propylene formation was not detected.

**Scheme 7:**
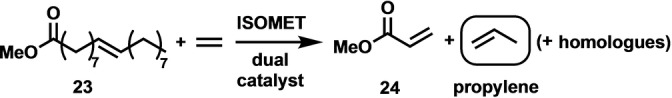
ISOMET of methyl oleate (**23**), purity 96.5 %; 75 °C; toluene; 24 h; 2 mol % **RuH**; 1 mol % **HG2**/**1**–**3/14**; [**23**]=0.25 M; *p*_Et(3.0)_=10 bar, ethylene purity 99.9 %.

**Table 1:** ISOMET of methyl oleate (**23**) using catalysts **HG2**, **3**, and **14**.^[a]^

Entry^[b]^	Catalyst	MO conv. [%]	TON^[e]^	Moles of propylene produced per mole of **23** ^[c]^
1	**HG2**	71	0	<1
2	**1**	86	3	<1
3	**2**	84	97	<1
4	**3**	85	11	<1
5^[d]^	**14**	92	1400	14

[a] Reaction conditions given in Scheme 7. [b] Reactions were reproduced. [c] The yield was calculated based on the propylene content of the gas phase using a gas chromatograph fitted with an FID detector. [d] Flushing the gas atmosphere after 24 h resulted in the formation of further propylene and lower molecular weight homologues (48 h); the experiment was reproduced. [e] Moles of propylene produced per mol of catalysts.

Using CAAC‐5 catalysts systems **1**–**3**, minimal propylene (less than one propylene per **23**) formation was observed. In contrast, with BICAAC−Ru catalyst **14**, almost complete conversion of **23**, high propylene yield, and some homologue formation was observed. It could be concluded that the BICAAC−Ru complex **14** shows not only latent properties but also significantly higher selectivity to propylene even in the presence of ethylene at elevated reaction temperatures in comparison to catalysts **HG2** and **1**–**3**. Using catalyst **14**, 14 mol of propylene could be produced from 1 mol of **24**, which equals 89 % propylene yield and 1400 TON (Table 1). A reaction carried out at higher (30 bar) ethylene pressure resulted in a lower propylene yield.

ISOMET of 1‐octadecene (**25**): Following the investigation of the ISOMET of **23**, our attention turned to the ISOMET of long‐chain olefins, the main component of HDPE mild pyrolysis oils.[^46^, ^47^, ^48^] 1‐Octadecene (**25**)—as one of the longest linear chain olefins in pyrolysis oil—was selected as model compound. The applied reaction conditions and the results of catalyst screening are given in Scheme 8 and Table 2, respectively. Catalysts **HG2**, **1**, and **2** showed significantly lower ISOMET activity, just as in case of **23**. However, catalyst **3** indicated significantly better performance (9300 TON). In addition, up to 21 000 TON could be obtained using high‐purity 99.995 % ethylene. Interestingly, the diisopropylphenyl (dipp) derivative **5** exhibited limited activity; only a minor amount of propylene formation was observed (Table 2), while mesityl derivative **18** (Scheme 5) showed a TON similar to that of complex **14** (23 000 vs. 26 000). The self‐metathesis of 1‐decene at 10 ppm loading showed a similar trend: Minimal activity was found for **5** (TON: 130), whereas catalyst **14** provided high product yield (TON: 54 000) within three hours reaction time. It is worth mentioning that the olefin metathesis activity of complexes **18**, **19**, and **5** display a declining trend (Table 2), which may be related to the increasing bulkiness of the alkyl groups in the *ortho* positions. *Bis*‐BICAAC complexes **16** and **20** also displayed ISOMET catalytic activity; however, the observed TONs were nearly half of that of catalyst **14**. The dimethylamino *bis*‐BICAAC ruthenium complex (**16**) gave slightly higher TON than its mesityl (**20**) analogue (Table 2).

**Scheme 8:**
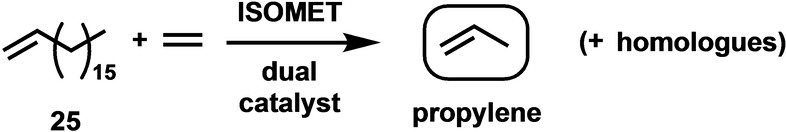
ISOMET of 1‐octadecene (**25**). 75 °C; toluene; [**25**]=1.95 M; *p*_Et_=10 bar; 24 h; [**RuH**]=200 ppm; ethylene purity 99.9 %.

**Table 2:** ISOMET of 1‐octadecene (**25**) with different catalysts.^[a]^

Entry	Catalyst	[Catalyst] in ppm	TON^[b]^
1	–	0	0
2	**HG2**	10	4700^[c]^
3	**1**	10	1700^[d]^
4	**2**	10	4800^[e]^
5	**3**	10	9300
6	**5**	10	<40^[f]^
7	**14**	10	26 000
8	**16**	10	16 000
9	**18**	10	23 000
10	**19**	10	11 000
11	**20**	10	12 000

[a] Reaction conditions are given in Scheme 8. [b] Moles of propylene produced per mol of catalyst. [c] Average of three runs, SDEV: 688. [d] Ethylene purity 99.995 %. [e] Average of three runs, SDEV: 285. [f] Average of two runs.

The ISOMET of **25** at different isomerization catalyst (**RuH**) loadings revealed that at higher concentration (from 20 to 2000 ppm) significantly higher propylene yield could be obtained (Table 3). It should be noted, however, that even in the absence of an isomerization catalyst, some propylene formation was also observed for catalysts **1**–**3** and **14**. This can be explained by the intrinsic decomposition of the metathesis catalysts via β‐elimination, leading to Ru−H species inducing double‐bond isomerization.[^24^, ^25^, ^26^] The positive effect of increasing **RuH** loading on propylene yield showed that the “bottleneck” of propylene formation is certainly the rate of isomerization.

**Table 3:** The influence of **RuH** loading on the ISOMET of 1‐octadecene (**25**).^[a]^

Entry	Catalyst	[**RuH**] in ppm	TON^[b]^
1	**1**	0	1900
2	**2**	0	2400
3	**3**	0	600
4	**14**	0	2200
5	**14**	20	7600
6	**14**	200	26 000
7	**14**	2000	36 000

[a] 75 °C; toluene; [**26**]=1.95 M; [**Catalyst**]=10 ppm; *p*_Et_=10 bar; 24 h; ethylene purity 99.9 %. [b] Mole of of propylene produced per mol of catalyst.

The purity of the ethylene gas used strongly influenced the ISOMET activity of the catalysts. In fact, changing the ethylene gas from 99.9 % to 99.995 % purity almost doubled the propylene yield (Table 4). The catalyst systems still manifested some activity even after 48 hours (Table 5). The highest TON observed was 55 000 after 48 hours. Similar TONs could be achieved at as low as 1 ppm **14** loading. It should be noted, however, that more than half of the propylene formed within three hours at 10 ppm loading, indicating a high average turnover frequency (TOF=10 000 mol_propylene_ mol_catalyst_^−1^ h^−1^) in this reaction time frame.

**Table 4:** The influence of ethylene purity on the ISOMET of 1‐octadecene (**25**).^[a]^

Entry	Catalyst	Ethylene purity [%]	TON^[b]^
1	**14**	99.9	26 000
2	**14**	99.95	41 000^[c]^
3	**14**	99.995	51 000
4	**3**	99.995	21 000

[a] 75 °C; toluene; [**25**]=1.95 M; [**Catalyst**]=10 ppm; [**RuH**]=200 ppm; *p*_Et_=10 bar; 24 h. [b] Moles of propylene produced per mol of catalyst. [c] Average of three runs, SDEV: 910.

**Table 5:** The **i**nfluence of reaction time on the ISOMET of 1‐octadecene (**25**).^[a]^

Entry	*t* [h]	[Catalyst] in ppm	TON^[b]^
1	3	10	30 000
2	6	10	44 000
3	24	10	51 000
4	48	10	55 000
5	24	1	30 000
6	48	1	41 000
7	72	1	47 000
8	96	1	50 000

[a] 75 °C; toluene; [**25**]=1.95 M; [**RuH**]=200 ppm; *p*_Et_=10 bar; ethylene purity 99.995 %. [b] Moles of propylene produced per mol of catalyst **14**.

A new generation of BICAAC−Ru latent olefin metathesis catalysts has been developed, displaying exceptional ethenolysis activity at elevated reaction temperature. ISOMET reaction of 96.5 % pure methyl oleate (**23**) at 75 °C reaction temperature under 10 bar ethylene (99.9 % purity) pressure in the presence of 1 mol % of **14** metathesis and 2 mol % of **RuH** isomerization catalysts resulted in almost quantitative conversion of **23** to propylene (TON=1400). The ISOMET reactions of 1‐octadecene (**25**) indicated that long‐chain olefins—which are the main components of HDPE mild pyrolysis oil—can be decomposed selectively to propylene even at as low as 1 ppm BICAAC−Ru (**14**) catalyst loading at 75 °C reaction temperature using high‐grade ethylene (99.995 %) (TON=50 000). The highest TON (55 000) was attained at 10 ppm loading of catalyst **14** after 48 h reaction time. The bottleneck of the ISOMET reactions is presumably the rate of the isomerization reaction step.

## Conflict of interest

The authors declare no conflict of interest.

## Supporting information

As a service to our authors and readers, this journal provides supporting information supplied by the authors. Such materials are peer reviewed and may be re‐organized for online delivery, but are not copy‐edited or typeset. Technical support issues arising from supporting information (other than missing files) should be addressed to the authors.

Supporting Information

## Data Availability

The data that support the findings of this study are available in the Supporting Information of this article.
