# Supplementary material for: Catalytic Decomposition of Long‐Chain Olefins to Propylene via Isomerization‐Metathesis Using Latent Bicyclic (Alkyl)(Amino)Carbene‐Ruthenium Olefin Metathesis Catalysts
Source: Angew Chem Int Ed Engl. 2022 May 19;61(28):e202204413. doi: 10.1002/anie.202204413 (PMC9400880; doi:10.1002/anie.202204413)
Supplement: Supplementary file 1 — Supporting Information [file ANIE-61-0-s001.pdf]

Angewandte Chemie
Eine Zeitschrift der Gesellschaft Deutscher Chemiker

Supporting Information

Catalytic Decomposition of Long-Chain Olefins to Propylene via Isomerization-Metathesis Using Latent Bicyclic (Alkyl)(Amino)Carbene-Ruthenium Olefin Metathesis Catalysts

*M. Nagyházi, Á. Lukács, G. Turczel, J. Hancsók, J. Valyon, A. Bényei, S. Kéki, R. Tuba**

Table of Contents

Table of Contents 2
1. General information 3
2. Catalyst synthesis 4
2.1. Synthesis of BICAAC carbene precursors **10**-**13** 4
2.1.1. Synthesis of imine **8c** 5
2.1.2. Synthesis of alkylated imine **9c** 6
2.1.3. Synthesis of BICAAC precursor **12** 7
2.2. Analytical data and NMR spectra of the respective iminium salts **10**-**13** 7
2.2.1. Compound **10** 7
2.2.2. Compound **11** 9
2.2.3. Compound **12** 11
2.2.4. Compound **13** 13
2.3. Synthesis of mono-BICAAC-Ru complexes **5**, **14**, **18** and **19** 15
2.3.1. Complex **14** 16
2.3.2. Complex **5** 18
2.3.3. Complex **18** 21
2.3.4. Complex **19** 23
2.4. Synthesis of bis-BICAAC-Ru complexes **16** and **20** 25
2.4.1. Complex **16** 26
2.4.2. Complex **20** 28
2.5. Synthesis of ionic mono-BICAAC-Ru complex **15** 31
2.6. Synthesis of ionic bis-BICAAC-Ru complex **17** 33
3. Catalytic experiments 34
3.1. Representative example of the RCM of diethyl diallylmalonate (**21**) in toluene-d_8 at 50 °C or 75 °C (0.05 mol% catalyst load) 34
3.2. Representative example of the latent properties in the RCM of diethyl diallylmalonate (**21**) in toluene-d_8 (0.25 mol% catalyst load) 36
3.3. RCM of diethyl diallylmalonate (**21**) with catalyst **5** in toluene-d_8 at 100 °C 36
3.4. Representative example of the ISOMET reaction of methyl oleate (**23**) 36
3.5. Representative example of the ISOMET reaction of 1-octadecene (**25**) 37
3.6. Representative example for the CM of 1-decene with catalyst **14** in neat at 75 °C 38
4. X-ray structure determination 39
5. References 44
Author Contributions 45

1. General information

All metathesis reactions were conducted under nitrogen atmosphere using Schlenk-technique or under argon using a glovebox. The reagents and solvents (Aldrich) including deuterated solvents (Eurisotop) were used as received. 1-Octadecene (Aldrich) was filtered through a short pad of activated alumina in the glovebox. Catalyst ultraNitroCat (**1**) and ultraCat (**3**) was acquired from Strem Chemicals.

High pressure reactions were carried out in a 150 mL Fischer-Porter bottle, using ethylene 99.9%, 99.95% or 99.995% (Linde).
NMR spectra were recorded on Varian Unity INOVA and Varian INOVA spectrometers operating at an equivalent ^{1}H frequency of 499.64 and 299.96 MHz respectively. Abbreviation used in the NMR assignments: Bzy: benzylidene, Ar: aromatic ring (*i. e. N*-aryl group), Cy: cyclohexene ring, BICAAC: 2-azabicyclo[2.2.2]octan ring. The abbreviations C-*Me* and CH-*Me* refer to methyl groups connecting to the 2-azabicyclo[2.2.2]octan ring.

GC-MS analyses were carried out using a Shimadzu GC-MS-QP2010 instrument fitted with an Rxi-5Sil MS column coupled with a quadrupole mass filter with pre-rods. The gaseous reaction products were analyzed on-line by a Shimadzu GC-2010 gas chromatograph (GC) equipped with a 50-m HP-PLOT-Fused Silica column (Al_2O_3, KCl), flame ionization detector (FID).

For the high resolution mass spectrometric measurements (HRMS) a Maxis II type Qq-TOF MS instrument (Bruker Daltonics, Bremen, Germany) with an electrospray ion-source were used. The spray voltage was kept at 3.5 kV and N_2 was used as the drying (200 °C, 4.0 L/min) and nebulizer gas (0.5 bar). The mass spectra were recorded by a digitizer at a sampling rate of 2 GHz. The mass accuracy of the instrument is better than 600 ppb (internal calibration) and the resolution power is higher than 40000 at m/z 400 (fwhm). The MS spectra were calibrated internally with sodium formate clusters formed *in-situ* under electrospray and evaluated by means of the Compass DataAnalysis 4.4 software from Bruker Daltonics (Bremen, Germany). The samples were dissolved in dichloromethane (DCM) and then diluted with methanol (MeOH) (MeOH/DCM : 9/1 V/V) to obtain sample concentrations of 0.01-0.04 mg/mL.

X-ray-quality crystals of **5, 14, 18** and **20** were grown by slow evaporation or cooling of hexane or DCM solutions. A crystal well-looking in polarized light microscope was fixed under a microscope onto a Mitegen loop using high-density oil. Diffraction intensity data were collected at room temperature (295-300 K) using a Bruker-D8 Venture diffractometer (Bruker AXS GmbH, Karlsruhe, Germany) equipped with INCOATEC IμS 3.0 (Incoatec GmbH, Geesthacht, Germany) dual (Cu and Mo) sealed tube micro sources and a Photon II Charge-Integrating Pixel Array detector (Bruker AXS GmbH, Karlsruhe, Germany) using Mo Kα (λ = 0.71073 Å) radiation, in case of **5** Cu Kα. radiation was applied because of the rather long unit cell axis. High multiplicity data collection and integration were performed using APEX3 (version 2017.3-0, Bruker AXS Inc., 2017, Madison, USA) software. Data reduction and multi-scan absorption correction were performed using SAINT (version 8.38A, Bruker AXS Inc., 2017, Madison, USA). The structure was solved using direct methods and refined on F^2 using the SHELXL program[1] incorporated into the APEX3 suite. Refinement was performed anisotropically for all non-hydrogen atoms. Hydrogen atoms were placed into geometric positions The CIF file was manually edited using Publcif software,[2] while graphics were prepared using the Mercury program.[3] The results for the X-ray diffraction structure determinations were very good according to the Checkcif functionality of PLATON software (Utrecht University, Utrecht, The Netherlands),[4] and structural parameters such as bond length and angle data were in the expected range. Deposition Numbers 2128047-2128050 for **14**, **18**, **5** and **20** (respectively), as well as 2144205 and 2144206 for the other enantiomer of **18** and the inversion twin crystal of **18** from the racemic conglomerate and 2144599 for **15**, respectively contain the supplementary crystallographic data for this paper. These data are provided free of charge by the joint Cambridge Crystallographic Data Centre and Fachinformationszentrum Karlsruhe Access Structures service.

2. Catalyst synthesis

*2.1. Synthesis of BICAAC carbene precursors **10**-**13***

Following the synthetic procedure of **13** (^{Me}BICAAC^{dipp}) reported by the Bertrand group,[5] two recently published by our group[6] (**10**, ^{Me}BICAAC^{NMe2}; **11**, ^{Me}BICAAC^{mes}) and one new (**12**, ^{Me}BICAAC^{Et2}) BICAAC-HBF_4 precursor salts have been prepared.

The synthetic procedure reported by the Bertrand group was slightly modified. At step (**i**), molecular sieves were used instead of magnesium sulfate as drying agent. Molecular sieves have greater drying intensity than the magnesium sulfate and its acidity replaces the *para*-toluenesulfonic acid catalyst. At step (**ii**), lithium diisopropylamide (LDA) solution was used instead of butyllithium solution. The basicity of the LDA is also sufficient for the deprotonation of the imine, and does not require intensive cooling (0 °C is enough instead of -78 °C). At step (**iii**), 1,4-dioxane was used as solvent instead of diethyl ether. This change was applied for only safety reasons. The indicated alternative for generating hydrogen chloride *in-situ* by reacting dry methanol with acetyl chloride afforded the products (**10-13**) in same yields as in case of commercially available dioxane-HCl solutions.

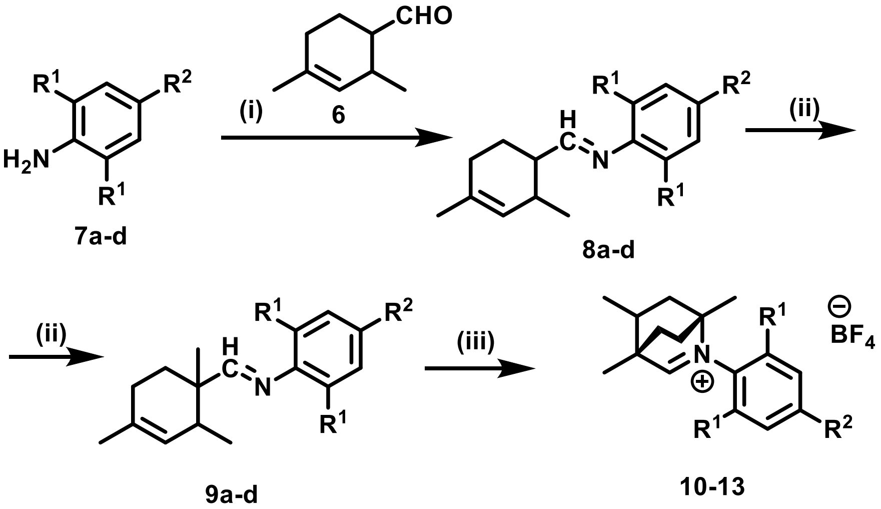

a: R^1 = Me, R^2 = NMe_2 — **10: ^{Me}BiCAAC^{NMe2}**
b: R^1 = Me, R^2 = Me — **11: ^{Me}BiCAAC^{mes}**
c: R^1 = Et, R^2 = H — **12: ^{Me}BiCAAC^{Et2}**
d: R^1 = iPr, R^2 = H — **13: ^{Me}BiCAAC^{dipp}**

Synthesis of carbene precursors **10-13**

The aldehyde (**6,** Trivertal, Fluorochem) and anilines **7b-d** (Merck) used in reaction step **i** are commercially available. Compound **7a** was synthetized following the procedure described in a paper published by our group recently.[7] As a representative example, herein the synthesis of the novel ^{Me}BICAAC^{Et2} ligand's precursor (**12**) is reported in detail.

*2.1.1. Synthesis of imine **8c***

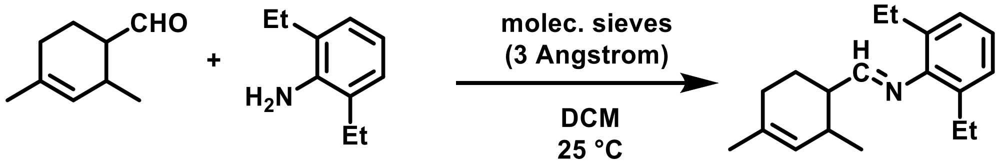

6 **7c** **8c**

Synthesis of imine **8c**

2,4-Dimethylcyclohex-3-en-1-carbaldehyde (Trivertal, **6**, 33.83 mmol, 5.00 mL) and aniline **7c** (5.57 mL, 33.83 mmol), were mixed in a Schlenk-flask in equimolar ratio, then the flask was evacuated and filled with dry nitrogen. To the cloudy emulsion, 40 mL of dry dichloromethane and 20 g of dry molecular sieves (3 Å) were added. The formed solution was left overnight (16 hours) at room temperature without stirring, but shaked gently, occasionally. The resulted, clear, pale yellow solution was analyzed by GC-MS and NMR. After approx. 90% conversion, the mixture was filtered and concentrated. The crude product was dried on high vacuum using a 90 °C oil bath to evaporate the unreacted starting materials. In the ^{1}H NMR, two diastereomers were observed, as the imine and the 2-Me group may be positioned either *cis* or *trans* positions to each other. The product (**8c**) is a yellow oil (8.20 g, 90%), stored under nitrogen in the dark.

N-(2,6-Diethylphenyl)-1-(2,4-dimethylcyclohex-3-en-1-yl)methanimine (**8c**): yellow oil, (8.20 g, yield: 90%). In ^{1}H NMR, two diastereomers were observed in a 80-20% molar ratio.

^{1}H NMR (300 MHz, $CDCl_3$) δ: 7.69 (d, *J* = 5.5 Hz, 0.2 × 1H, C*H*=N, minor isomer), 7.60 (d, *J* = 5.5 Hz, 1H, 0.8 × 1H, C*H*=N, major isomer), 7.05 (d, *J* = 7.5 Hz, 2H, C*H*$_{Ar}$), 7.02 – 6.97 (m, 1H, C*H*$_{Ar}$), 5.38 (s, 0.2 × 1H, olefinic C=C*H*, minor isomer), 5.30 (s, 0.8 × 1H, olefinic C=C*H*, major isomer), 2.81 – 2.74 (m, 0.2 × 1H, C*H*$_{Cy}$, minor isomer), 2.65 – 2.56 (m, 0.2 × 1H, C*H*$_{Cy}$, minor isomer), 2.52 – 2.41 (m, 4H, *CH*$_2$Et and undef. amount of C*H*$_{Cy}$), 2.31 – 2.22 (m, 0.8 × 1H, C*H*$_{Cy}$, major isomer), 2.14 – 1.95 (m, 3H, C*H*$_{Cy}$), 1.79 – 1.72 (m, 1H, C*H*$_{Cy}$), 1.71 (s, 3H, C-*Me*), 1.19 – 1.10 (m, 9H, *Me*$_{Et}$ and the major isomer of CH-*Me*), 1.06 (d, *J* = 7.2 Hz, 0.2 × 2H, CH-*Me*, minor isomer).

^{13}C NMR (75 MHz, $CDCl_3$) both isomers δ: 170.91, 170.19, 150.47, 133.29, 133.14, 133.09, 126.54, 126.52, 126.31, 126.23, 123.76, 123.72, 48.03, 44.51, 32.78, 32.19, 29.19, 28.57, 25.88, 24.71, 24.65, 23.77, 23.65, 23.59, 20.85, 18.08, 14.90, 14.79.

HRMS: calculated m/z: 270.2216, found: 270.2215 (for $[M+H]^+$: $C_{19}H_{28}N^+$).

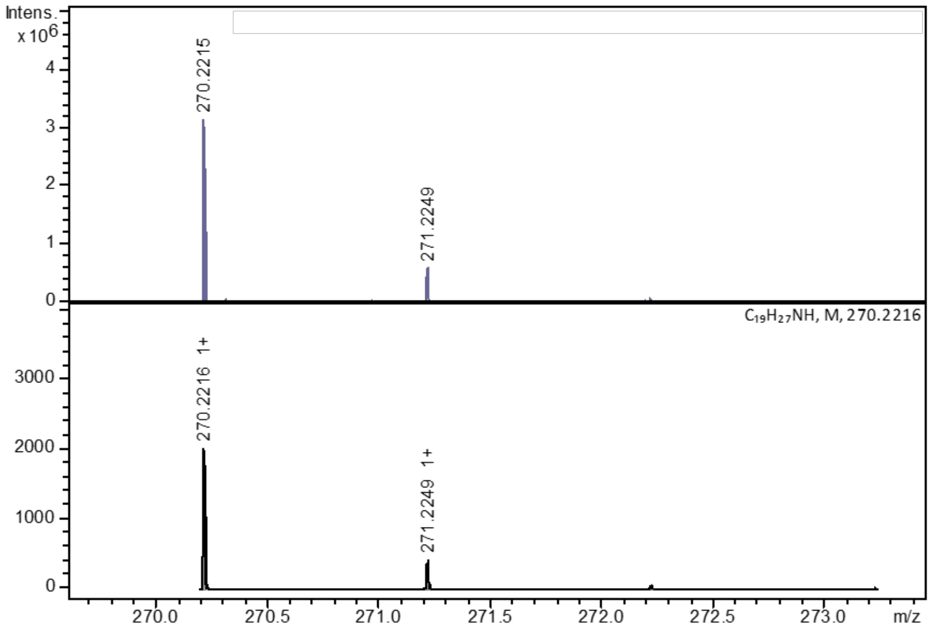

Fig. S1. ESI-MS mass spectrum of compound **8c.** Measured (top) and calculated (bottom) masses and isotopic distributions.

2.1.2. Synthesis of alkylated imine **9c**

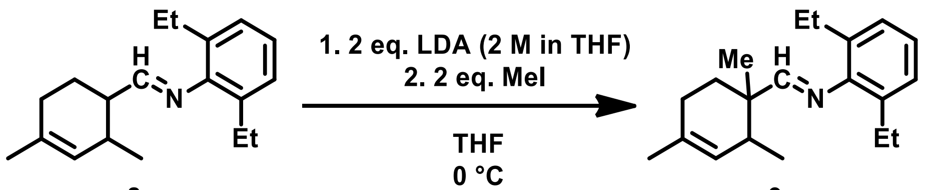

Synthesis of imine **9c**

In a Schlenk-flask, imine **8c** (8.08 g, 30.00 mmol) was dissolved in dry THF (20 mL). Lithium-diisopropylamide (LDA) solution (2 M in THF, 60.00 mmol, 30 mL) was diluted with another crop of dry THF (20 mL), than it was cooled to 0 °C using an ice-bath. To this stirred LDA solution, the imine's solutin was added dropwise, then the resulted reddish-brown mixture was allowed to warm up to room temperature. Then the mixture was stirred for additional 4 hours. (Note: less than 4 hours of deprotonation gradually decreases the reaction's yield.) The solution was cooled down to 0 °C, and methyl iodide (3.73 mL, 60.00 mmol) was added dropwise resulting a yellow suspension. The mixture was stirred overnight (16 h), then the reaction's progress was checked with GC-MS. Then distilled water and hexane were added to the suspension, until the precipitated inorganic salt dissolved. The organic layer was washed three times with water (40 mL each) and once with diluted (10%) sodium bisulfite solution (40 mL). Finally, it was dried over anhydrous sodium sulfate, filtered and the solution concentrated at reduced pressure. As commercially available LDA-solutions contain ethylbenzene as co-solvent, the product was dried on high vacuum using a 90 °C oil bath. The product (**9c**) is a yellow oil (8.076 g, 95%), stored under nitrogen in the dark.

N-(2,6-Diethylphenyl)-1-(1,2,4-trimethylcyclohex-3-en-1-yl)methanimine (**9c**): yellow oil (8.076 g, yield: 95%). In ^{1}H NMR, two diastereomers were observed in a 87-13% molar ratio.

^{1}H NMR (300 MHz, $CDCl_3$) δ: 7.69 (s, 0.87 × 1H, C*H*=N, major isomer), 7.53 (s, 0.13 × 1H, C*H*=N, minor isomer), 7.22 – 6.94 (m, 3H, CH_{Ar}), 5.30 (s, 0.13 × 1H, olefinic C=C*H*, minor isomer), 5.27 (s, 0.87 × 1H, olefinic C=C*H*, major isomer), 2.45 (q, *J* = 7.5 Hz, 4H, CH_{2Et}), 2.31 – 2.18 (m, 1H, C$H_{aliphatic}$), 2.11 – 1.96 (m, 2H, C$H_{aliphatic}$), 1.80 – 1.69 (m, 2H, C$H_{aliphatic}$), 1.67 (s, 3H, C-*Me*), 1.28 (s, 3H, C-*Me*), 1.15 (t, J = 7.5 Hz, 6H, Me_{Et}), 1.06 (d, *J* = 7.3 Hz, 0.87 × 3H, CH-*Me*, major isomer), 1.01 (d, *J* = 7.2 Hz, 0.13 × 1H, CH-*Me*, minor isomer).

^{13}C NMR (75 MHz, $CDCl_3$) major isomer only δ: 172.00, 150.75, 133.44, 132.96, 126.20, 123.56, 41.86, 39.09, 32.60, 28.10, 24.64, 23.76, 23.34, 17.19, 14.79.

HRMS: calculated m/z: 284.2373, found: 284.2371 (for $[M+H]^+$: $C_{20}H_{30}N^+$).

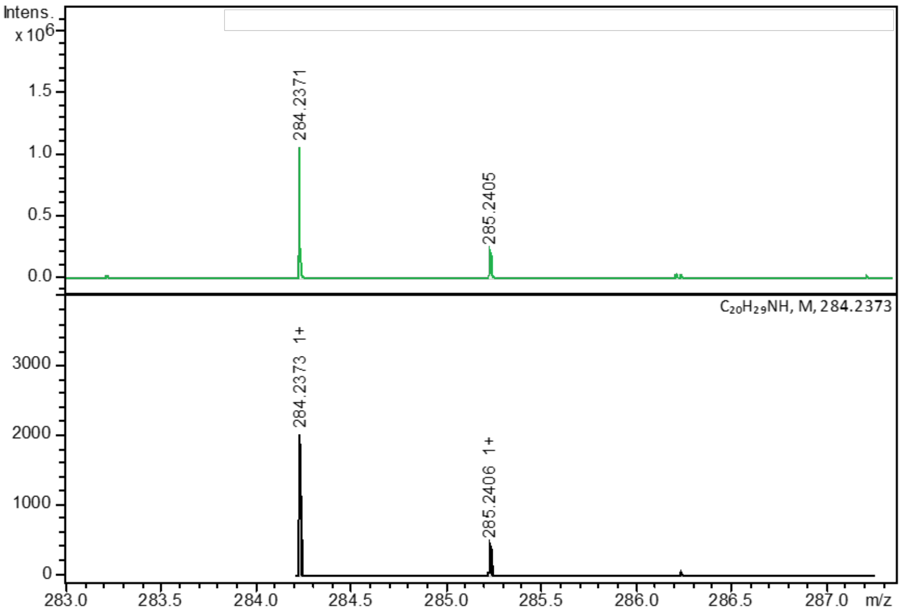

Fig. S2. ESI-MS mass spectrum of compound **9c**. Measured (top) and calculated (bottom) masses and isotopic distributions.

2.1.3. Synthesis of BICAAC precursor **12**

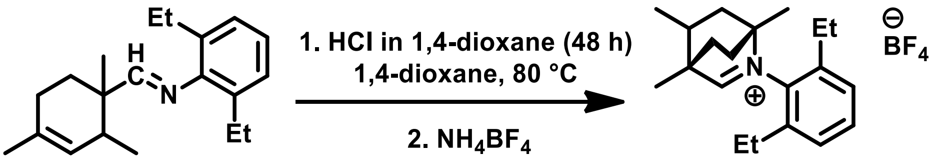

9c **12**

Synthesis of BICAAC precursor salt **12**

In a Schlenk-flask, imine **9c** (7.086 g, 25.00 mmol) was dissolved in dry 1,4-dioxane (15 mL), followed by the addition of 1,4-dioxane solution of 3 molar hydrochloric acid (41.66 mL, 125.00 mmol) at room temperature. Alternatively, the HCl can be generated *in-situ* by reacting dry methanol with acetyl chloride in equimolar ratio in dry dioxane. The flask was then sealed and stirred at 80 °C for at least 48 hours. The formed solution or suspension was allowed to cool down to room temperature, then concentrated sodium bicarbonate solution (50 mL) and dichloromethane (80 mL) were added to neutralize the hydrochloric acid. The organic phase was separated, concentrated at reduced pressure, and then aqueous solution of ammonium tetrafluoroborate (50.00 mmol, 5.24 g, cca. 50 mL solution) was added to the dichloromethane solution. The resulted mixture was stirred for two hours, then separated, and the organic phase was evaporated affording the mixture of the unreacted starting material (**9c**) and the product. Adding diethyl ether to this mixture dissolved the impurities and induced the crystallization of the product. The crude BICAAC salt can be recrystallized from hot hexane, giving **12** as a pale-yellow powder.

2.2. Analytical data and NMR spectra of the respective iminium salts **10-13**

Each iminium compound were synthetized following the procedure described above.

2.2.1. Compound **10**

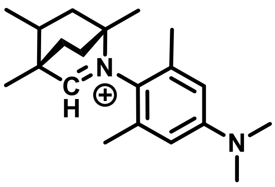

10

2-(2,6-Dimethyl-4-dimethylaminophenyl)-1,4,5-trimethyl-2-azabicyclo[2.2.2]oct-2-en-2-ium tetrafluoroborate (^{Me}BICAAC^{NMe2}-HBF$_4$, **10**): light brown solid, yield: 14%. Note that this compound's ring closing reaction requires doubled amount of hydrochloric acid due to the *tertiary* amine group.

^{1}H NMR (500 MHz, CDCl$_3$) δ: 9.06 (s, 1H, C*H*=N$^+$), 6.37 (s, 2H, Ar$_{CH}$), 2.96 (s, 6H, N*Me$_2$*), 2.44 (dd, *J* = 14.0, 10.1 Hz, 1H, C*H*$_{BICAAC}$), 2.30 – 2.21 (m, C*H*$_{BICAAC}$), 2.20 (s, 3H, Ar-*Me*), 2.14 (s, 3H, Ar-*Me*), 2.12 – 2.01 (m, 2H, C*H*$_{BICAAC}$), 2.00 – 1.91 (m, 1H, C*H*$_{BICAAC}$), 1.89 – 1.81 (m, 1H, C*H*$_{BICAAC}$), 1.63 (s, 3H, C-*Me*), 1.62 – 1.56 (m, 1H, C*H*$_{BICAAC}$), 1.24 (s, 3H, C-*Me*), 1.07 (d, *J* = 7.2 Hz, 3H, CH-*Me*).

^{13}C NMR (75 MHz, CDCl$_3$) δ: 193.76, 150.85, 133.57, 132.88, 127.92, 112.03, 111.95, 69.37, 44.92, 43.82, 40.13, 38.46, 33.32, 33.17, 21.42, 20.61, 20.56, 19.21, 18.80.

^{19}F NMR (282 MHz, CDCl$_3$) δ: -152.32 ($^{11}BF_4^-$), -152.37 ($^{10}BF_4^-$).

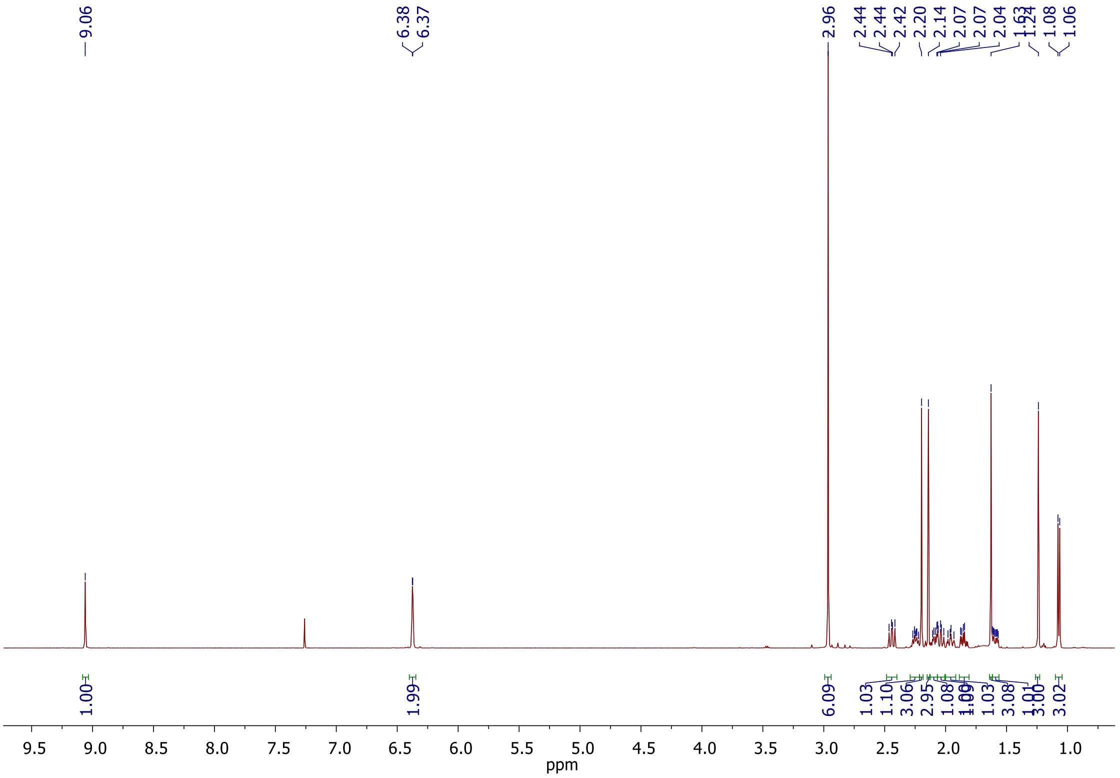

Fig. S3. ^{1}H NMR spectra of compound **10**. Solvent: $CDCl_3$

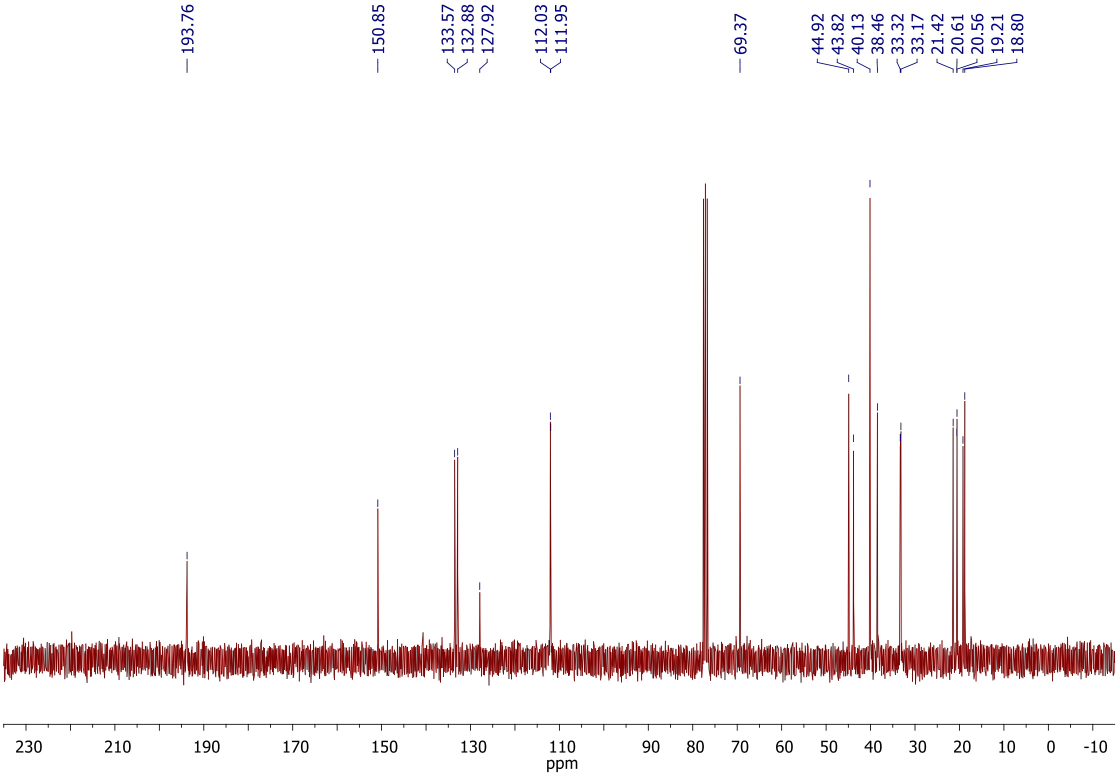

Fig. S4. ^{13}C NMR spectra of compound **10**. Solvent: $CDCl_3$

HRMS: calculated m/z: 299.248175, recorded: 299.2481 (for organic cation $C_{20}H_{31}N_2^+$).

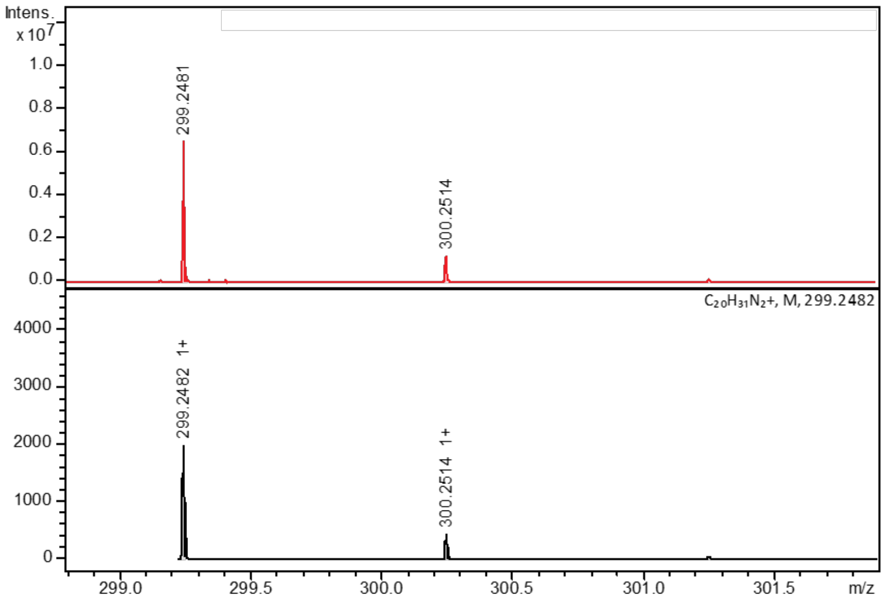

Fig. S5. ESI-MS mass spectrum of compound **10** along with the measured (upper) and calculated (lower) masses and isotopic distributions

*2.2.2. Compound **11***

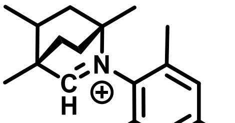

11

2-(2,4,6-Trimethylphenyl)-1,4,5-trimethyl-2-azabicyclo[2.2.2]oct-2-en-2-ium tetrafluoroborate (^{Me}BICAAC^{NMe2}-HBF$_4$, **11**): off-white solid, yield: 13%.

^{1}H NMR (500 MHz, $CDCl_3$) δ: 9.12 (s, 1H, C<u>*H*</u>=N$^+$), 6.98 (s, 2H, C<u>*H*</u>$_{Ar}$), 2.48 (dd, *J* = 14.0, 10.1 Hz, 1H, C<u>*H*</u>$_{BICAAC}$), 2.29 (s, 3H, Ar-<u>*Me*</u>), 2.28 – 2.23 (m, 1H, C<u>*H*</u>$_{BICAAC}$), 2.22 (s, 3H, Ar-<u>*Me*</u>), 2.16 (s, 3H, Ar-<u>*Me*</u>), 2.14 – 2.10 (m, 1H, C<u>*H*</u>$_{BICAAC}$), 2.10 – 2.03 (m, 1H, C<u>*H*</u>$_{BICAAC}$), 2.01 – 1.93 (m, 1H, C<u>*H*</u>$_{BICAAC}$), 1.84 (td, *J* = 12.2, 5.4 Hz, 1H, C<u>*H*</u>$_{BICAAC}$), 1.62 (s, 3H, C-<u>*Me*</u>), 1.61 – 1.58 (m, 1H, C<u>*H*</u>$_{BICAAC}$), 1.23 (s, 3H, C-<u>*Me*</u>), 1.07 (d, *J* = 7.2 Hz, 3H, CH-<u>*Me*</u>).

^{13}C NMR (75 MHz, $CDCl_3$) δ: 194.29, 141.13, 136.49, 132.98, 132.29, 130.73, 130.56, 69.24, 45.05, 44.05, 38.57, 33.46, 33.25, 21.35, 21.00, 20.50, 20.02, 18.76, 18.65.

^{19}F NMR (282 MHz, $CDCl_3$) δ: -152.20 ($^{11}BF_4^-$), -152.25 ($^{10}BF_4^-$).

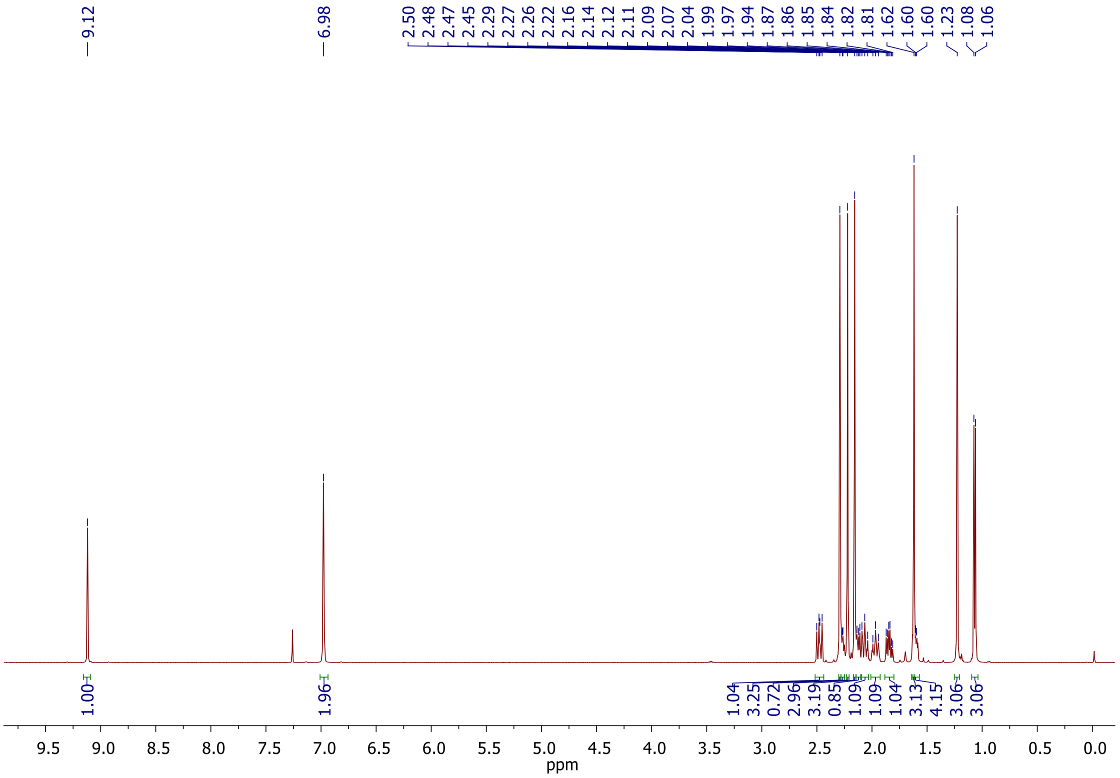

Fig. S6. ^{1}H NMR spectra of compound **11**. Solvent: $CDCl_3$

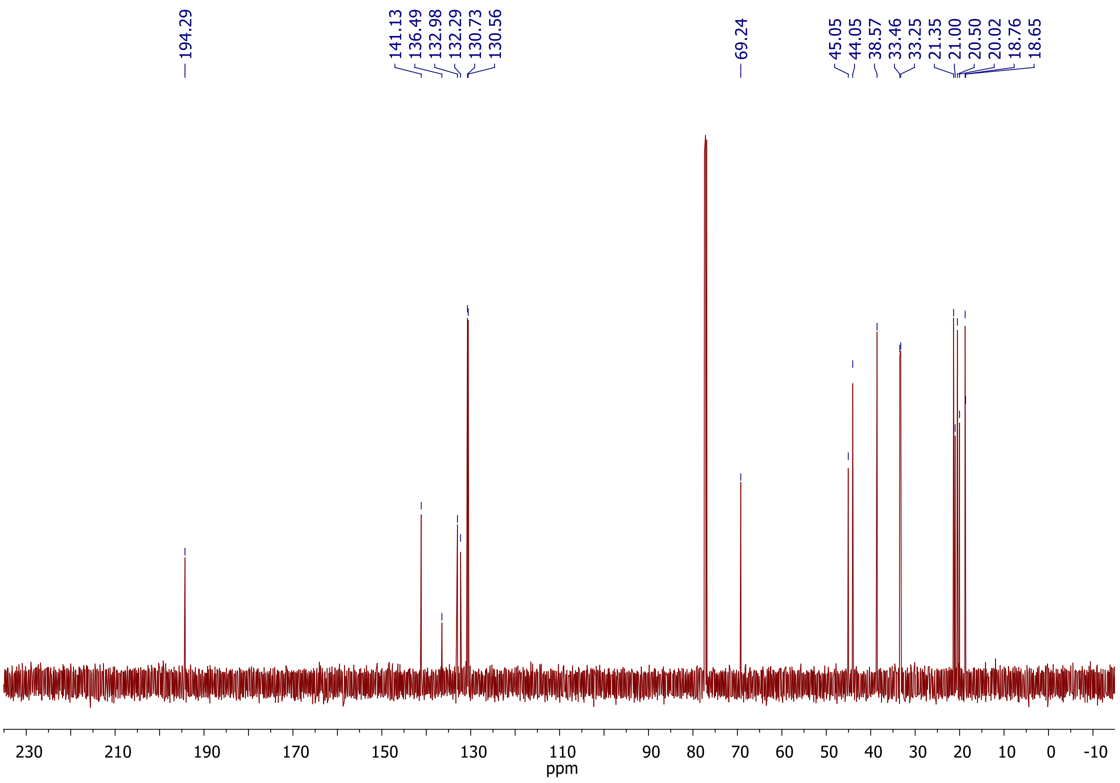

Fig. S7. ^{13}C NMR spectra of compound **11**. Solvent: $CDCl_3$

HRMS: calculated m/z: 270.221626, recorded: 270.2213 (for organic cation $C_{19}H_{28}N^+$).

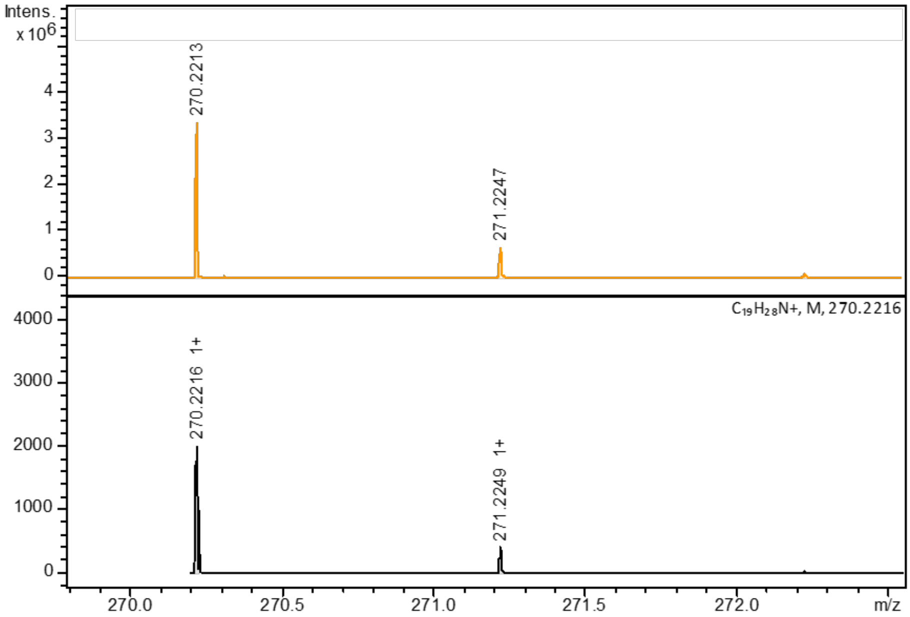

Fig. S8. ESI-MS mass spectrum of compound **11** along with the measured (upper) and calculated (lower) masses and isotopic distribution

*2.2.3. Compound **12***

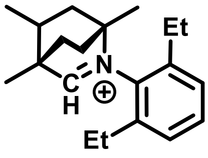

12

2-(2,6-Diethylphenyl)-1,4,5-trimethyl-2-azabicyclo[2.2.2]oct-2-en-2-ium tetrafluoroborate (^{Me}BICAAC^{Et2}-HBF$_4$, **12**): brown solid, 0.185 g, yield: 5%.

^{1}H NMR (500 MHz, CDCl$_3$) δ: 9.23 (s, 1H, C<u>H</u>=N), 7.46 (t, *J* = 7.8 Hz, 1H, C<u>H</u>$_{Ar}$), 7.29 (dd, *J* = 12.0, 7.7 Hz, 2H, C<u>H</u>$_{Ar}$), 2.60 – 2.45 (m, 4H, C<u>H$_2$</u>$_{Et}$), 2.37 – 2.26 (m, 2H, C<u>H</u>$_{BICAAC}$), 2.19 – 2.07 (m, 2H, C<u>H</u>$_{BICAAC}$), 2.05 – 1.98 (m, 1H, C<u>H</u>$_{BICAAC}$), 1.96 – 1.87 (m, 1H, C<u>H</u>$_{BICAAC}$), 1.72 – 1.65 (m, 1H, C<u>H</u>$_{BICAAC}$), 1.67 (s, 3H, C-<u>*Me*</u>), 1.28 (td, *J* = 7.5, 5.2 Hz, 6H, <u>*Me*</u>$_{Et}$), 1.22 (s, 3H, C-<u>*Me*</u>), 1.10 (d, *J* = 7.2 Hz, 3H, CH-<u>*Me*</u>).

^{13}C NMR (75 MHz, CDCl$_3$) δ: 194.28, 138.80, 138.21, 137.42, 131.28, 127.57, 127.44, 69.37, 45.20, 43.88, 38.56, 33.33, 33.27, 25.24, 24.03, 21.43, 20.46, 18.85, 14.48, 14.31.

^{19}F NMR (282 MHz, CDCl$_3$) δ: -152.37 ($^{11}BF_4^-$), -152.42 ($^{10}BF_4^-$).

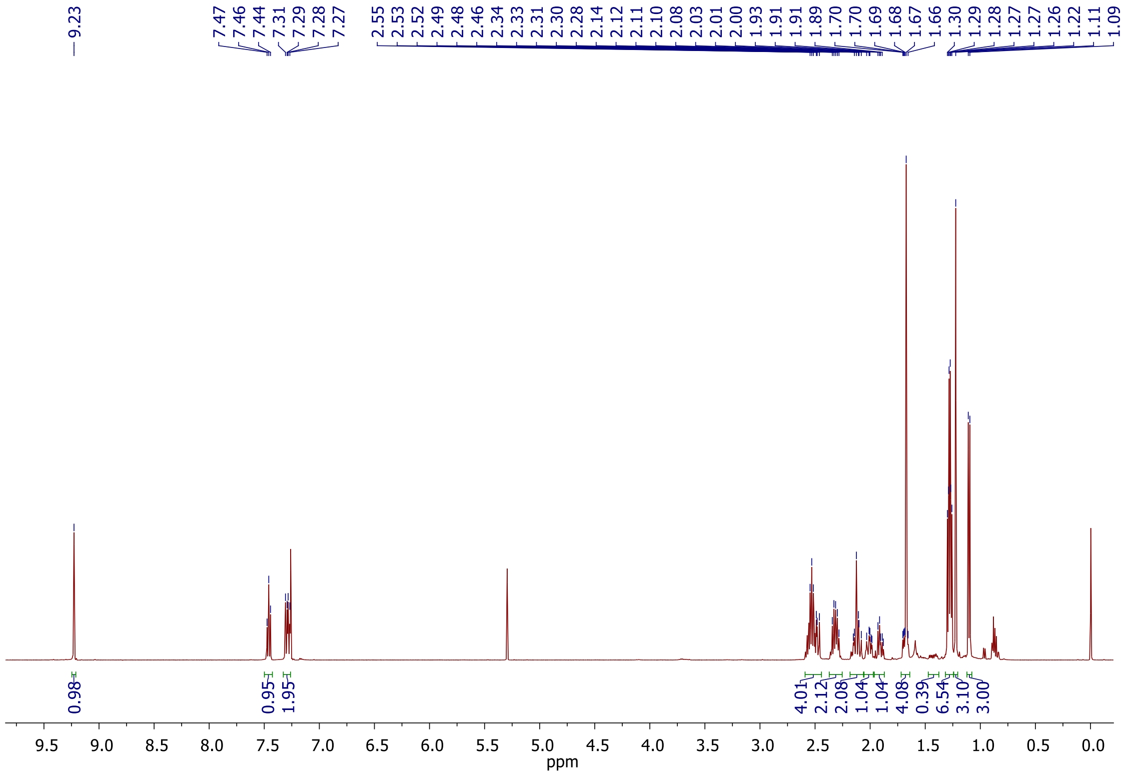

Fig. S9. ^{1}H NMR spectra of compound **12**. Solvent: $CDCl_3$

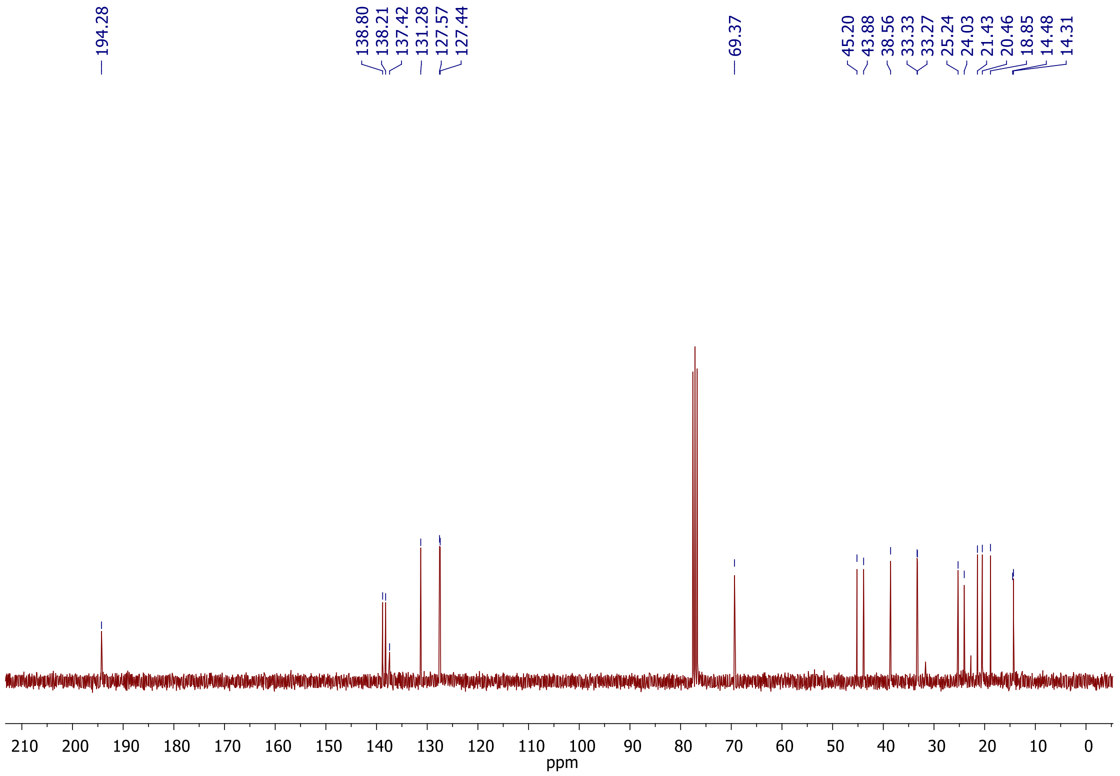

Fig. S10. ^{13}C NMR spectra of compound **12**. Solvent: $CDCl_3$

HRMS: calculated m/z: 284.2373, found: 284.2371 (for M^+: $C_{20}H_{30}N^+$).

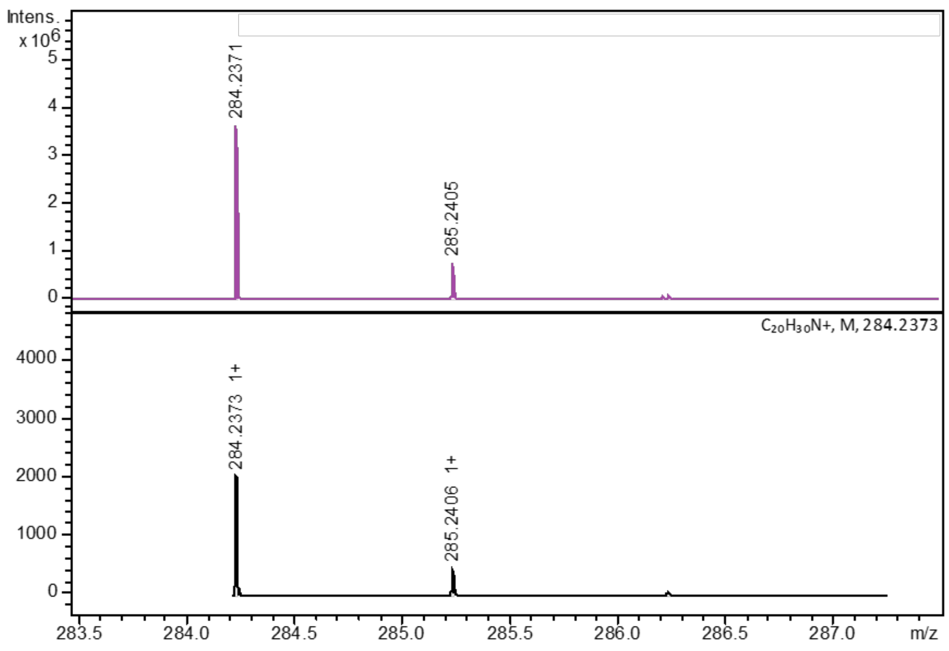

Fig. S11. ESI-MS mass spectrum of compound **12**. Measured (top) and calculated (bottom) masses and isotopic distributions.

*2.2.4. Compound **13***

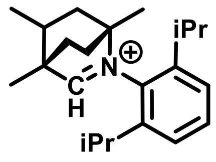

13

2-(2,6-Diisopropylphenyl)-1,4,5-trimethyl-2-azabicyclo[2.2.2]oct-2-en-2-ium tetrafluoroborate (^{Me}BICAAC^{dipp}-HBF_4, **13**): white solid, yield: 15%.

^{1}H NMR (300 MHz, $CDCl_3$) δ: 9.24 (s, 1H, C<u>*H*</u>=N), 7.49 (t, *J* = 7.8 Hz, 1H, C$\underline{H}_{Ar}$), 7.30 (d, *J* = 8.0 Hz, 2H, C$\underline{H}_{Ar}$), 2.72 – 2.51 (m, 2H, C$\underline{H}_{BICAAC}$), 2.41 – 2.26 (m, 2H, C$\underline{H}_{iPr\text{-}Ar}$), 2.26 – 2.09 (m, 2H, C$\underline{H}_{BICAAC}$), 1.88 (t, J = 12.5 Hz, 1H, C$\underline{H}_{BICAAC}$), 1.80 – 1.67 (m, 1H, C$\underline{H}_{BICAAC}$), 1.62 (s, 3H, C-$\underline{Me}_{BICAAC}$), 1.61 – 1.55 (m, 1H, C$\underline{H}_{BICAAC}$), 1.32 (d, *J* = 6.8 Hz, 3H, $\underline{Me}_{iPr}$), 1.26 (d, *J* = 6.8 Hz, 3H, $\underline{Me}_{iPr}$), 1.20 (d, *J* = 6.9 Hz, 3H, $\underline{Me}_{iPr}$), 1.18 (s, 3H, C-$\underline{Me}_{BICAAC}$), 1.15 (d, *J* = 6.8 Hz, 3H, $\underline{Me}_{iPr}$), 1.03 (d, *J* = 7.2 Hz, 3H, CH-$\underline{Me}_{BICAAC}$).

^{13}C NMR (75 MHz, $CDCl_3$) δ: 194.13, 143.84, 143.34, 135.36, 131.79, 125.72, 125.34, 69.64, 45.51, 43.55, 38.65, 33.18, 33.00, 29.83, 29.16, 25.06, 24.97, 23.79, 22.77, 21.83, 20.54, 19.07.

^{19}F NMR (282 MHz, $CDCl_3$) δ: -151.90 ($^{11}BF_4^-$), -151.96 ($^{10}BF_4^-$).

NMR data are the same as previously published.[5]

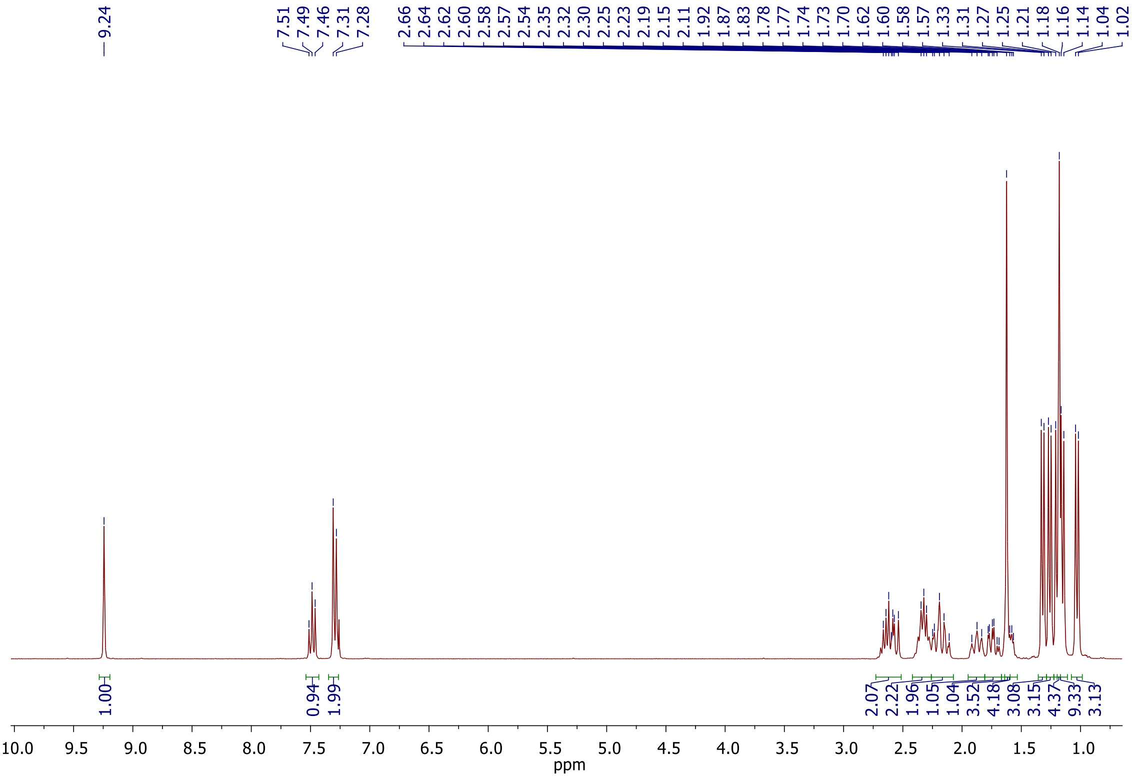

Fig. S12. ^{1}H NMR spectra of compound **13**. Solvent: $CDCl_3$

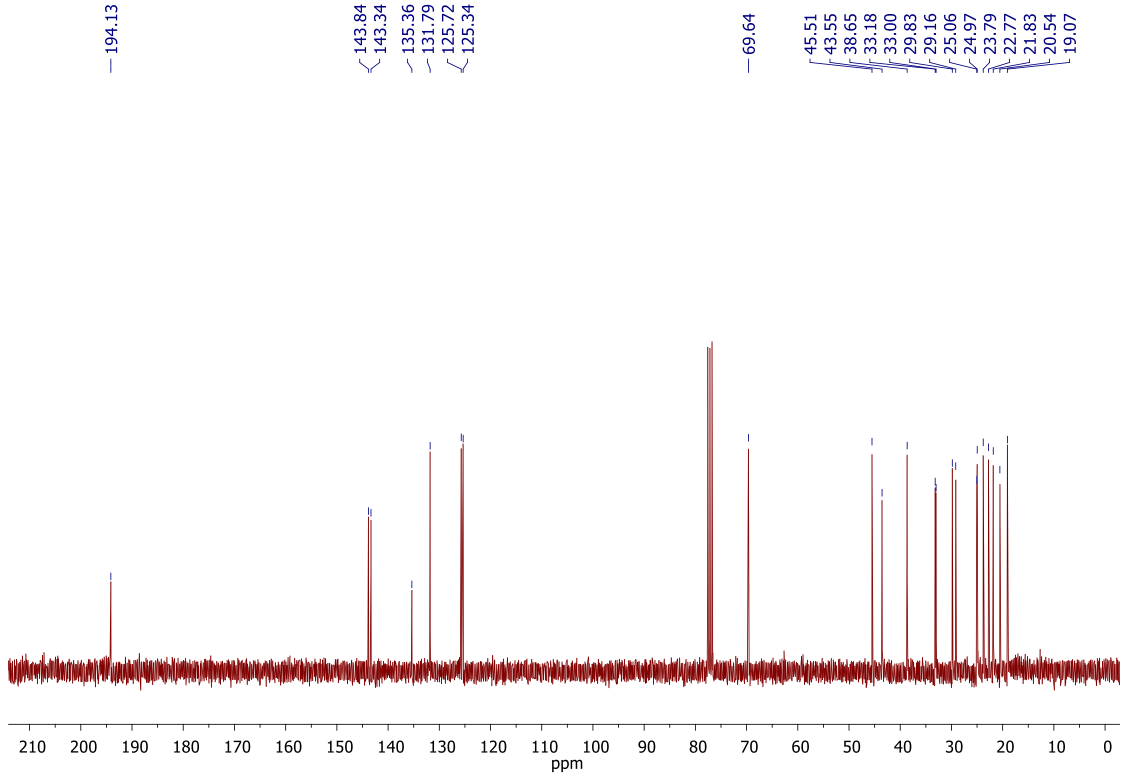

Fig. S13. ^{13}C NMR spectra of compound **13**. Solvent: $CDCl_3$

*2.3. Synthesis of mono-BICAAC-Ru complexes **5**, **14**, **18** and **19***

For complexations, the free carbenes were not isolated. Their *in-situ* preparation from the corresponding precursor salts and their subsequent complexation was a straightforward method. This method was already used for the synthesis of CAAC-5 systems.[7,8]

General procedure for *mono*-BICAAC-Ru complexes

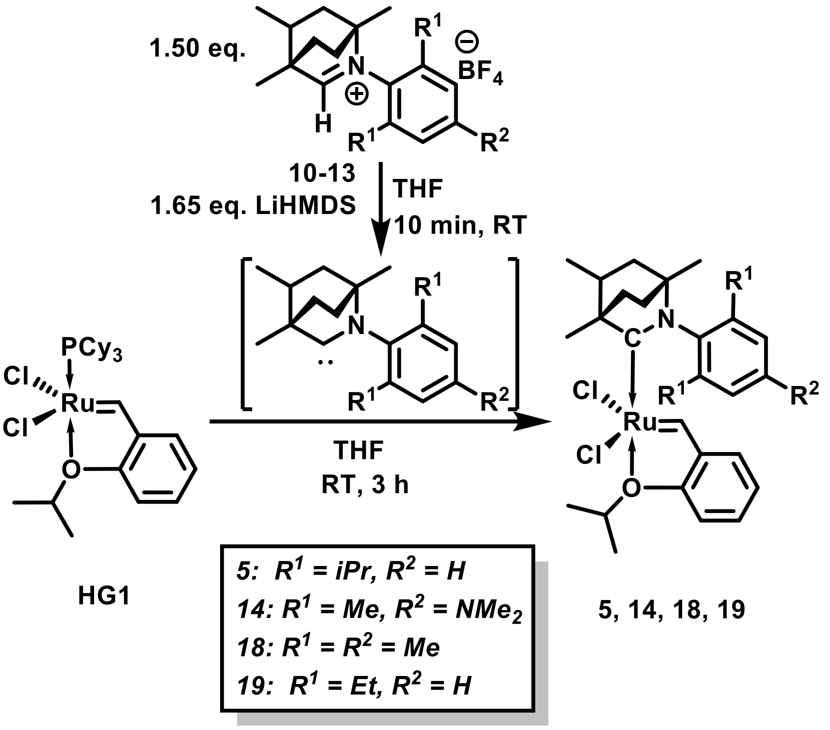

Synthesis of *mono*-carbene complexes **5**, **14**, **18** and **19**

In glovebox, Hoveyda-Grubbs first generation complex (**HG1**, 167 μmol, 100.0 mg) and the HBF_4-salt of the BICAAC precursor (250 μmol, 96.5 mg **10**, 88.8 mg **11**, 92.7 mg **12**, 99.7 mg **13**) were measured into two different vials. Dry THF (5-5 mL) was added to both compounds forming a suspension (**10-13**) and a solution (**HG1**). The LiHMDS (1 M in THF, 275 μmol, 275 μL) was added to the stirred suspension of the BICAAC precursors at room temperature, resulting a clear solution immediately, indicating the formation of the free carbene. Depending on the substrate, it may be colorless (**13**), yellow (**11**) or brown (**10** and **12**). This solution was added to the stirred solution of the **HG1** complex yielding a brownish solution. The conversion of the reaction can be followed by ^{1}H and ^{31}P NMR, and also visually, as the reaction is progressing the color of the mixture is gradually turning green. The reaction time is minimum 20 minutes (*e.g.* **13**), but no more than 3 hours.

The resulted solution was filtered through an alumina pad and concentrated at reduced pressure. Depending on the crystallization ability of the complex, it can be purified by crystallization (**14** and **5**) or column chromatography (**18** and **19**). For crystallization, the crude material was dissolved in warm (40-50 °C) hexane (cca. 10 mL) to form a concentrated solution, then it was cooled to -20 °C (freezer). Green crystals were formed overnight. For the chromatography, the crude material was taken up in hexane under air, then it was layered on the top of a column. Hexane containing 0–50 vol.% ethyl acetate was used as eluent over alumina as stationary phase. The green fractions were collected and concentrated, affording the products as green solids upon evaporation of the solvent. The complexes are soluble in almost all organic solvents.

*2.3.1. Complex **14***

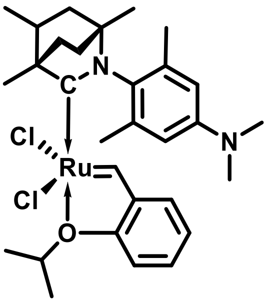

14

{2-[2,6-Dimethyl-4-dimethylaminophenyl]-1,4,5-trimethyl-2-azabicyclo[2.2.2]octan-3-ylidene}{2-isopropoxy-benzylidene}ruthenium(II) dichloride (^{Me}BICAAC^{NMe2}-HG complex, **14**): green solid, (64.9 mg, yield: 63%).

^{1}H NMR (500 MHz, $CDCl_3$) δ: 16.47 (s, 1H, Ru=C<u>*H*</u>), 7.54 (td, *J* = 8.4, 1.7 Hz, 1H, C<u>*H*</u>$_{Bzy}$), 7.00 – 6.85 (m, 3H, C<u>*H*</u>$_{Bzy}$), 6.59 (dd, *J* = 13.6, 2.7 Hz, 2H, C<u>*H*</u>$_{Ar}$), 5.16 (sept, *J* = 6.1 Hz, 1H, C<u>*H*</u>$_{iPrO}$), 3.06 (s, 6H, N<u>*Me$_2$*</u>), 2.82 (s, 3H, C-<u>*Me*</u>$_{BICAAC}$), 2.43 (dd, *J* = 11.2, 5.1 Hz, 1H, C<u>*H$_2$*</u>$_{BICAAC}$), 2.27 (s, 3H, <u>*Me*</u>-Ar), 2.17 (s, 3H, <u>*Me*</u>-Ar), 2.12 (dd, *J* = 13.0, 10.5 Hz, 1H, C<u>*H$_2$*</u>$_{BICAAC}$), 2.04 – 1.90 (m, 2H, C<u>*H*</u>$_{BICAAC}$ and 1H of C<u>*H$_2$*</u>$_{BICAAC}$), 1.75 (d, *J* = 6.3 Hz, 3H, <u>*Me*</u>$_{iPrO}$), 1.74 (d, *J* = 6.3 Hz, 3H, <u>*Me*</u>$_{iPrO}$), 1.73 – 1.64 (m, 2H, 1H of C<u>*H$_2$*</u>$_{BICAAC}$ and 1H of C<u>*H$_2$*</u>$_{BICAAC}$), 1.56 – 1.48 (m, 1H, C<u>*H$_2$*</u>$_{BICAAC}$), 1.32 (d, *J* = 7.1 Hz, 3H, CH-<u>*Me*</u>$_{BICAAC}$), 1.10 (s, 3H, C-<u>*Me*</u>$_{BICAAC}$).

^{13}C NMR (126 MHz, $CDCl_3$) δ: 306.40 (Ru=<u>C</u>H), 263.55 (BICAAC carbene), 151.92, 150.28, 144.99, 137.67, 137.63, 135.81, 130.81, 124.12, 122.10, 113.12, 113.11, 112.97, 74.70, 66.46, 54.07, 46.18, 40.80, 40.01, 33.90, 30.42, 23.96, 22.24, 22.14, 21.40, 21.28, 20.55, 19.80.

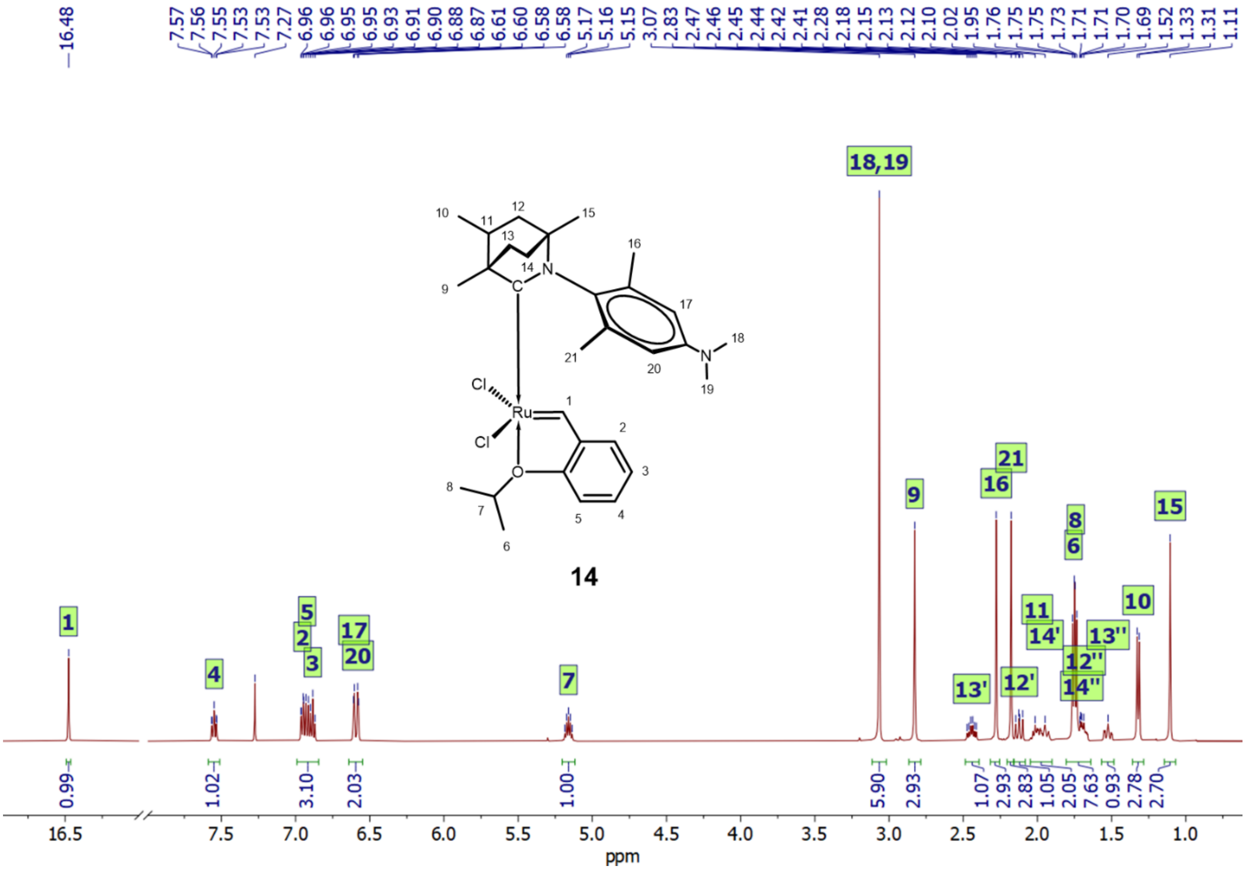

Fig. S14. ^{1}H NMR spectra of complex **14**, the shown assignation is based on NOESY and TOCSY measurements. Solvent: $CDCl_3$

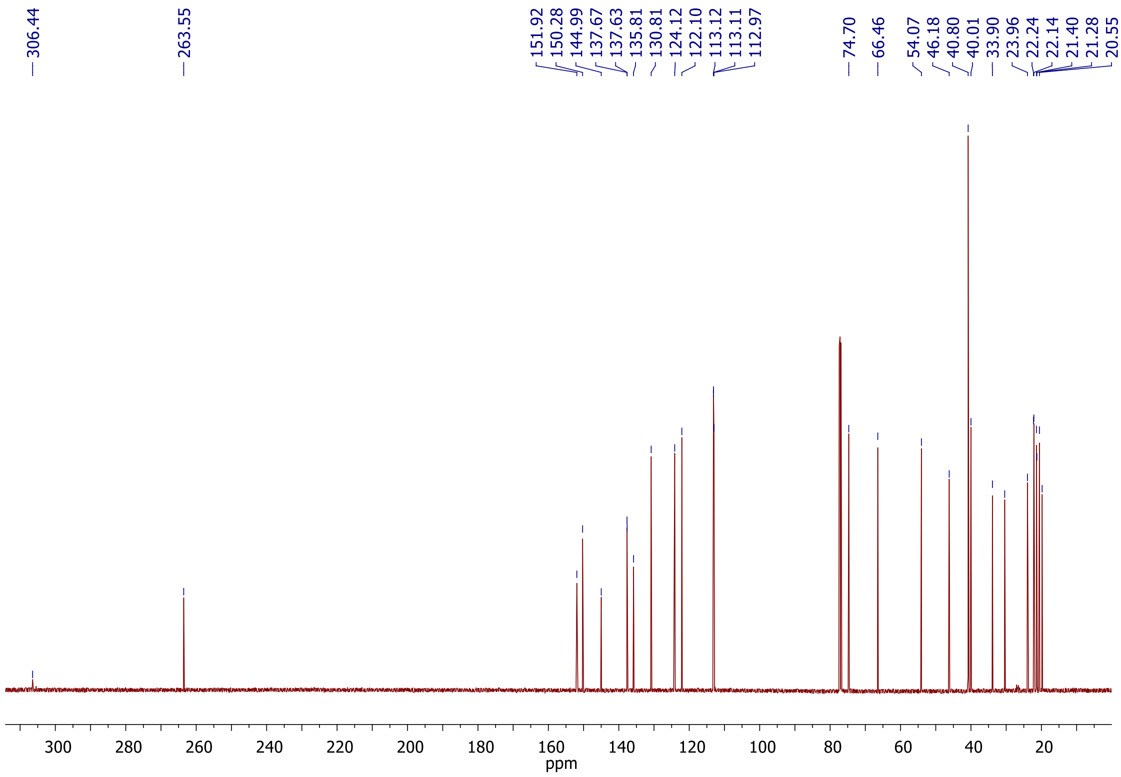

Fig. S15. ^{13}C NMR spectra of complex **14**. Solvent: $CDCl_3$

HRMS: calculated m/z: 583.2029, found: 583.2033 (for $[M-Cl]^+$:$C_{30}H_{42}N_2OClRu^+$).

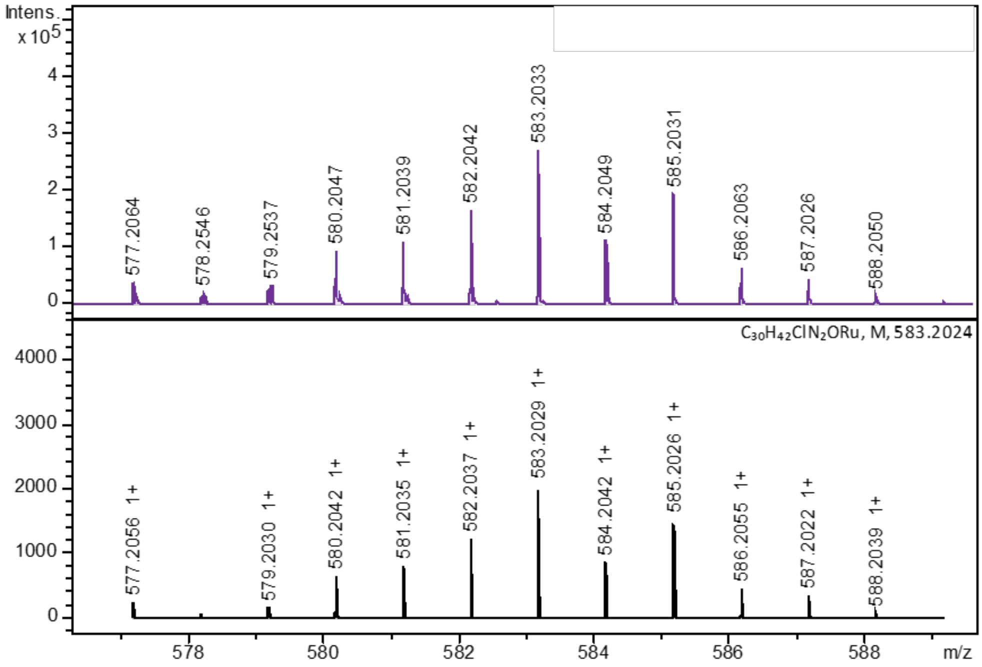

Fig. S16. ESI-MS mass spectrum of complex **14**. Measured (top) and calculated (bottom) masses and isotopic distributions.

*2.3.2. Complex **5***

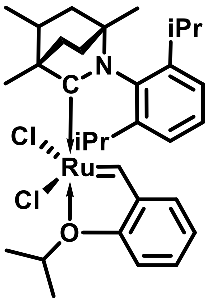

5

{2-[2,6-Diisopropylphenyl]-1,4,5-trimethyl-2-azabicyclo[2.2.2]octan-3-ylidene}{2-isopropoxy-benzylidene}ruthenium(II) dichloride (^{Me}BICAAC^{dipp}-HG complex, **5**): green solid, 83.3 mg, yield: 79%.

^{1}H NMR (500 MHz, $CDCl_3$) δ: 16.54 (s, 1H, Ru=C*H*), 7.61 (t, *J* = 7.7 Hz, 1H, C*H*$_{Ar}$), 7.51 (ddd, J = 8.5, 6.9, 2.2 Hz, 1H, C*H*$_{Bzy}$), 7.46 (dd, *J* = 7.8, 1.4 Hz, 1H, C*H*$_{Ar}$), 7.43 (dd, *J* = 7.7, 1.3 Hz, 1H, C*H*$_{Ar}$), 6.92 (d, *J* = 8.4 Hz, 1H, C*H*$_{Bzy}$), 6.86 – 6.78 (m, 2H, C*H*$_{Bzy}$), 5.14 (sept, *J* = 6.2 Hz, 1H, C*H*$_{iPrO}$), 3.11 (sept, *J* = 6.7 Hz, 1H, C*H*$_{iPr\text{-}Ar}$), 2.85 (sept, *J* = 6.7 Hz, 1H, C*H*$_{iPr\text{-}Ar}$), 2.85 (s, 3H, C-*Me*$_{BICAAC}$), 2.52 (td, *J* = 12.4, 6.1 Hz, 1H, C*H*$_{2BICAAC}$), 2.11 (dd, *J* = 13.1, 10.6 Hz, 1H, C*H*$_{2BICAAC}$), 2.07 – 1.98 (m, 1H, C*H*$_{BICAAC}$), 1.94 – 1.86 (m, 1H, C*H*$_{2BICAAC}$), 1.82 (d, J = 6.2 Hz, 3H, *Me*$_{iPrO}$), 1.76 (d, *J* = 6.1 Hz, 3H, *Me*$_{iPrO}$), 1.74 – 1.67 (m, 1H, C*H*$_{2BICAAC}$), 1.67 – 1.61 (m, 1H, C*H*$_{2BICAAC}$), 1.57 – 1.50 (m, 1H, C*H*$_{2BICAAC}$), 1.34 (d, *J* = 7.1 Hz, 3H, CH-*Me*$_{BICAAC}$), 1.29 (d, *J* = 7.2 Hz, 3H, *Me*$_{iPr\text{-}Ar}$), 1.27 (d, *J* = 7.1 Hz, 3H, *Me*$_{iPr\text{-}Ar}$), 0.97 (s, 3H, C-*Me*$_{BICAAC}$), 0.85 (d, *J* = 6.6 Hz, 3H, *Me*$_{iPr\text{-}Ar}$), 0.71 (d, *J* = 6.5 Hz, 3H, *Me*$_{iPr\text{-}Ar}$).

^{13}C NMR (126 MHz, $CDCl_3$) δ: 300.36 (Ru=*C*H), 264.04 (BICAAC carbene), 152.59, 147.11, 147.09, 143.92, 142.14, 130.84, 129.32, 125.48, 125.45, 123.97, 121.99, 113.29, 75.02, 66.76, 54.60, 45.06, 40.09, 33.02, 30.22, 30.17, 28.25, 28.05, 25.95, 25.81, 25.25, 25.12, 25.04, 24.94, 24.25, 22.60, 22.31, 22.23, 21.40, 21.28, 20.55.

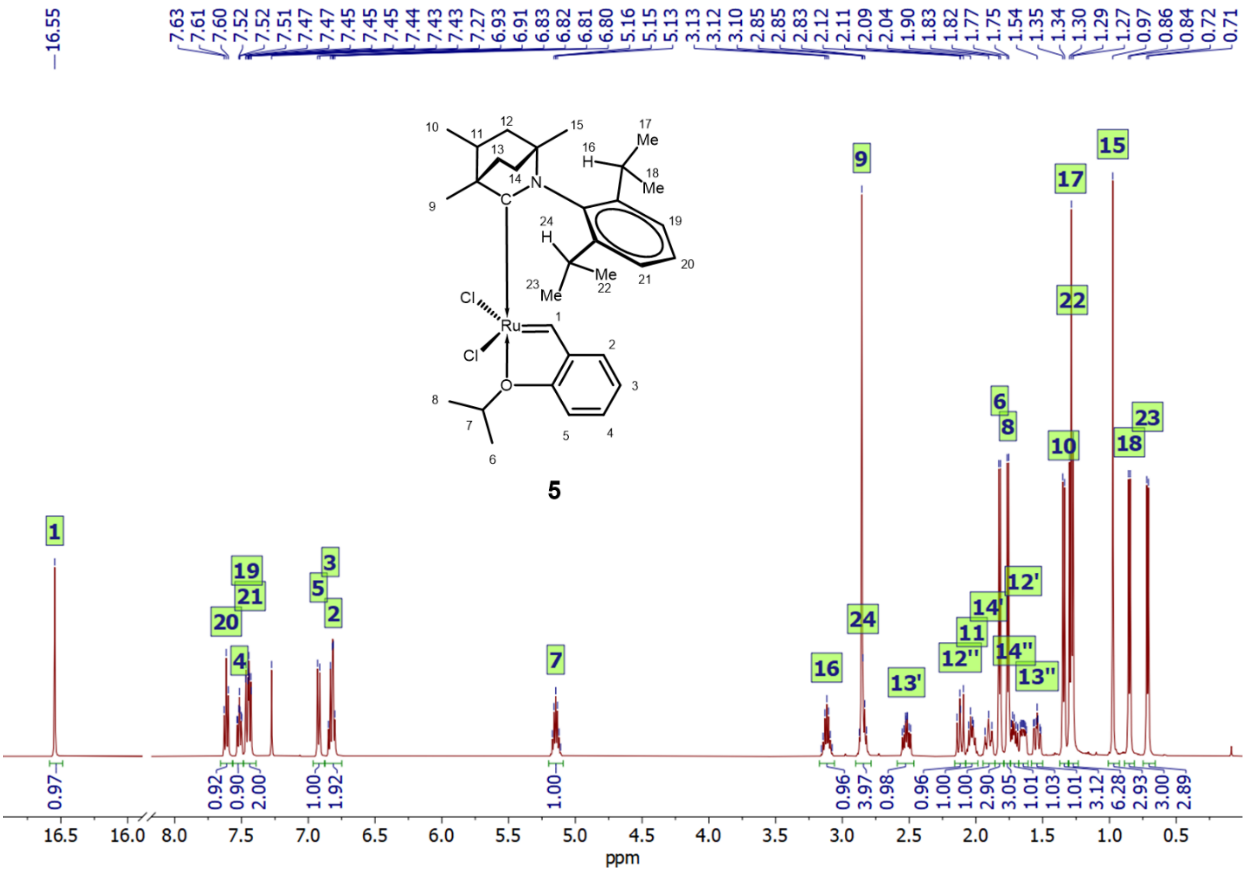

Fig. S17. ^{1}H NMR spectra of complex **5**, the shown assignation is based on NOESY measurements. Solvent: $CDCl_3$

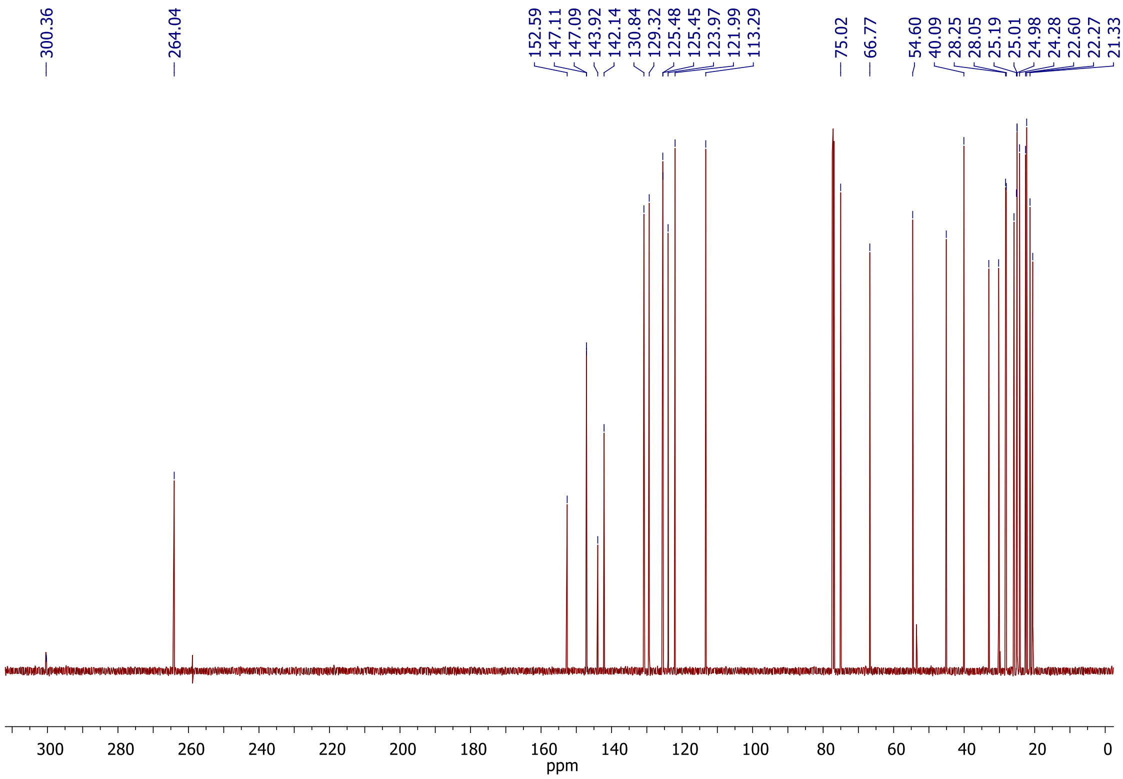

Fig. S18. ^{13}C NMR spectra of complex **5**. Solvent: $CDCl_3$

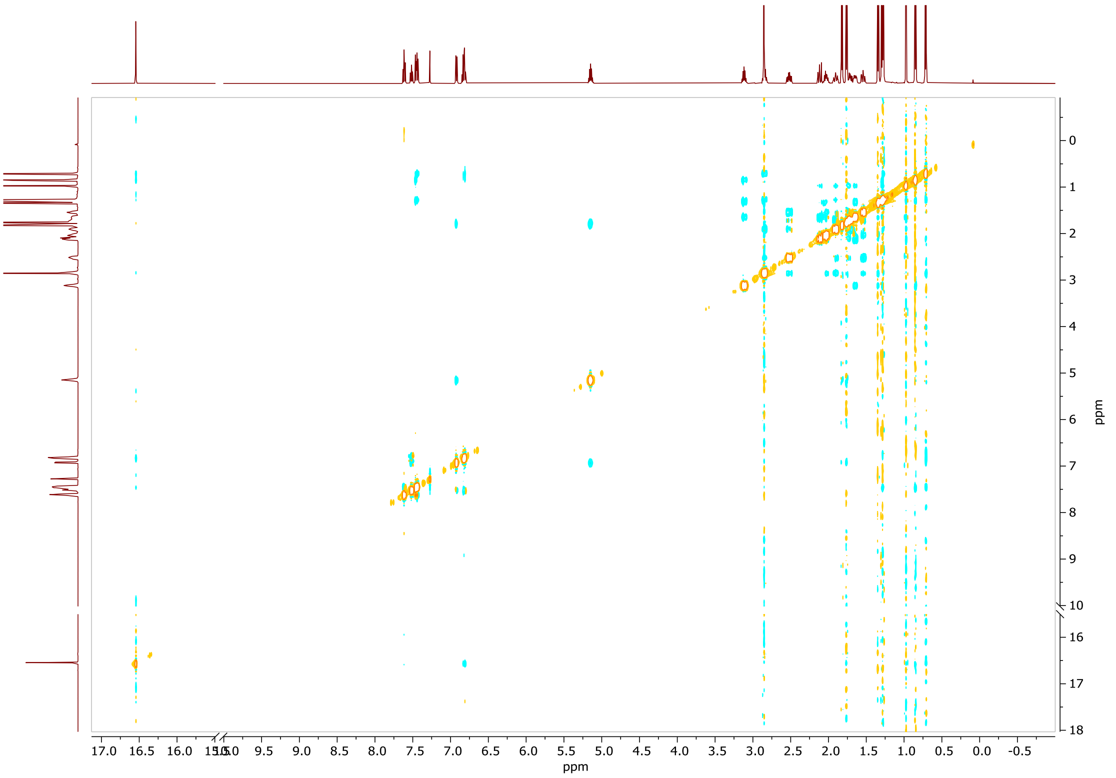

Fig. S19. 2D NOESY spectrum of complex **5**. Solvent: $CDCl_3$

HRMS: calculated m/z: 596.2233, found: 596.2232 (for [M-Cl]$^+$: $C_{32}H_{45}NOClRu^+$).

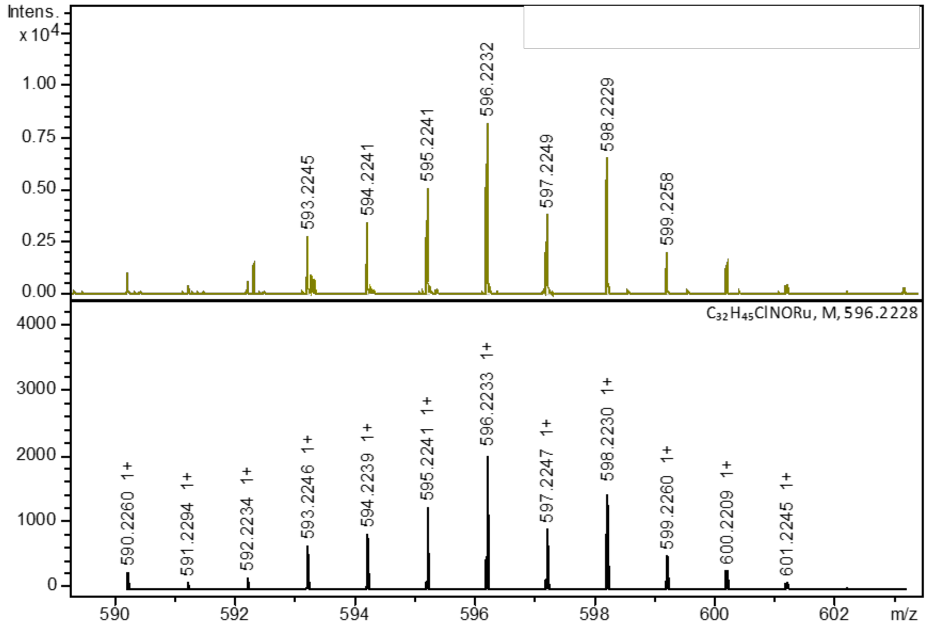

Fig. S20. ESI-MS mass spectrum of complex **5**. Measured (top) and calculated (bottom) masses and isotopic distributions.

*2.3.3. Complex **18***

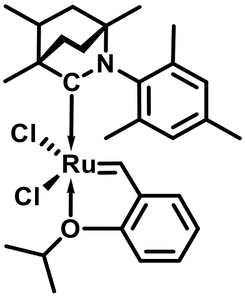

18

{2-[2,4,6-Trimethylphenyl]-1,4,5-trimethyl-2-azabicyclo[2.2.2]octan-3-ylidene}{2-isopropoxy-benzylidene}ruthenium(II) dichloride (^{Me}BICAAC^{mes}-HG complex, **18**): green solid, 75.8 mg, yield: 77%.

^{1}H NMR (500 MHz, $CDCl_3$) δ: 16.24 (s, 1H, Ru=C$\underline{H}$), 7.61 – 7.48 (m, 1H, C$\underline{H}_{Bzy}$), 7.08 (d, *J* = 6.2 Hz, 2H, C$\underline{H}_{Bzy}$), 6.96 – 6.82 (m, 3H, 1H C$\underline{H}_{Bzy}$ and 2H C$\underline{H}_{Ar}$), 5.15 (sept, *J* = 6.0 Hz, 1H, C$\underline{H}_{iPrO}$), 2.81 (s, 3H, C-Me$_{BICAAC}$), 2.47 (s, 3H, $\underline{Me}_{Ar}$), 2.46 – 2.37 (m, 1H, C$\underline{H}_{BICAAC}$), 2.28 (s, 3H, $\underline{Me}_{Ar}$), 2.18 (s, 3H, C-$\underline{Me}_{BICAAC}$), 2.10 (d, *J* = 12.3 Hz, 1H, C$\underline{H}_{BICAAC}$), 2.06 – 1.88 (m, 2H, C$\underline{H}_{BICAAC}$), 1.81 – 1.70 (m, 6H, 2 $\underline{Me}_{iPrO}$), 1.67 (s, 2H), 1.53 (t, *J* = 10.4 Hz, 1H), 1.31 (d, *J* = 6.8 Hz, 3H, CH-$\underline{Me}_{BICAAC}$), 1.07 (s, 3H, C-$\underline{Me}_{BICAAC}$).

^{13}C NMR (126 MHz, $CDCl_3$) δ: 305.44 (Ru=$\underline{C}$H), 263.64 (BICAAC carbene), 151.98, 144.94, 143.23, 138.36, 137.24, 137.21, 131.01, 130.36, 130.30, 124.18, 122.14, 113.15, 74.82, 66.37, 54.06, 46.27, 40.01, 33.99, 30.37, 23.87, 22.23, 22.14, 21.24, 21.15, 20.79, 19.93, 19.86.

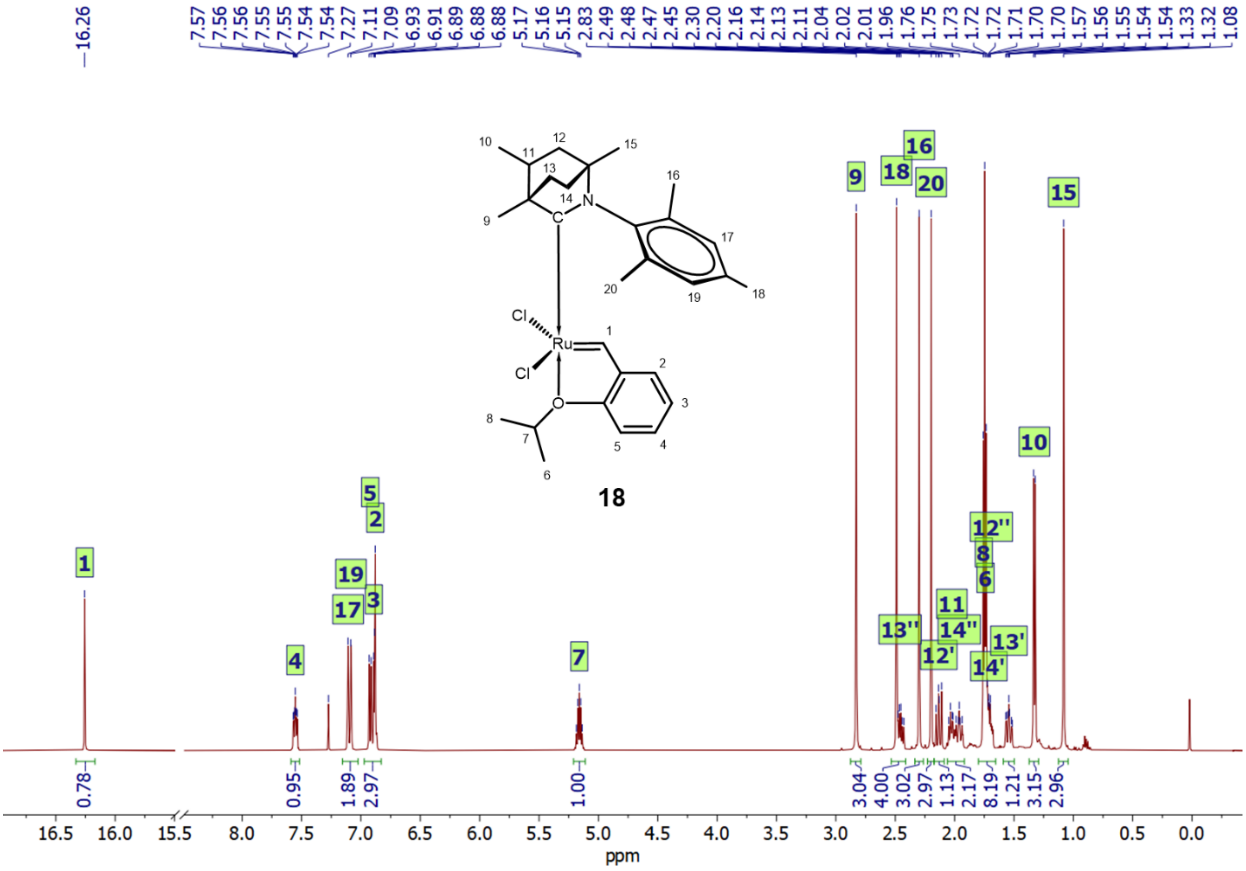

Fig. S21. ^{1}H NMR spectra of complex **18**, the shown assignation is based on NOESY and TOCSY measurements. Solvent: $CDCl_3$

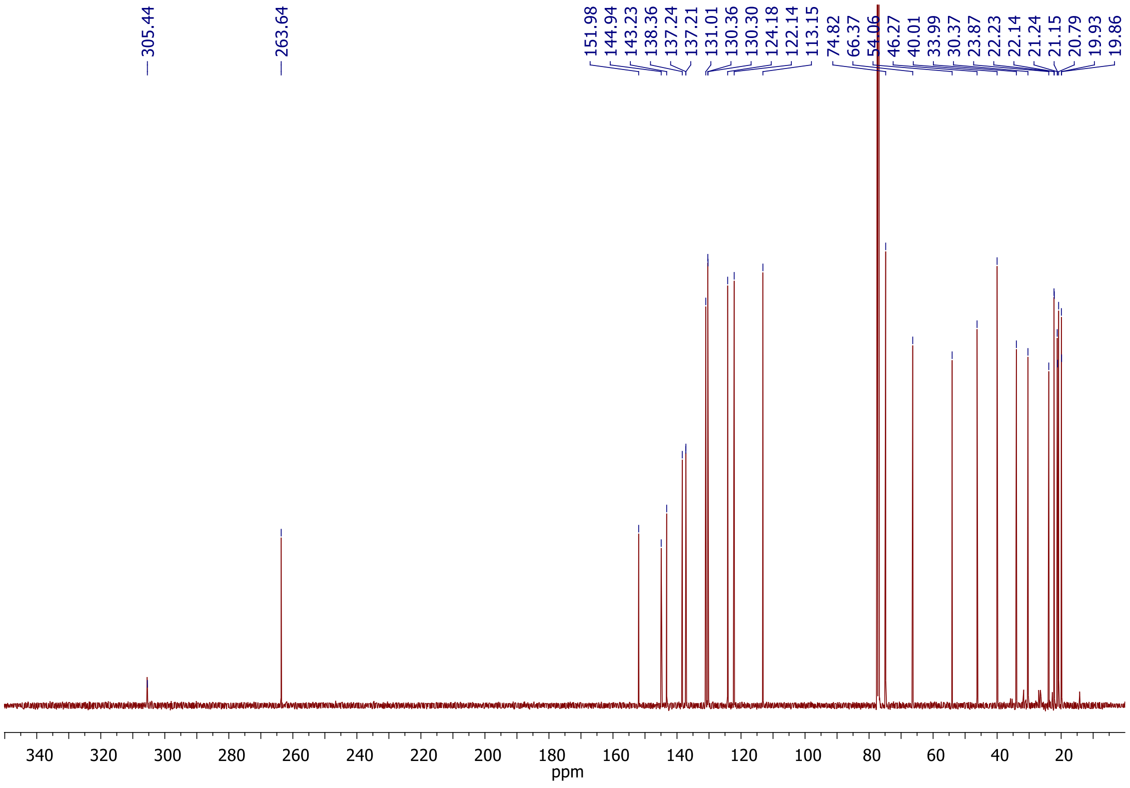

Fig. S22. ^{13}C NMR spectra of complex **18**. Solvent: $CDCl_3$

HRMS: calculated m/z: 554.1763, found: 554.1765 (for $[M\text{-}Cl]^+$: $C_{29}H_{39}NOClRu^+$).

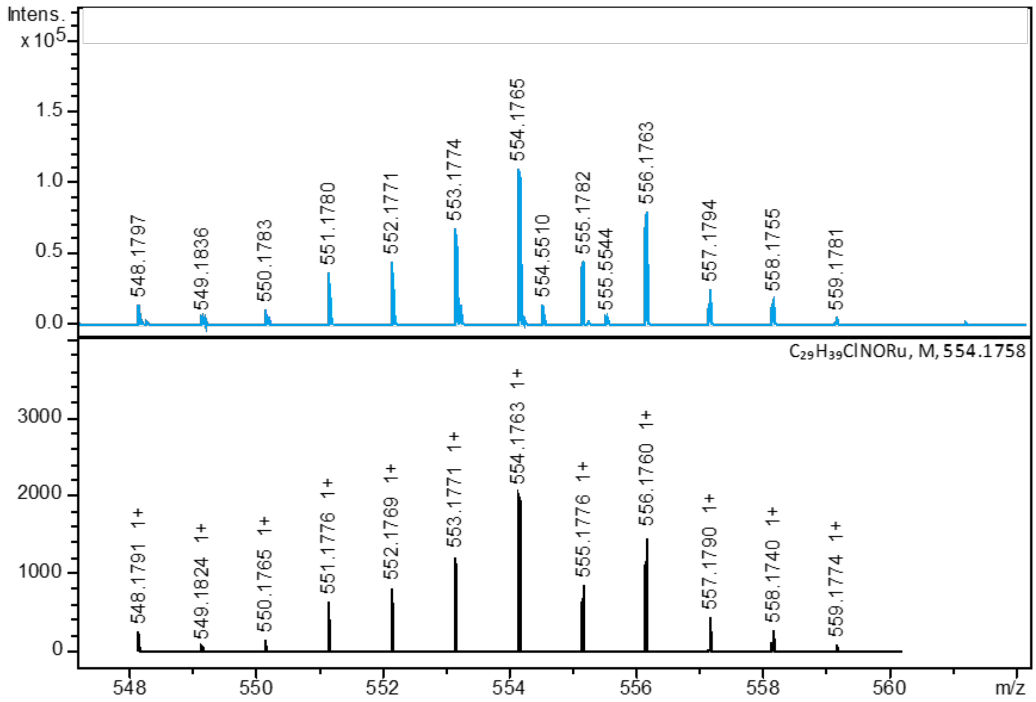

Fig. S23. ESI-MS mass spectrum of complex **18**. Measured (top) and calculated (bottom) masses and isotopic distributions.

*2.3.4. Complex **19***

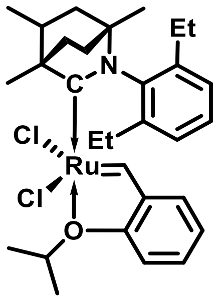

19

{2-[2,6-Diethylphenyl]-1,4,5-trimethyl-2-azabicyclo[2.2.2]octan-3-ylidene}{2-isopropoxy-benzylidene}ruthenium(II) dichloride (^{Me}BICAAC^{Et2}-HG complex, **19**): green solid, 20.2 mg, yield: 20%.

^{1}H NMR (500 MHz, $CDCl_3$) δ: 16.18 (s, 1H, Ru=C*H*), 7.58 (t, *J* = 7.7 Hz, 1H), 7.55 – 7.50 (m, 1H), 7.40 (d, *J* = 7.7 Hz, 2H), 6.90 (d, *J* = 8.3 Hz, 1H), 6.88 – 6.82 (m, 2H), 5.13 (sept, *J* = 6.1 Hz, 1H, CH_{iPrO}), 2.82 (s, 3H, C-Me_{BICAAC}), 2.80 – 2.71 (m, 1H, CH_{Et}), 2.67 – 2.56 (m, 2H, CH_{Et}), 2.57 – 2.36 (m, 3H, 1H of CH_{Et} and 2H of CH_{BICAAC}), 2.11 (dd, *J* = 12.9, 10.6 Hz, 1H, CH_{BICAAC}), 2.07 – 1.99 (m, 1H, CH_{BICAAC}), 1.93 (dt, *J* = 15.5, 3.4 Hz, 1H, CH_{BICAAC}), 1.76 (d, *J* = 6.1 Hz, 3H, Me_{iPrO}), 1.71 (d, *J* = 6.1 Hz, 3H, Me_{iPrO}), 1.74 – 1.66 (m, *J* = 6.1 Hz, 1H, CH_{BICAAC}), 1.58 – 1.50 (m, 1H, CH_{BICAAC}), 1.35 (d, *J* = 7.0 Hz, 3H, CH-Me_{BICAAC}), 1.14 (t, *J* = 7.5 Hz, 3H, Me_{Et}), 1.04 (t, *J* = 7.5 Hz, 3H, Me_{Et}), 1.00 (s, 3H, C-Me_{BICAAC}).

^{13}C NMR (126 MHz, $CDCl_3$) δ: 304.14 (Ru=*C*H), 264.12 (BICAAC carbene), 152.10, 144.74, 144.45, 142.57, 142.37, 130.99, 128.74, 126.54, 126.35, 123.99, 122.07, 113.17, 74.87, 66.41, 54.29, 45.67, 40.10, 33.69, 30.35, 24.66, 23.97, 23.87, 22.42, 22.18, 21.21, 19.99, 13.73.

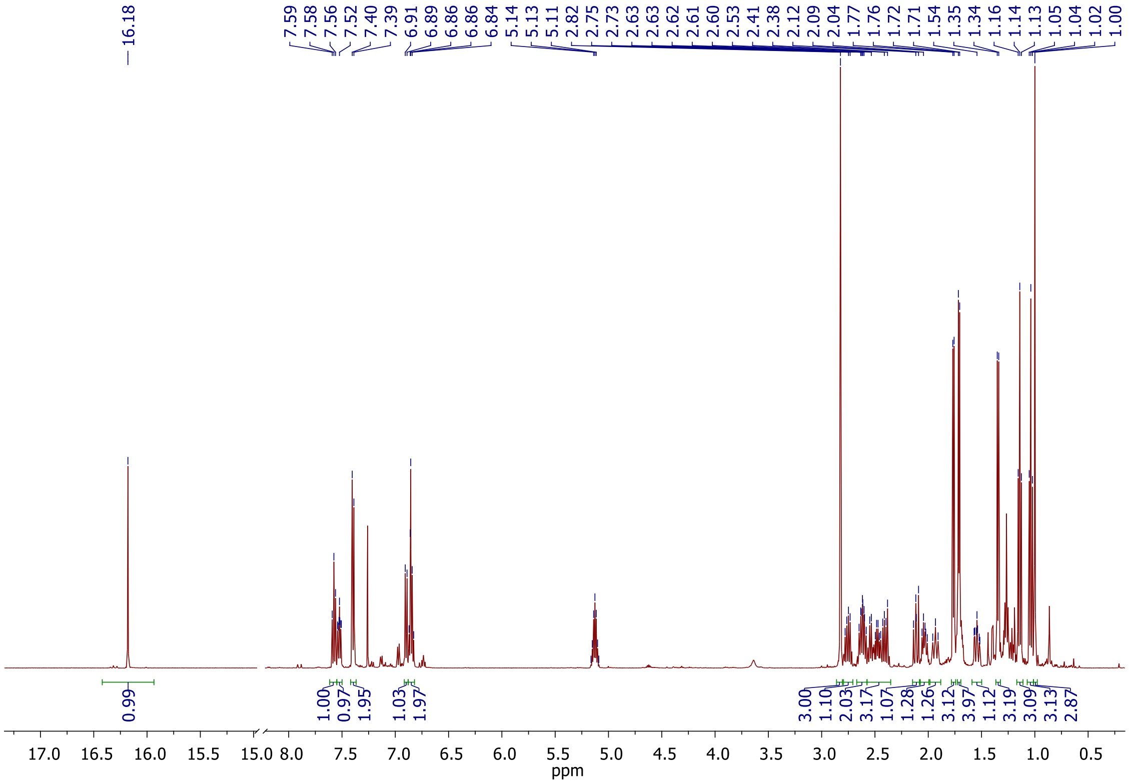

Fig. S24. ^{1}H NMR spectra of complex **19**. Solvent: $CDCl_3$

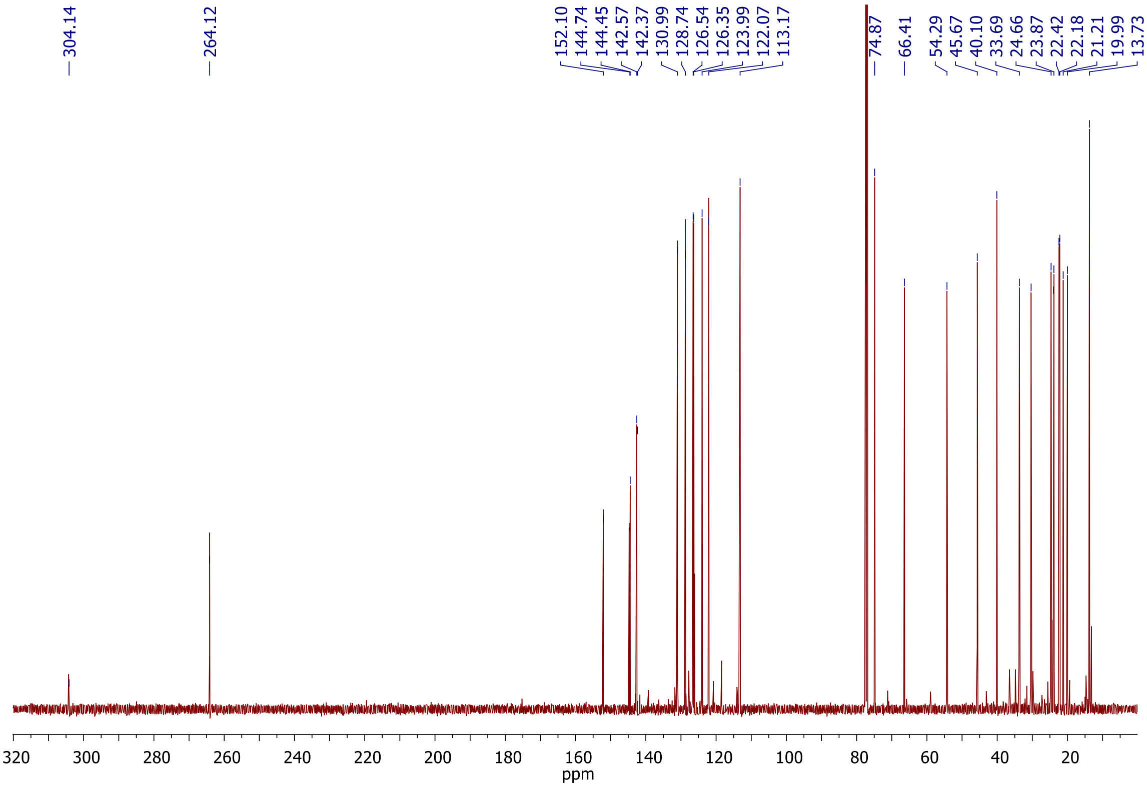

Fig. S25. ^{13}C NMR spectra of complex **19**. Solvent: $CDCl_3$

HRMS: calculated m/z: 568.1920, found: 568.1921 (for $[M-Cl]^+$:$C_{30}H_{41}NOClRu^+$).

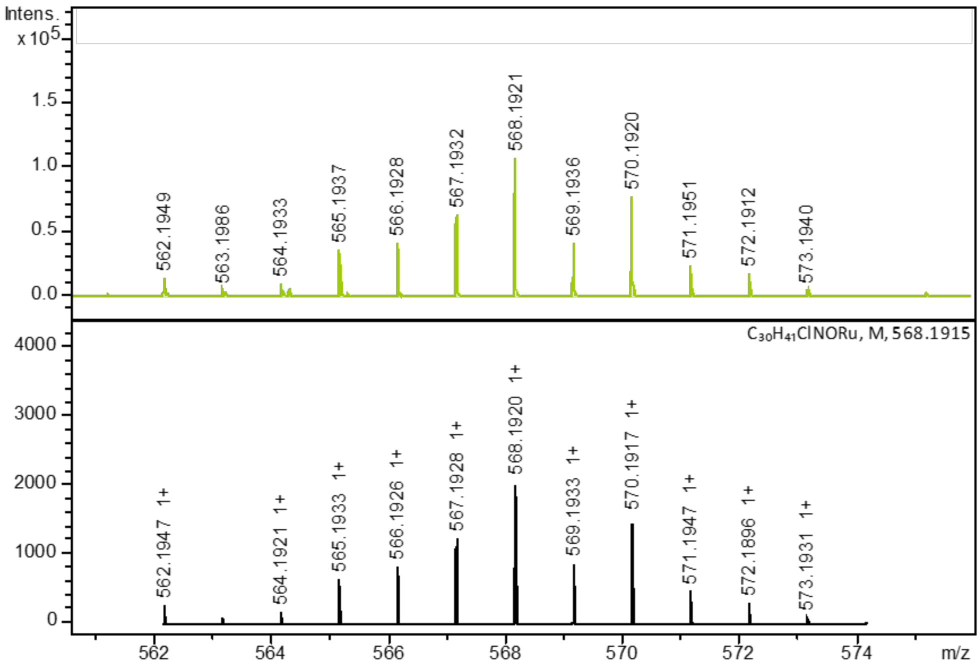

Fig. S26. ESI-MS mass spectrum of complex **19**. Measured (top) and calculated (bottom) masses and isotopic distributions.

2.4. *Synthesis of bis-BICAAC-Ru complexes* ***16*** *and* ***20***

Following the synthetic procedure reported by our group recently,[7] *bis*-carbene complexes were prepared starting from the Grubbs 1st generation complex (**G1**). The phosphine (tricyclohexylphosohine) – carbene exchange was carried out in glovebox using THF solution of the *in-situ* generated carbene by reacting the precursor salt with a strong base, lithium(hexamethyldisilyl)amide (LiHMDS).

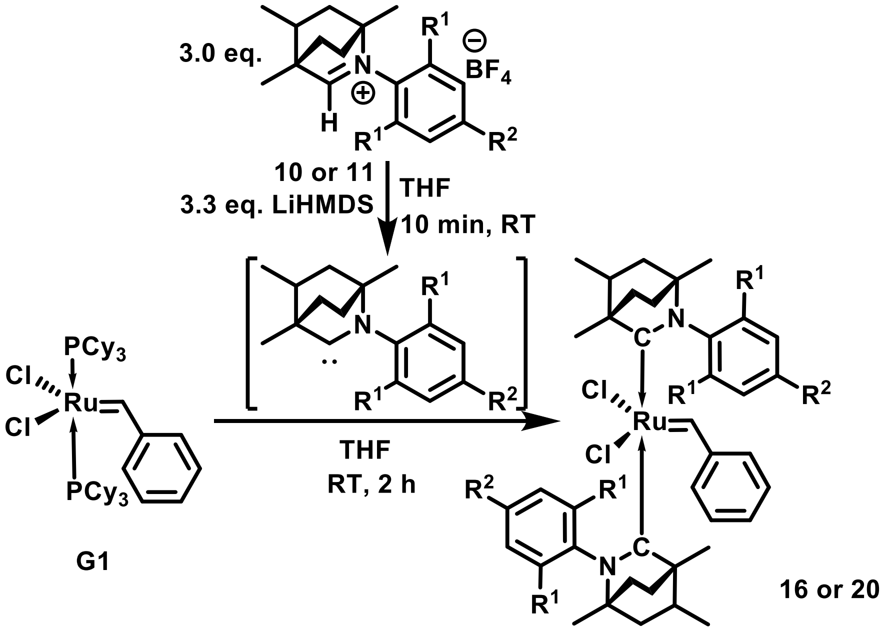

10 and 16: R^1 = Me, R^2 = NMe_2
11 and 20: R^1 = R^2 = Me

Synthesis of *bis*-carbene complexes **16** and **20**

In glovebox, Grubbs first generation complex (**G1**, 122 μmol, 100.0 mg) and the HBF_4-salt of the BICAAC precursor (365 μmol, 141.0 mg **10**, 130.3 mg **11**) were measured into two different vials. 5-5 mL of dry THF was added to both compounds forming a suspension (**10** or **11**) and a solution of **G1**. The LiHMDS (1 M in THF, 402 μmol, 402 μL) was added to the stirred suspension of the BICAAC precursors at room temperature, resulting a clear solution immediately, indicating the formation of the free carbene. Depending on the substrate, it may be brown (**10**) or yellow (**11**). This solution was added to the stirred solution of the **G1** complex yielding a brownish solution, which was stirred over 2 hours. The conversion of the reaction can be followed by ^{1}H and ^{31}P NMR.

The resulted solution was filtered through an alumina pad, then it was concentrated at reduced pressure. The complex can be purified by column chromatography. The crude material was taken up in hexane and layered on top of the column under air. Hexane containing 0–50 vol.% ethyl acetate was used as eluent over alumina as stationary phase. The yellow fractions were collected and concentrated, affording the products as yellow solids upon evaporation of the solvent.

*2.4.1. Complex **16***

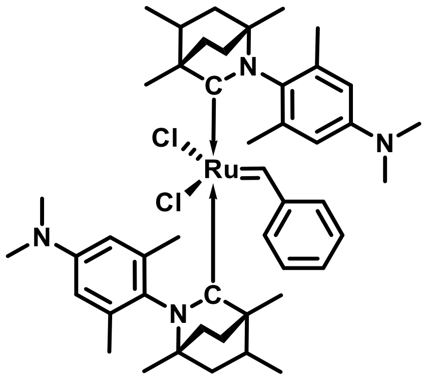

16

Bis{2-[2,6-dimethyl-4-dimethylaminophenyl]-1,4,5-trimethyl-2-azabicyclo[2.2.2]octan-3-ylidene}{benzylidene}ruthenium(II) dichloride (*bis*-^{Me}BICAAC^{NMe2} complex, **16**): yellow solid, 40.6 mg, yield: 39%. Due to the diversity of the possible diastereomers, the exact assignation is not possible, rather approx. shifts of some characteristic signals are given below. As such, at least 5 of them are observable at the temperature of recording (20 °C).

^{1}H NMR (500 MHz, $CDCl_3$,) δ: 18.70, 18.66, 18.10, 18.07 and 18.06 (s, Ru=C*H*), 9.50 – 3.50 (C*H*$_{Ar}$), 3.05 – 2.70 (includes N*Me*$_2$), others not specified.

^{13}C NMR (126 MHz, CD_2Cl_2) δ: 291.29, 282.34, 282.07, 281.86, 281.21, 281.03, 179.62, 173.67, 151.57, 150.55, 150.29, 150.22, 150.07, 149.61, 149.45, 149.20, 149.16, 149.11, 149.01, 148.63, 148.55, 148.48, 142.53, 141.94, 140.18, 139.49, 139.46, 139.20, 138.13, 138.03, 137.32, 136.50, 136.27, 136.20, 136.09, 135.93, 135.82, 135.19, 134.94, 134.90, 134.84, 134.80, 134.18, 134.02, 133.04, 132.85, 132.71, 131.80, 131.55, 131.11, 130.98, 130.43, 129.26, 129.18, 129.05, 128.94, 128.89, 128.53, 128.22, 128.15, 128.04, 127.60, 127.31, 127.25, 127.18, 127.04, 126.98, 126.41, 126.37, 114.53, 114.19, 113.85, 113.70, 113.63, 113.52, 113.44, 113.30, 113.26, 113.07, 113.01, 112.87, 112.59, 112.47, 111.91, 111.77, 111.67, 111.54, 111.12, 111.09, 67.50, 67.27, 66.92, 66.68, 66.45, 66.29, 65.80, 64.95, 57.61, 57.37, 57.13, 52.74, 48.40, 47.96, 46.67, 45.81, 41.88, 41.78, 41.17, 41.09, 41.07, 41.01, 40.98, 40.66, 40.56, 40.51, 40.35, 40.26, 40.15, 40.10, 40.06, 39.53, 39.26, 38.80, 38.63, 36.79, 36.74, 35.93, 35.26, 35.00, 34.47, 34.10, 32.64, 32.59, 31.17, 30.29, 28.56, 27.16, 26.81, 26.54, 25.80, 25.56, 25.42, 25.01, 24.85, 24.74, 24.63, 22.49, 22.23, 21.82, 21.63, 21.54, 21.46, 21.30, 20.91, 20.61, 20.52, 20.34.

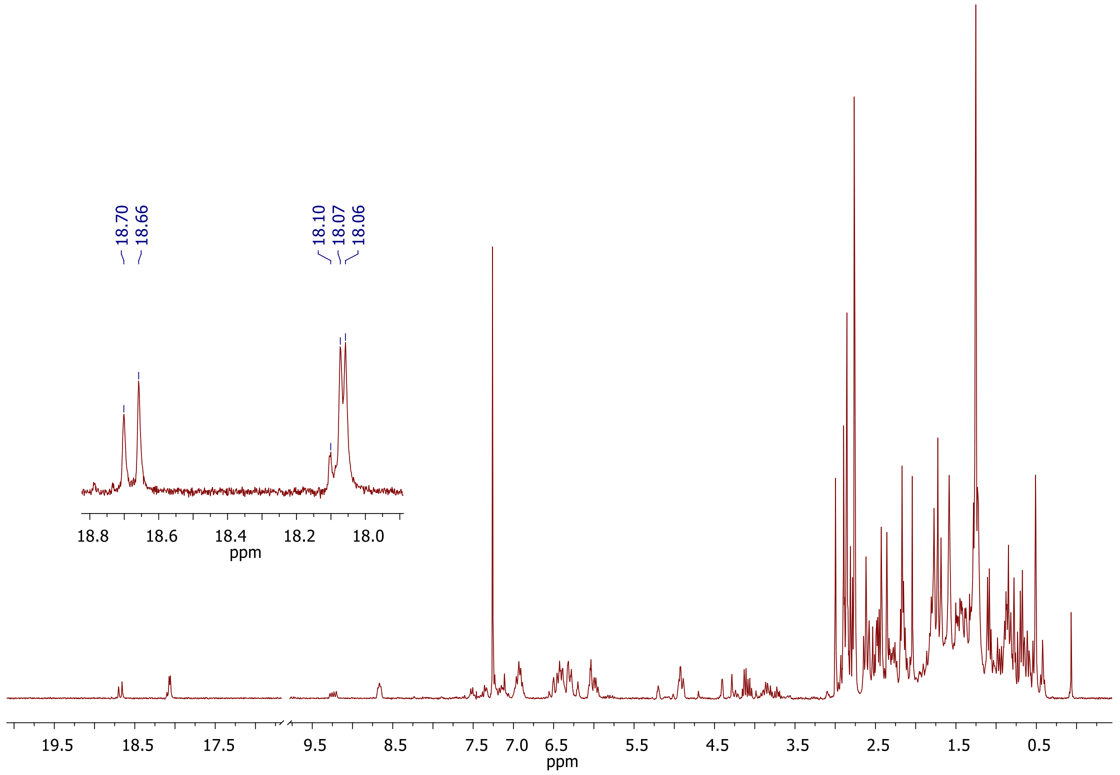

Fig. S27. ^{1}H NMR spectra of complex **16**. The characteristic benzylidene (Ru=C*H*-Ph) section is detailed. Solvent: $CDCl_3$

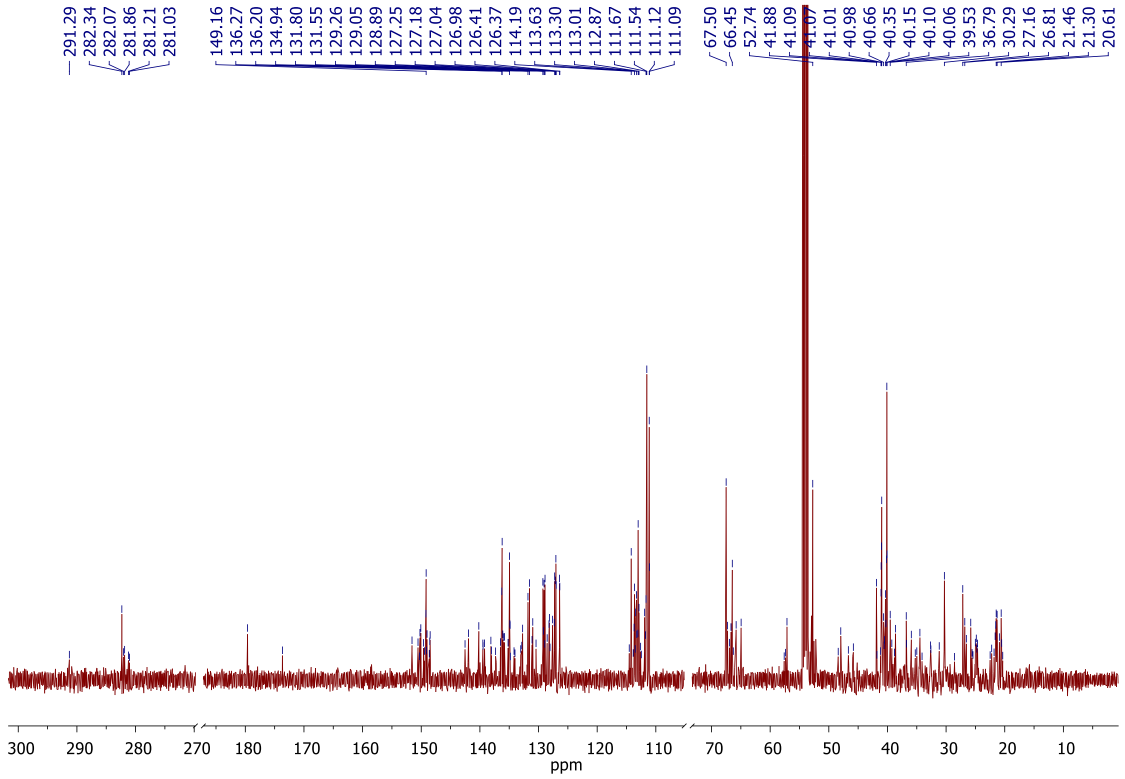

Fig. S28. ^{13}C NMR spectra of complex **16**. Solvent: CD_2Cl_2

HRMS: calculated m/z: 823.4024, found: 823.4024 (for $[M-Cl]^+$ $C_{47}H_{66}ClN_4Ru^+$).

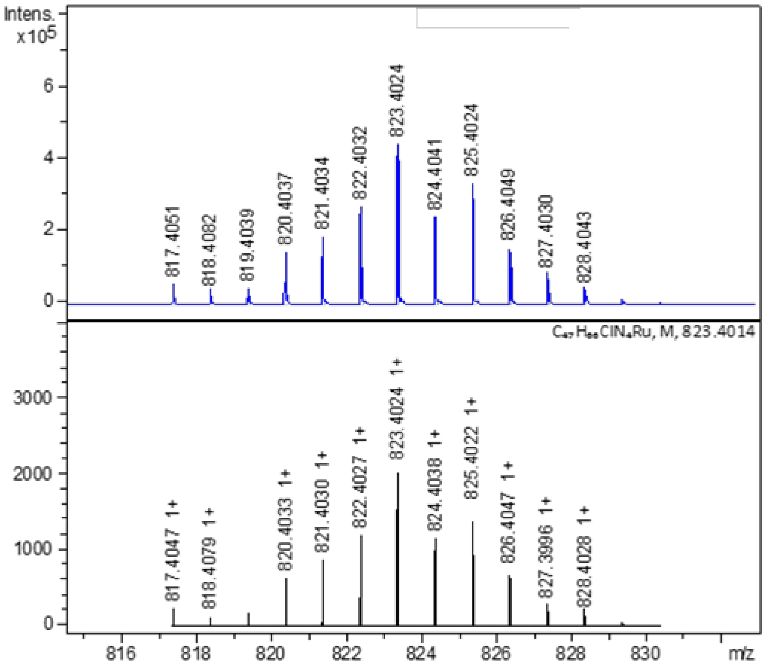

Fig. S29. ESI-MS mass spectrum of complex **16**. Measured (top) and calculated (bottom) masses and isotopic distributions.

*2.4.2. Complex **20***

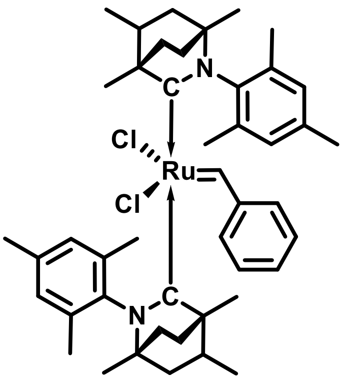

20

Bis{2-[2,4,6-trimethylphenyl]-1,4,5-trimethyl-2-azabicyclo[2.2.2]octan-3-ylidene}{benzylidene}ruthenium(II) dichloride (*bis*-^{Me}BICAAC^{mes} complex, **20**): yellow solid, 38.8 mg, yield: 40%. Due to the diversity of the possible diastereomers, the exact assignation is not possible, rather approx. shifts of some characteristic signals are given below. As such, at least 7 of them are observable at the temperature of the recording (20 °C).

^{1}H NMR (500 MHz, CD_2Cl_2) δ: 18.84, 18.77, 18.75, 18.70, 18.18, 18.16, 18.13 (s, Ru=C*H*), signals in domains between 9.26 – 9.12 and 8.72 – 8.60 probably stand for C$\underline{H}_{Bzy(ortho)}$,[7] 7.58 – 6.43 (C$\underline{H}_{Ar}$), 5.85 – 5.65 (C$\underline{H}_{Ar}$), 5.46 – 5.34 (C$\underline{H}_{Ar}$), 2.67 – 2.08 (includes C-$\underline{Me}_{BICAAC}$ and $\underline{Me}_{Ar}$), 2.08 – 1.12 (unspecified), 1.12 – 0.34 (here, the 7 doublets probably stand for CH-$\underline{Me}_{BICAAC}$).

^{13}C NMR (126 MHz, CD_2Cl_2) δ: 292.62, 292.06, 281.53, 281.30, 281.24, 280.50, 280.17, 179.35, 175.29, 151.19, 149.63, 149.49, 143.49, 143.24, 143.14, 142.96, 141.83, 141.28, 138.89, 138.56, 138.33, 138.15, 137.75, 137.70, 137.64, 137.54, 137.48, 137.40, 137.38, 137.31, 136.67, 136.56, 135.94, 135.62, 135.54, 135.45, 135.34, 135.24, 134.42, 134.36, 134.34, 134.29, 132.83, 132.73, 132.05, 131.90, 131.65, 131.18, 131.05, 130.95, 130.81, 130.38, 130.35, 130.05, 129.85, 129.72, 129.61, 129.58, 129.43, 129.35, 129.26, 129.21, 129.17, 129.03, 128.23, 128.17, 127.75, 127.41, 127.04, 126.97, 126.93, 126.79, 126.73, 126.62, 126.58, 126.38, 126.33, 126.30, 67.34, 66.99, 66.73, 66.52, 66.45, 66.32, 59.53, 57.96, 56.67, 53.01, 52.78, 52.71, 52.47, 52.36, 52.21, 52.12, 48.25,

47.81, 46.65, 46.60, 46.53, 46.44, 46.27, 46.05, 45.97, 45.20, 43.31, 41.92, 41.39, 39.51, 39.34, 39.25, 39.06, 38.89, 38.72, 38.50, 37.25, 37.13, 36.85, 35.35, 35.24, 35.17, 34.82, 34.65, 34.51, 34.18, 34.07, 33.72, 33.27, 33.14, 32.95, 32.75, 32.66, 32.63, 32.51, 32.19, 31.88, 31.75, 31.10, 30.58, 30.29, 29.96, 28.46, 27.16, 26.85, 26.48, 25.85, 25.72, 25.37, 24.85, 24.77, 24.72, 24.63, 24.55, 24.17, 24.10, 23.80, 23.29, 23.25, 22.19, 21.71, 21.30, 21.25, 21.19, 21.15, 21.09, 21.00, 20.98, 20.90, 20.87, 20.80, 20.72, 20.69, 20.59, 20.35, 20.25, 20.03, 20.00, 19.91, 19.64, 19.55, 19.36, 16.00, 15.76, 14.46.

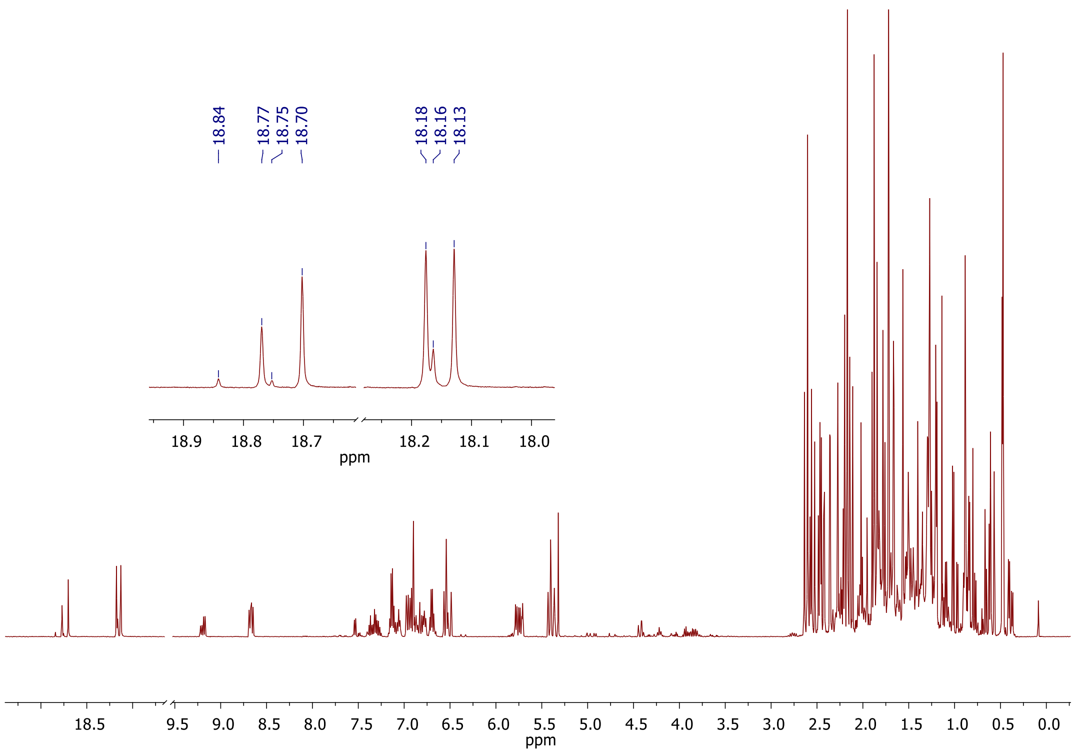

Fig. S30. ^{1}H NMR spectra of complex **20**. The characteristic benzylidene (Ru=C<u>*H*</u>-Ph) section is detailed. Solvent: CD_2Cl_2

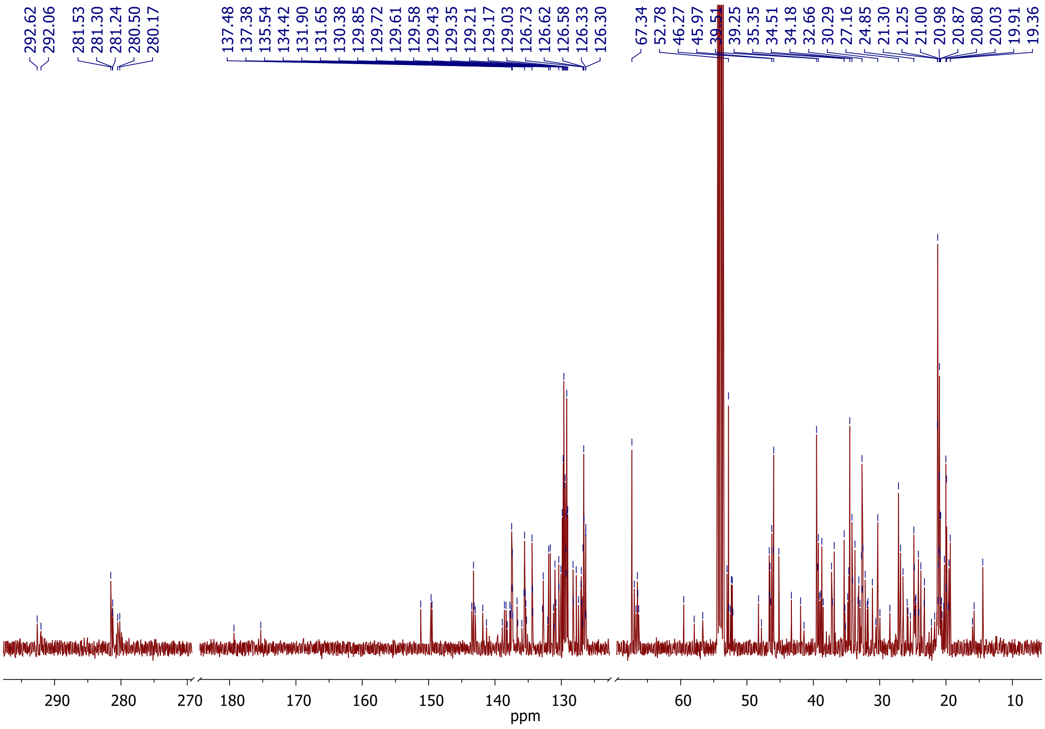

Fig. S31. ^{13}C NMR spectra of complex **20**. Solvent: CD_2Cl_2

HRMS: calculated m/z: 765.3492, found: 765.3491 (for $[M-Cl]^+$ $C_{45}H_{60}N_2ClRu^+$).

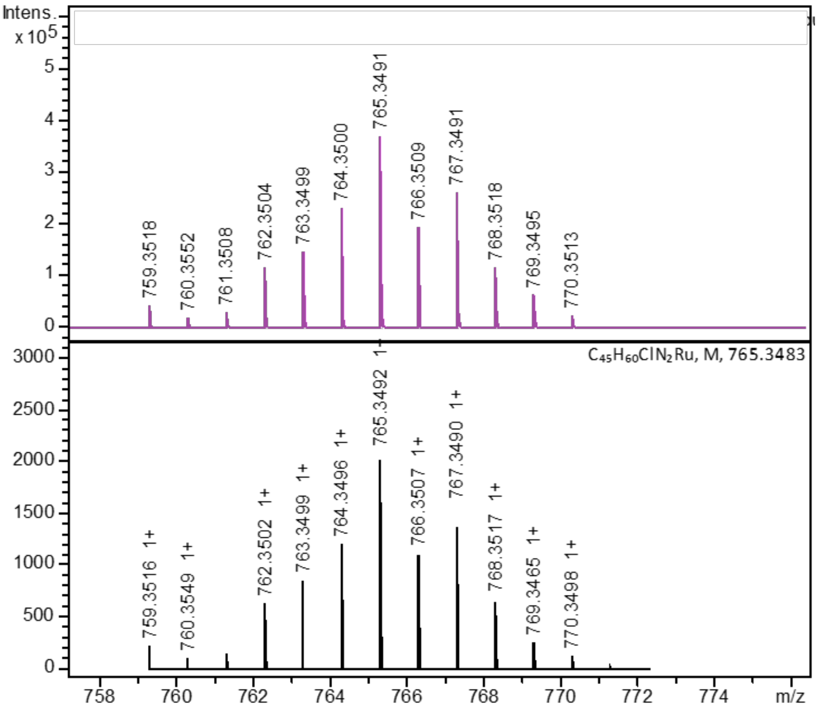

Fig. S32. ESI-MS mass spectrum of complex **20**. Measured (top) and calculated (bottom) masses and isotopic distributions.

*2.5. Synthesis of ionic mono-BICAAC-Ru complex **15***

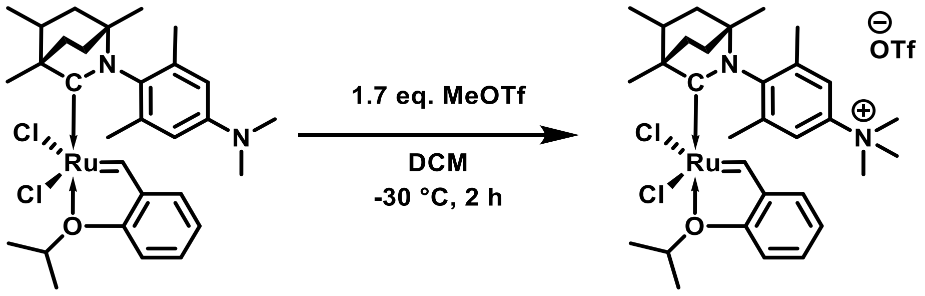

14 **15**

Synthesis of complex **15**

An oven-dried Schlenk flask was charged with complex **14** (0.162 mmol, 100.0 mg) and dissolved in 2 mL of DCM. Then, it was cooled down to –30°C by using a mixture of acetone/dry ice. Methyl trifluormethanesulfonate (0.275 mmol, 30 μL) was added dropwise through a septum to the stirred DCM solution of **14**. After 15 minutes, the flask was allowed to warm up to room temperature. An hour later, green precipitate formed. Evaporation of the solvent and the alkylation agent resulted the product (**15**) as green solid (106.3 mg, 84%).

^{1}H NMR (500 MHz, acetone-d_6) δ: 16.32 (s, 1H, Ru=C$\underline{H}$), 8.23 – 8.15 (m, 2H, C$\underline{H}_{Ar}$), 7.68 (t, J = 7.7 Hz, 1H, C$\underline{H}_{Bzy}$), 7.21 (d, J = 8.4 Hz, 1H, C$\underline{H}_{Bzy}$), 6.96 – 6.87 (m, 2H, C$\underline{H}_{Bzy}$), 5.29 (sept, J = 6.0 Hz, 1H, C$\underline{H}_{iPrO}$), 4.12 (s, 9H, N$\underline{Me_3}^+$), 2.81 (s, 3H, C-$\underline{Me}_{BICAAC}$), 2.47 (s, 3H, $\underline{Me}_{Ar}$), 2.46 – 2.41 (m, 1H, C$\underline{H}_{BICAAC}$), 2.38 (s, 3H, $\underline{Me}_{Ar}$), 2.30 (dd, J = 13.4, 10.6 Hz, 1H, C$\underline{H}_{BICAAC}$), 2.20 – 2.10 (m, 1H, C$\underline{H}_{BICAAC}$), 1.99 – 1.85 (m, 2H, C$\underline{H}_{BICAAC}$), 1.72 (d, J = 3.2 Hz, 3H, $\underline{Me}_{iPrO}$), 1.71 (d, J = 3.2 Hz, 3H, $\underline{Me}_{iPrO}$), 1.70 – 1.65 (m, 1H, C$\underline{H}_{BICAAC}$), 1.65 – 1.58 (m, 1H, C$\underline{H}_{BICAAC}$), 1.29 (d, J = 7.2 Hz, 3H, CH-$\underline{Me}_{BICAAC}$), 1.05 (s, 3H, C-$\underline{Me}_{BICAAC}$).

^{13}C NMR (126 MHz, acetone-d_6) δ: 300.65 (Ru=$\underline{C}$H), 266.26 (BICAAC carbene), 153.08, 147.93, 147.39, 145.61, 141.38, 141.29, 136.76, 132.05, 128.42, 124.82, 123.10, 122.27, 122.19, 121.26, 115.57, 114.23, 75.84, 71.94, 68.15, 58.22, 57.89, 54.91, 46.74, 40.47, 34.35, 26.37, 22.40, 22.37, 22.18, 22.12, 21.36, 21.34, 20.52, 20.25.

^{19}F NMR (282 MHz, acetone-d_6) δ: -78.83 (OTf$^-$).

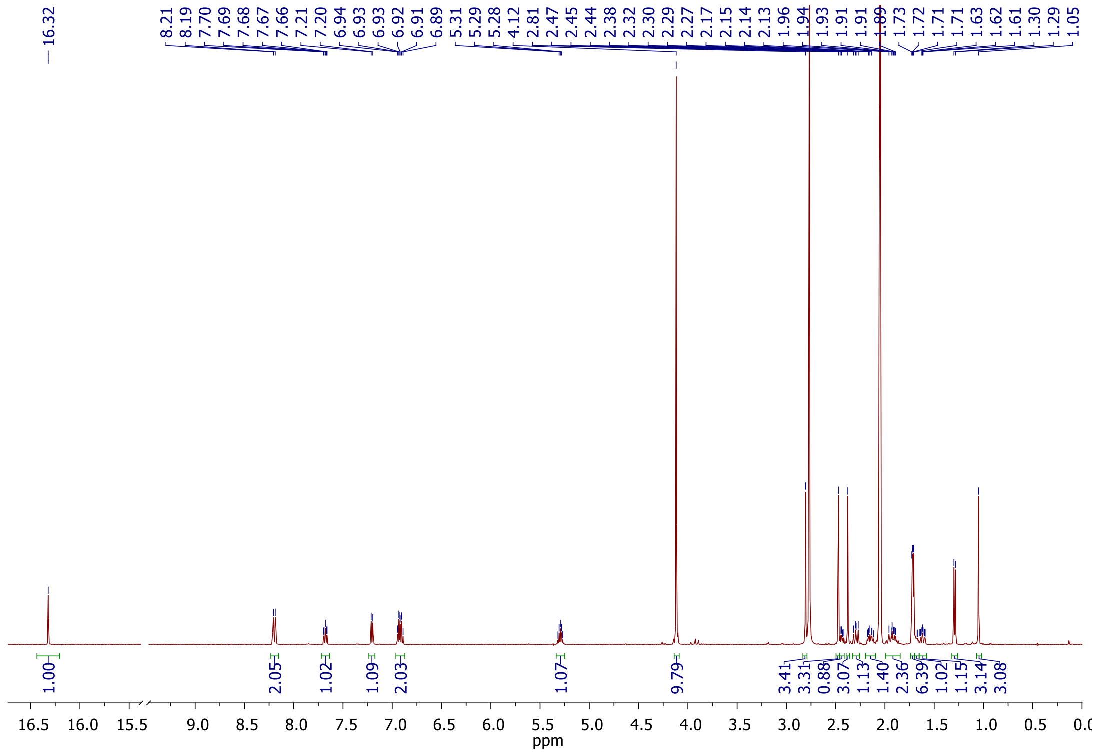

Fig. S33. ^{1}H NMR spectra of complex **15**. Solvent: $(CD_3)_2CO$

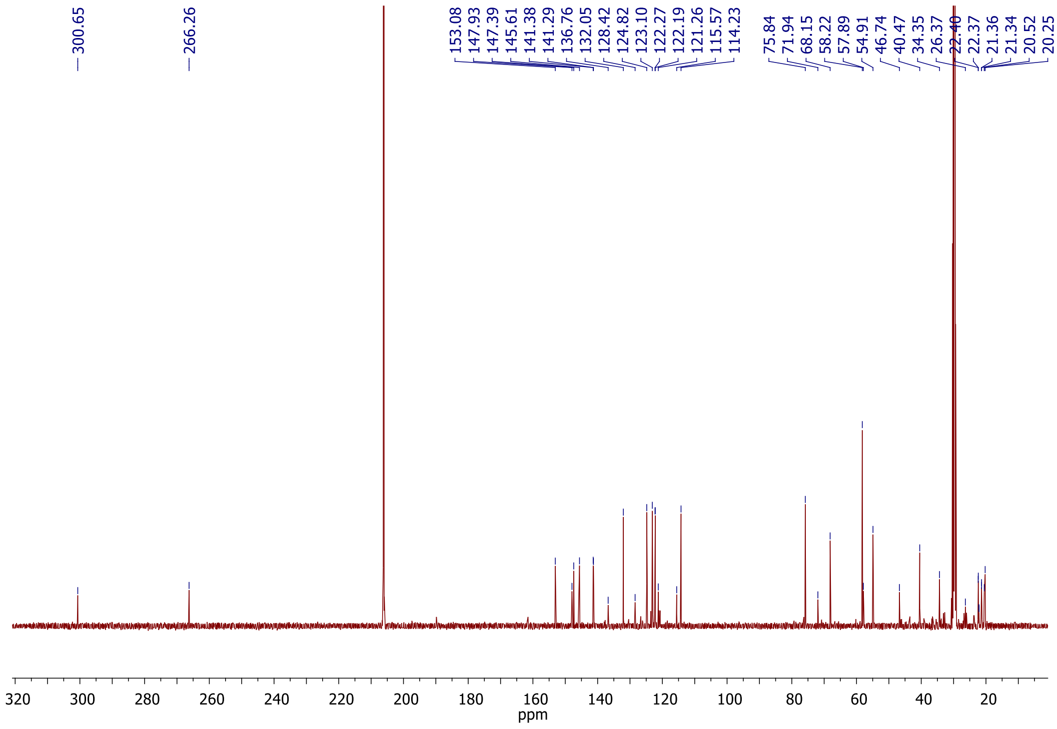

Fig. S34. ^{13}C NMR spectra of complex **15**. Solvent: $(CD_3)_2CO$

HRMS: calculated m/z: 633.1950, found: 633.1948 (for $[M-Cl]^+$ $C_{31}H_{45}N_2ClORu^+$).

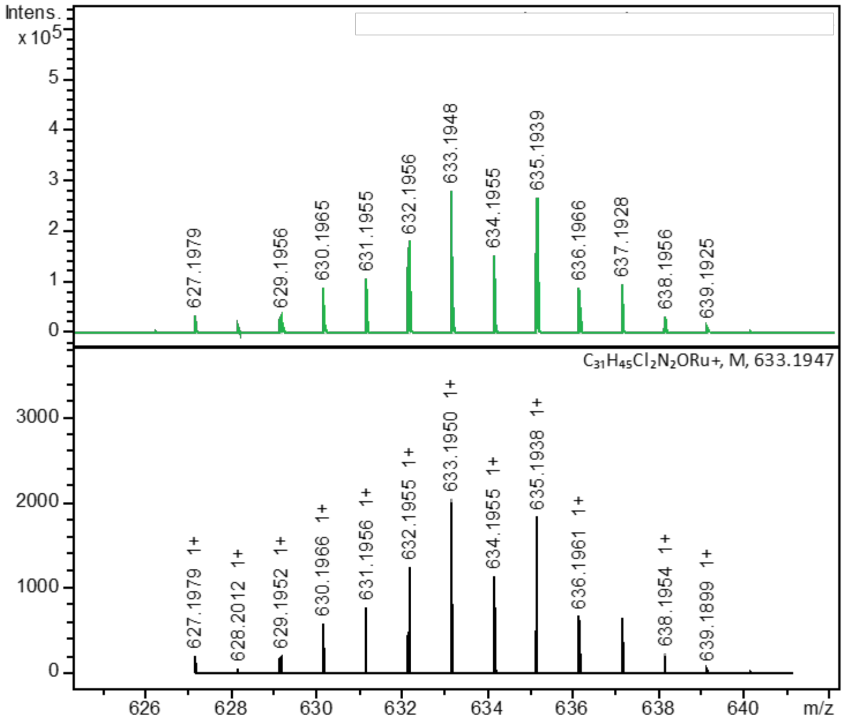

Fig. S35. ESI-MS mass spectrum of complex **15**. Measured (top) and calculated (bottom) masses and isotopic distributions.

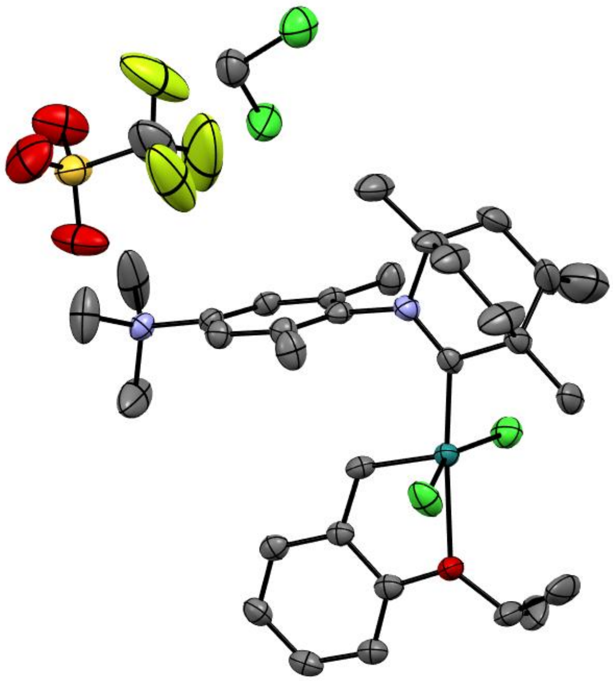

Fig. S36. X-Ray structure of complex **15**. Note that the triflate anion and one molecule of dichloromethane is also present in the unit cell

*2.6. Synthesis of ionic bis-BICAAC-Ru complex **17***

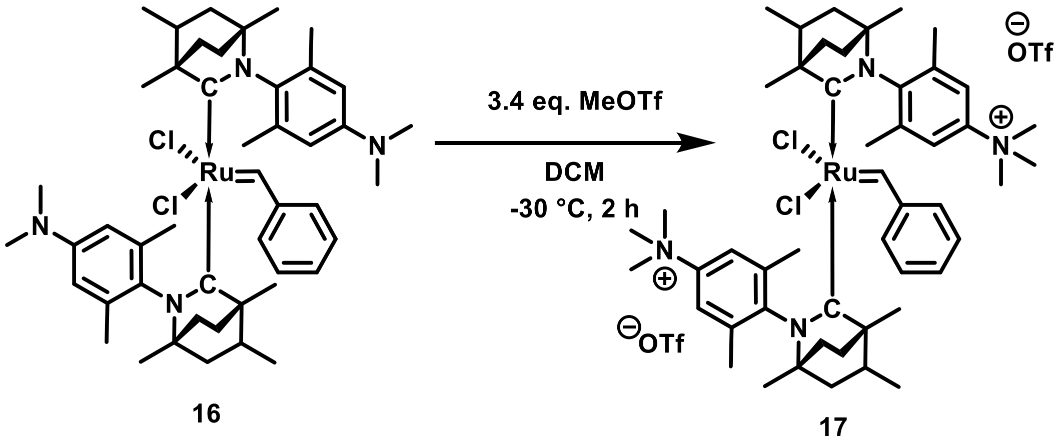

Attempt for the synthesis of complex **17**

An oven-dried Schlenk flask was charged with complex **16** (0.116 mmol, 100.0 mg) and dissolved in 2 mL of DCM. Then, it was cooled down to –30°C by using a mixture of acetone/dry ice. Methyl trifluormethanesulfonate (0.396 mmol, 43 µL) was added dropwise through a septum to the stirred DCM solution of **16**. After 30 minutes, the flask was allowed to warm up to room temperature. No precipitate formed. Evaporation of the solvent and the alkylation agent resulted the crude product of **17** as yellow solid.

Although signals of the benzylidene positioned proton (Ru=C*H*) showed clear upshift (indicating full conversion of **16**), an approximate 20mol% loss of carbene ligand was detected in ^{1}H NMR: a characteristic signal (singlet between 10 and 9 ppm) of protonated species – similar to precursor **10** – appeared. The formed side products may render similar chemically and physical properties to the target compound (*e.g.* the *N*-methylated derivative of **10**), therefore the isolation and full characterization of the target complex did not succeed.

3. Catalytic experiments

*3.1. Representative example of the RCM of diethyl diallylmalonate (**21**) in toluene-d_8 at 50 °C or 75 °C (0.05 mol% catalyst load)*

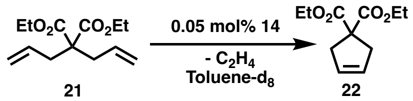

In a glovebox, screwcap NMR tube was charged with 29 uL (0.12 mmol) diethyl diallylmalonate (**21**) and 0.6 mL toluene-d_8. The sample temperature was equilibrated (at 50 or 75 °C) and the starting point was measured. The sample was removed from the spectrometer and a solution of the catalyst (**14**, 0.05 mol%, 0.038 mg in 30 uL toluene-d_8) was added. The sample was placed back in the spectrometer and ^{1}H NMR transients were collected at every two minutes over 3 hours.

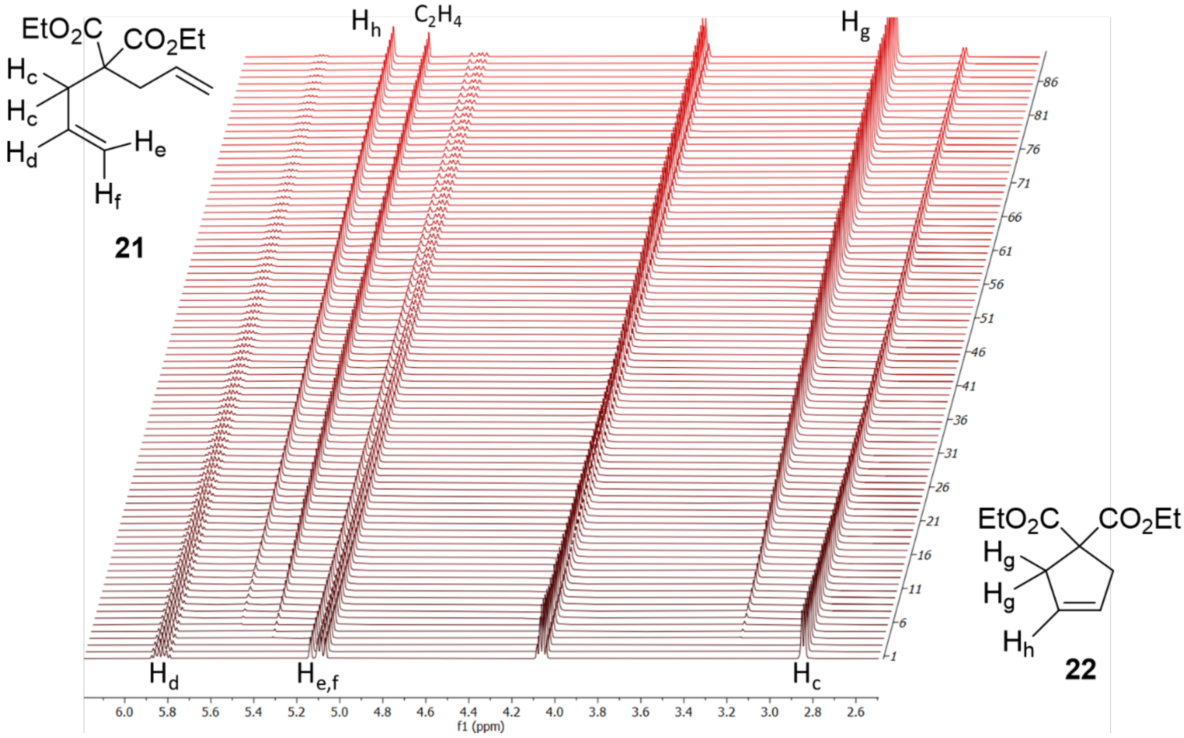

Fig. S37. Representative example of the RCM of diethyl diallylmalonate (**21**) in toluene-d_8 (75 °C, 3 h, 0.05 mol% **14**)

Interestingly, the RCM kinetic curves can be described by eq. 1

$$\frac{dx}{dt} = kx^{\alpha}(1-x)^{\beta} \qquad (1)$$

where dx/dt is the rate of formation of ring closing metathesis reaction (RCM) for product **22**, x is the conversion value of **21** to **22**. k is the rate constant, α and β are the reaction orders.
Eq. 1. was solved numerically using MS Excel and the parameters of eq. 1 (*i.e.*, k, α and β) were obtained by fitting of eq. 1. to the experimental x *versus* reaction time curves using the "Solver" function of MS Excel. The fitted curves along with the experimental ones are plotted in Fig. S38.

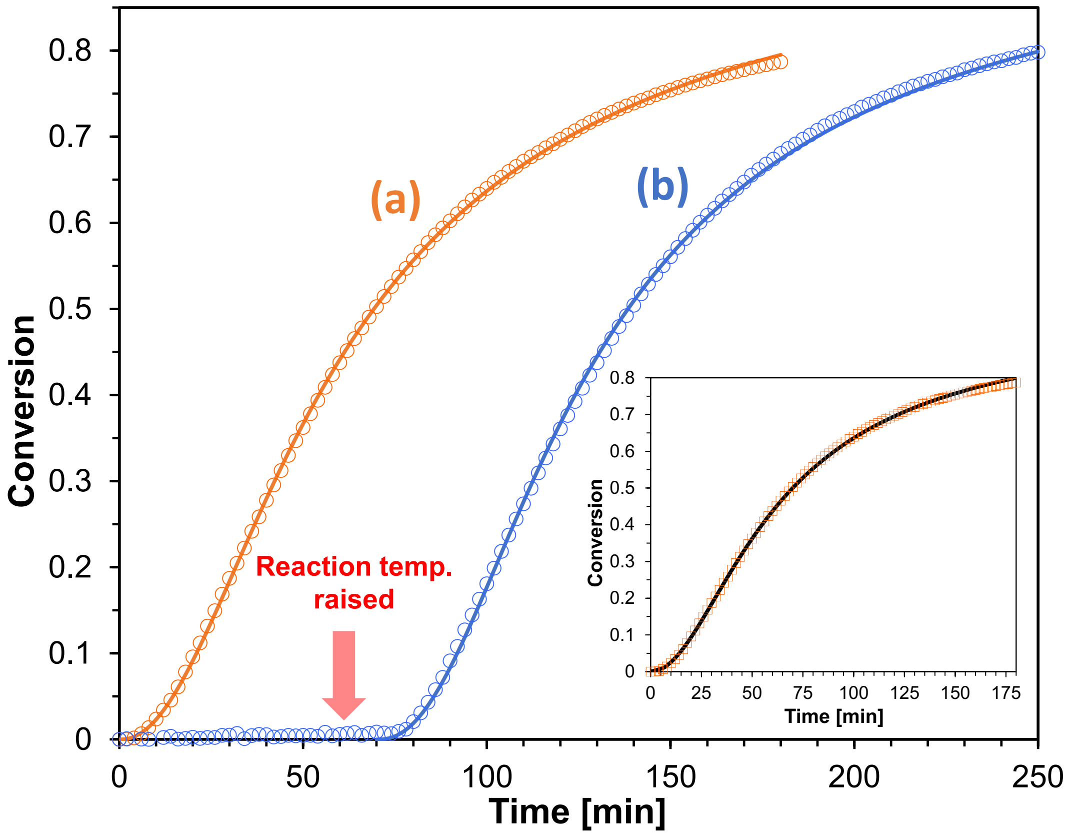

Fig. S38. The experimental and the fitted conversion *versus* reaction time curves for the ring closing metathesis reaction of **21** using catalyst **14**. The symbols and the solid lines represent the experimental and fitted curves using eq. 1., respectively. Orange symbols denote the experimental data obtained by *in-situ* NMR at 75 °C, while the blue symbols stand for the experiments thermostated for one hour at 50 °C followed by a temperature raise to 75 °C. Catalyst load (**14**): 0.05 mol%, [**21**] = 0.2 M. The inset shows the experimental and the fitted conversion curves using eq. 3. (symbols represent the experimental and black line the calculated data by eq. 3.)

According to the results of the fitting, the values of k, α and β for curve (a) were determined to be 0.035 min^{-1}, 0.51 and 2.09 respectively, while for curve (b) k = 0.039 min^{-1}, α = 0.55 and β = 2.14 were obtained. Thus, using values of α = 0.5 and β = 2, the kinetics can be approximated by eq. 2:

$$\frac{dx}{dt} = k\sqrt{x}(1-x)^2 \qquad (2)$$

and integration of eq. 2 leads to eq. 3.

$$\frac{1}{2}\ln\left(\frac{\sqrt{x}+1}{\left|\sqrt{x}-1\right|}\right) + \frac{\sqrt{x}}{1-x} = kt \qquad (3)$$

As seen in Fig. S38 inset, eq. 3. with k = 0.033 min^{-1} adequately describes the observed kinetic properly.

*3.2. Representative example of the latent properties in the RCM of diethyl diallylmalonate (**21**) in toluene-d_8 (0.25 mol% catalyst load)*

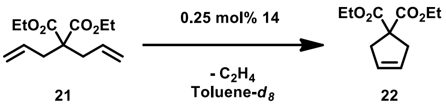

In a glovebox, screwcap NMR tube was charged with 29 uL (0.12 mmol) diethyl diallylmalonate (**21**) and 0.6 mL toluene-d_8. The sample temperature was equilibrated at 50 °C and the starting point was measured. The sample was removed from the spectrometer and a solution of the catalyst (**14**, 0.25 mol%, 0.188 mg in 37 uL toluene-d_8) was added. The sample was placed back in the spectrometer and ^{1}H NMR transients were collected over 1 hours. In one hour, the temperature was increased to 75 °C and the measurement continued for a total of 6 hours.

*3.3. RCM of diethyl diallylmalonate (**21**) with catalyst **5** in toluene-d_8 at 100 °C*

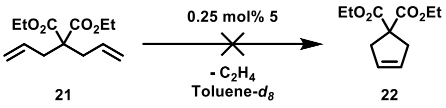

In a glovebox, a small vial was charged with 29 uL (0.12 mmol) diethyl diallylmalonate (**21**) and 0.6 mL toluene-d_8. Catalyst **5** (5 mg) was dissolved in 4 mL of toluene-d_8 and 29 uL of the catalyst solution (0.19 mg, 3*10^{-4} mmol) was added to the reaction mixture. The vial was sealed and heated up to 100 °C for 12 h. After cooling to room temperature, the solution was measured via ^{1}H NMR, which showed no RCM product (**22**) formation.

*3.4. Representative example of the ISOMET reaction of methyl oleate (**23**)*

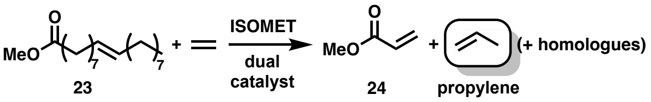

In a glovebox, Fischer-Porter bottle was charged with isomerization catalyst (**RuH**, 2 mol%, 15 mg), methyl oleate (240 mg, 0.80 mmol), toluene (3 mL) and the metathesis catalyst (**14**, 1 mol%, 5 mg). The bottle was closed in the glovebox under argon and transferred to the ethylene line, equipped with a pressure gauge. The line was vented 5 times with ethylene (99.9%) followed by the venting of the bottle 5 times. The ethylene pressure was set to 10 bar, the bottle was closed and heated to 75°C for 24 h. After the reaction mixture cooled to room temperature, sample of the gas phase was collected and measured *via* GC-FID. Part of the liquid phase was measured *via* GC-MS in the presence of anthracene as internal standard.

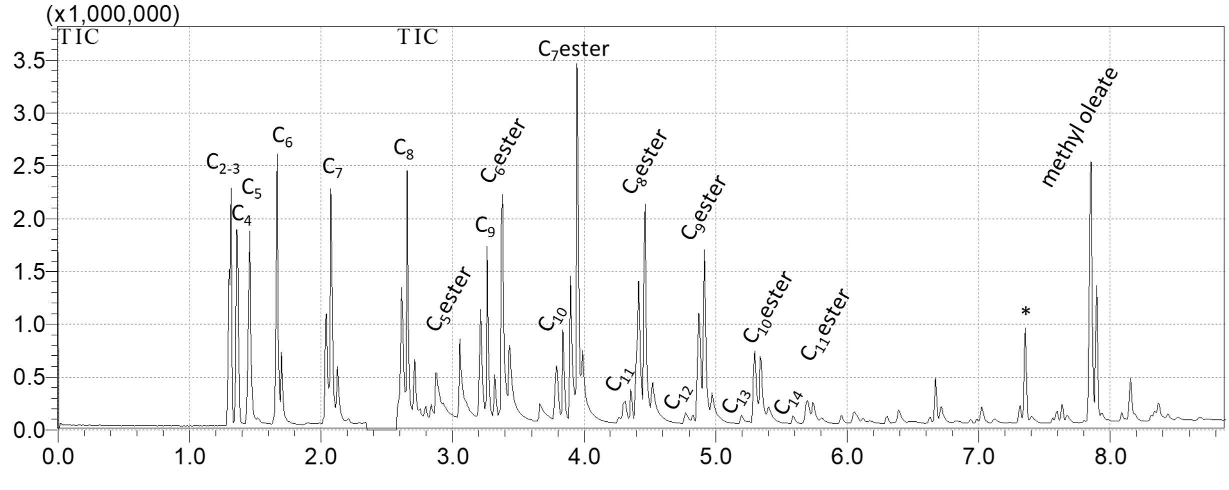

Fig. S39. GC-MS of the ISOMET reaction mixture of methyl oleate (**23**) with 1mol% **14** (C_x indicated the corresponding alkene homologues; $C_x$ester indicated the corresponding unsaturated ester homologues; * methyl palmitate, impurity of the starting material)

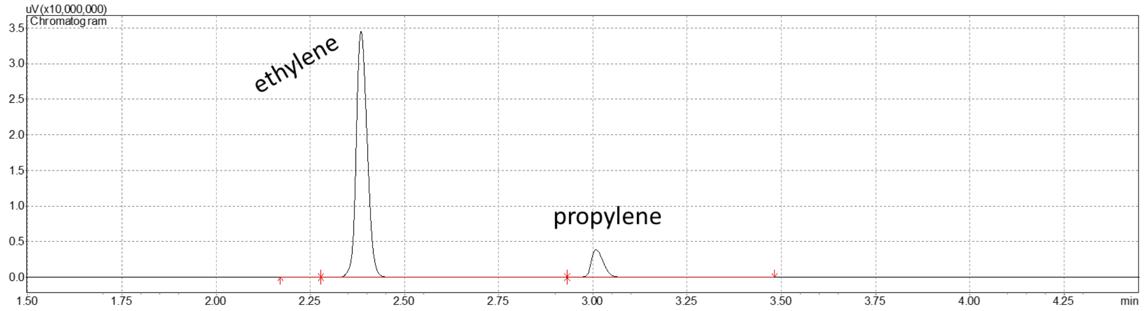

Fig. S40. GC-FID chromatogram of the gas phase of the ISOMET reaction mixture of methyl oleate (**23**) with 1mol% **14**

3.5. *Representative example of the ISOMET reaction of 1-octadecene (**25**)*

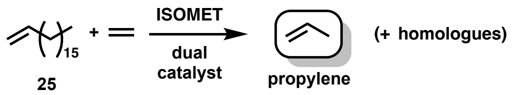

In a glovebox, Fischer-Porter bottle was charged with isomerization catalyst (**RuH**, 0.02 mol%, 3 mg), 1-octadecene (**25**, 4 g, 15.8 mmol), toluene (3 mL) and the solution of the metathesis catalyst (**14**, 0.001 mol%, 0.098 mg in 100 uL toluene). The bottle was closed in the glovebox under argon and transferred to the ethylene line, equipped with a pressure gauge. The line was vented 5 times with ethylene (99.9 or 99.95 or 99.995) followed by the venting of the bottle 5 times. The ethylene pressure was set to 10 bar, the bottle was closed and heated to 75 °C for 3 h. After 3, 6 and 24 h the reaction mixture was cooled to room temperature and the gas phase was collected for GC-FID analysis. The bottle was filled with ethylene and the heating was continued for the specified interval. After the reaction was completed, part of the liquid phase was measured *via* GC-MS in the presence of anthracene as internal standard.

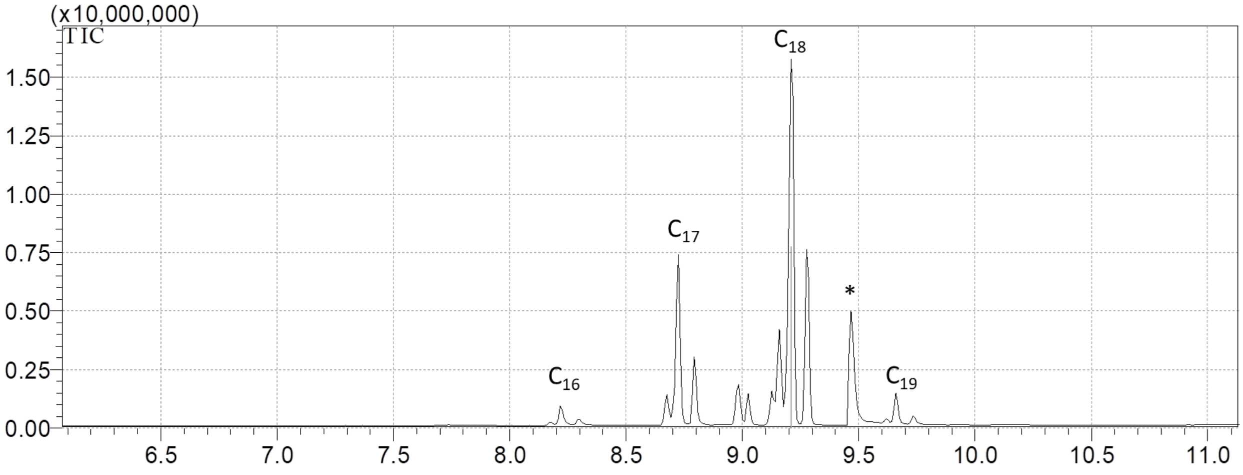

Fig. S41. GC-MS of the ISOMET reaction mixture of 1-octadecene (**25**) with 10 ppm **14** after 24h (Ethylene purity: 99.9 %, ; C_x indicated the corresponding alkene homologues; * anthracene, internal standard)

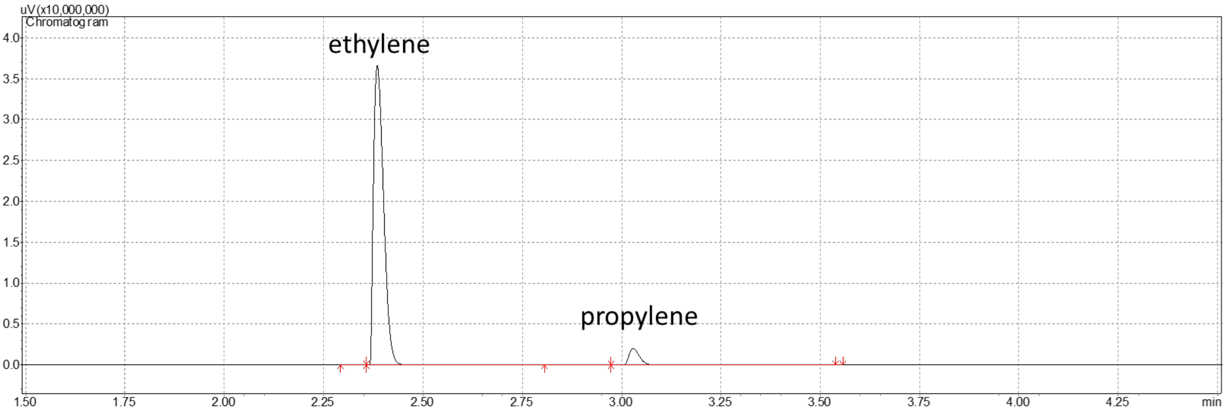

Fig. S42. GC-FID chromatogram of the gas phase of the ISOMET reaction mixture of 1-octadecene (**25**) with 10 ppm **14** after 3h (Ethylene purity: 99.9 %)

*3.6. Representative example for the CM of 1-decene with catalyst **14** in neat at 75 °C*

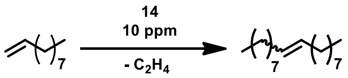

In a glovebox, a small vial was charged with 5.4 mL (28.5 mmol) 1-decene. Solution of catalyst **14** (0.18 mg, 10 ppm) in toluene was added to 1-decene and the mixture was stirred for 3 h at 75 °C. After cooling to room temperature, part of the mixture was dissolved in toluene-d_8 and measured via ^{1}H NMR.

4. X-ray structure determination

The crystal and molecular structures of compounds **14**, **18**, **20** and **21** were established unequivocally by single-crystal X-ray diffraction analysis (Figure 1). The results for the X-ray diffraction structure determinations were very good according to the Checkcif functionality of PLATON software (Utrecht University, Utrecht, The Netherlands).[4] The reasons for alert A and B level errors are the presence of very heavy element (ruthenium) and the highly irregular shape (very thin plates or needles) of the crystals. However, the deposited Crystallographic Information File (CIF) includes answers for these errors and the overall correctness of the structures is not influenced. In case of **20** because of the unusually long unit cell axis of 59.4 Å Cu Kα radiation was applied. In **21** there is a half distorted solvent hexane molecule in the asymmetric unit. Further experimental details of the crystal parameters, data collection, and results of structure refinement are given in Table S1. The very rigid BICAAC ligand induce strong constrains on the coordination of ruthenium. Selected bond length and angle data of ruthenium coordination are compiled in Table S2.

The coordination of the ruthenium center in BICAAC-Ru complexes is similar to NHC and CAAC-Ru complexes having the ruthenium and two chlorides is approximately in one plane with the coordinating benzylidene carbon, while the NHC, CAAC or BICAAC carbene and the coordinated oxygen are above and under the plane, respectively (Table S2). The C-Ru-C angles of the two BICAAC-Ru complexes are close to 105° which values are in alignment with those of the reported six-membered CAAC-6 Ru complexes.[9] However, the relative configuration of the stereogenic centers of the BICAAC ligand is the most interesting feature of the structure of these new complexes. In our case, the bicyclic nature of the BICAAC ligand decreases the number of possible diastereomers and it is also important how the crystal mirrors the achiral route of the synthesis. In **14** we found two molecules in the asymmetric unit which are mirror images of each-others with slight difference in the conformation of the phenyl rings (Figure S43). Here the glide plane of the lattice symmetry generates the opposite enantiomer of both conformer as it is expected for a racemate and the crystal is also a twin by inversion. In **5** there are three complexes in the asymmetric unit and the ratio of the enantiomers is 2:1, their overlayed structure is shown at Figure S44. Moreover, as the space group is centrosymmetric the final ratio of the enantiomers is 1:1, as it is expected for the racemate. The structure of complex **18** is the most interesting in this respect. In this case the space group (No.19) is non-centrosymmetric, Sohncke space group suitable for pure enantiomers. We found racemic conglomerate i.e. mechanical mixture of enantiomer pure crystals as well as racemic twin and also crystalline powder. For the chiral crystals the Flack parameter was very close to 0 with slight difference in conformation (Figure S45) while the inversion twin crystal gave Flack parameter of 0.5, as it is expected.

Deposition Numbers 2128047-2128050 for **14**, **18**, **5** and **20**, as well as 2144205 and 2144206 for the other enantiomer of **18** and the inversion twin crystal of **18** from the racemic conglomerate and 2144599 for **15**, respectively contain the supplementary crystallographic data for this paper. These data are provided free of charge by the joint Cambridge Crystallographic Data Centre and Fachinformationszentrum Karlsruhe Access Structures service.[10]

Table S1 *Experimental details of single crystal X-ray diffraction studies, continued*

**Compound**	**(18_2R_5S_7S)**	**(18_inversion_twin)**	**(15)**
Crystal data			
Chemical formula	$C_{29}H_{39}Cl_2NORu$	$C_{29}H_{39}Cl_2NORu$	$C_{31}H_{44}Cl_2N_2ORu\cdot CF_3O_3S\cdot CH_2Cl_2$
M_r	589.58	589.58	866.65
Crystal system, space group	Orthorhombic, $P2_12_12_1$	Orthorhombic, $P2_12_12_1$	Orthorhombic, *Pbca*
Temperature (K)	150		
a, *b*, *c* (Å)	9.7846 (12), 16.3964 (17), 17.076 (2)	9.7881 (4), 16.3946 (6), 17.0819 (7)	16.2635 (12), 16.6893 (15), 28.446 (3)
V (Å^3)	2739.5 (6)	2741.17 (19)	7721.0 (12)
Z	4	4	8
Radiation type	Mo *K*α		
μ (mm^{-1})	0.79	0.79	0.79
Crystal size (mm)	0.31 × 0.27 × 0.10	0.24 × 0.14 × 0.06	0.30 × 0.13 × 0.08
Data collection			
Diffractometer	Bruker D8 VENTURE		
Absorption correction	Multi-scan *SADABS2016*/2 - Bruker AXS area detector scaling and absorption correction		
T_{min}, T_{max}	0.87, 0.93	0.88, 0.95	0.85, 0.94
No. of measured, independent and ó observed [$I > 2\sigma(I)$] reflections	119890, 5618, 5345	137576, 5619, 5392	91903, 7300, 5414
R_{int}	0.084	0.076	0.085
$(\sin \theta/\lambda)_{max}$ (Å^{-1})	0.626	0.626	0.609
Refinement			
$R[F^2 > 2\sigma(F^2)]$, $wR(F^2)$, S	0.033, 0.097, 1.24	0.036, 0.104, 1.24	0.057, 0.166, 1.08
No. of reflections	5618	5619	7300
No. of parameters	316	316	443
H-atom treatment	H-atom parameters constrained		
	$w = 1/[\sigma^2(F_o^2) + (0.0488P)^2 + 1.777P]$ where $P = (F_o^2 + 2F_c^2)/3$	$w = 1/[\sigma^2(F_o^2) + (0.0514P)^2 + 3.1822P]$ where $P = (F_o^2 + 2F_c^2)/3$	$w = 1/[\sigma^2(F_o^2) + (0.0671P)^2 + 36.9188P]$ where $P = (F_o^2 + 2F_c^2)/3$
$\Delta\rangle_{max}$, $\Delta\rangle_{min}$ (e Å^{-3})	1.39, -0.74	1.42, -0.78	2.21, -1.30
Absolute structure	Flack x determined using 2265 quotients [(I+)-(I-)]/[(I+)+(I-)] (Parsons, Flack and Wagner, Acta Cryst. B69 (2013) 249-259).	Flack x determined using 2275 quotients [(I+)-(I-)]/[(I+)+(I-)] (Parsons, Flack and Wagner, Acta Cryst. B69 (2013) 249-259).	–
Absolute structure parameter	0.026 (13)	0.500 (9)	–

Table S2 *Selected Geometric parameters (Å, º) of ruthenium coordination*

**Compound**	**14**	**18**	**5**	**20**
C_c — Ru	1.941 (8)<br>1.927 (8)	1.962 (9)	1.929 (9)<br>1.936 (9)<br>1.921 (9)	2.140 (4)<br>2.123 (4)
C_{bz} — Ru	1.818 (8)<br>1.817 (8)	1.822 (9)	1.832 (9)<br>1.828 (9)<br>1.842 (9)	1.829 (4)
O — Ru	2.374 (6)<br>2.363 (6)	2.358 (5)	2.372 (6)<br>2.368 (5)<br>2.354 (5)	-
Cl — Ru	2.340 (2)<br>2.354 (3)<br>2.344 (3)<br>2.332 (3)	2.339 (3)<br>2.345 (3)	2.340 (3)<br>2.343 (3)<br>2.343 (3)<br>2.337 (3)<br>2.346 (3)<br>2.349 (3)	2.3945 (10)<br>2.4089 (10)
Cl — Ru — Cl	157.56 (11)<br>157.11 (11)	157.68 (12)	154.32 (11)<br>154.68 (10)<br>155.01 (10)	165.09 (4)
C_{bz} — Ru — C	104.9 (4)<br>104.4 (4)	104.7 (4)	104.5 (4)<br>104.3 (3)<br>104.1 (3)	98.6 (18)
Ru — Plane (Cl-Cl-C)	0.218<br>0.224	0.204	0.221<br>0.227<br>0.232	0.001

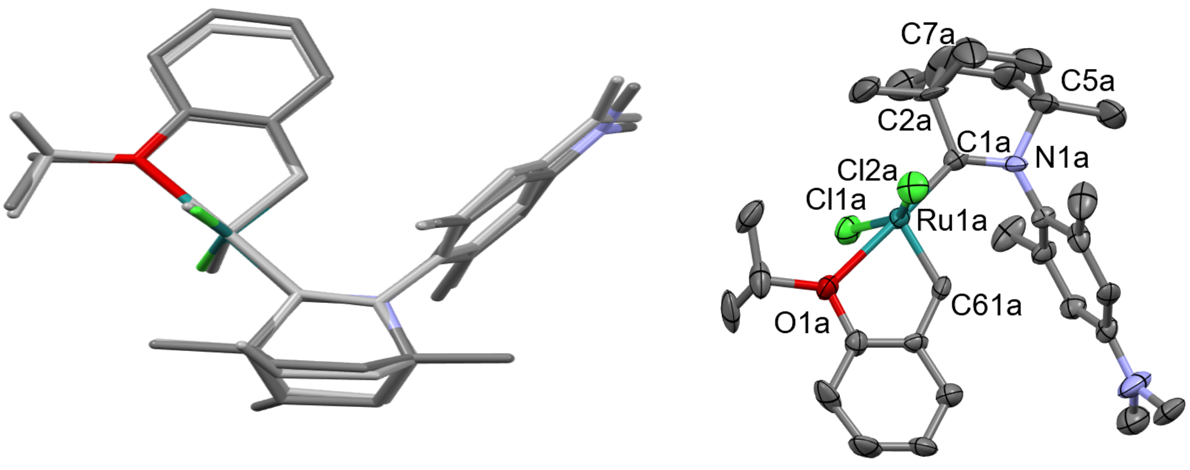

Fig. S43. Overlay of two isomers in the asymmetric unit of **14**, (left) and ORTEP view of one of the enantiomers with partial numbering scheme (right). The relative configurations are C2aS, C5aR, C7aR and C2bR, C5bS, C7bS, polar space group.

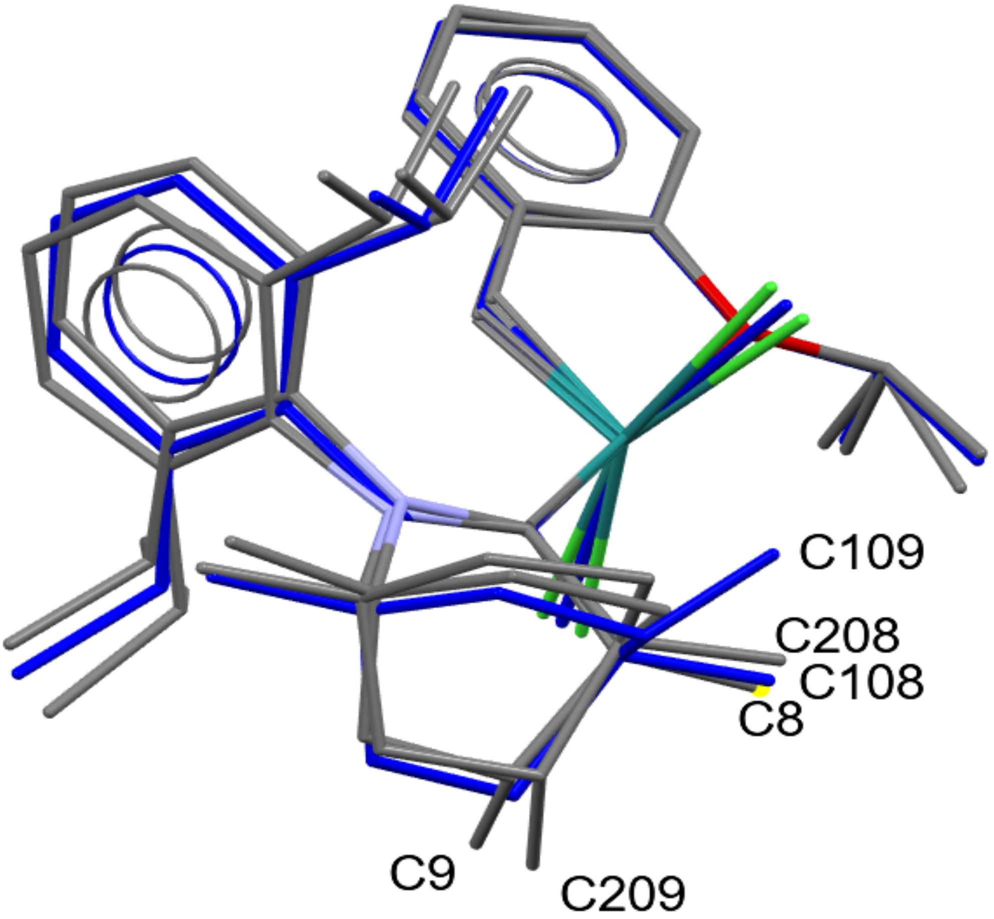

Fig. S44. Overlay of three conformers of the asymmetric unit of **5**. Stick model, hydrogen atoms are omitted for clarity. The opposite orientation of C109 than the corresponding C9 and C209 is shown. The relative assignment is *rac*-(C2S*, C3R*, C5R*, C102R*, C103S*, C105S*, C202S*, C203R*, C205R*, centrosymmetric space group.

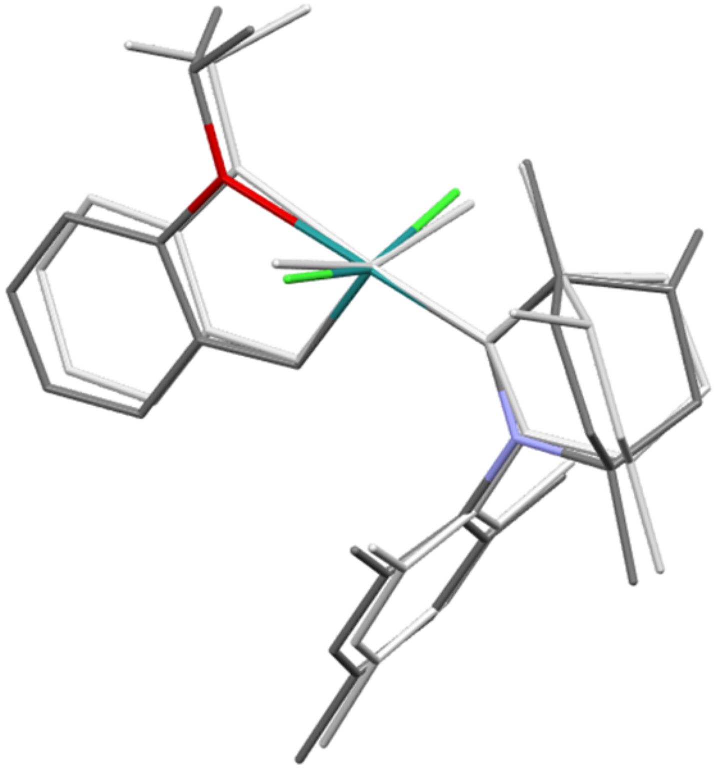

Fig. S45. Overlay of two enantiomers of **18** determined from two different enantiopure crystals with Flack parameters of 0 within 3σ

5. References

1. Sheldrick, G. M. & IUCr. A short history of SHELX. *urn:issn:0108-7673* **64**, 112–122 (2007).
2. Westrip, S. P. publCIF: software for editing, validating and formatting crystallographic information files. *urn:issn:0021-8898* **43**, 920–925 (2010).
3. Macrae, C. F. *et al.* Mercury: visualization and analysis of crystal structures. *urn:issn:0021-8898* **39**, 453–457 (2006).
4. Spek, A. L. Single-crystal structure validation with the program PLATON. *urn:issn:0021-8898* **36**, 7–13 (2003).
5. Tomás-Mendivil, E. *et al.* Bicyclic (Alkyl)(amino)carbenes (BICAACs): Stable Carbenes More Ambiphilic than CAACs. *J. Am. Chem. Soc.* **139**, 7753–7756 (2017).
6. Nagyházi, M. *et al.* Synthesis and characterization of novel PEPPSI type bicyclic (alkyl)(amino)carbene (BICAAC)-Pd complexes. *J. Mol. Struct.* **1256**, 132483 (2022).
7. Nagyházi, M. *et al.* Towards Sustainable Catalysis – Highly Efficient Olefin Metathesis in Protic Media Using Phase Labelled Cyclic Alkyl Amino Carbene (CAAC) Ruthenium Catalysts. *ChemCatChem* **12**, 1953–1957 (2020).
8. Gawin, R., Kozakiewicz, A., Guńka, P. A., Dąbrowski, P. & Skowerski, K. Bis(Cyclic Alkyl Amino Carbene) Ruthenium Complexes: A Versatile, Highly Efficient Tool for Olefin Metathesis. *Angew. Chemie Int. Ed.* **56**, 981–986 (2017).
9. Samkian, A. E., Xu, Y., Virgil, S. C., Yoon, K. Y. & Grubbs, R. H. Synthesis and Activity of Six-Membered Cyclic Alkyl Amino Carbene-Ruthenium Olefin Metathesis Catalysts. *Organometallics* **39**, 495–499 (2020).
10. Groom, C. R., Bruno, I. J., Lightfoot, M. P. & Ward, S. C. The Cambridge Structural Database. *urn:issn:2052-5206* **72**, 171–179 (2016).

Author Contributions

Márton Nagyházi: concept (ligand and catalyst development), synthesis of ligands and complexes. Ádám Lukács: synthesis of ligands and complexes, preliminary catalysis investigation. Gábor Turczel: catalysis investigation. Jenő Hancsók: concept support. József Valyon: concept support. Sándor Kéki: HRMS investigations and kinetics. Attila Bényei: XRD measurements and discussion. Róbert Tuba: concept (ligand and catalyst development, catalysis), general management, paper writing.
